# Identifying major predictors for parenting stress in a caregiver of autism spectrum disorder using machine learning models

**DOI:** 10.3389/fnins.2023.1229155

**Published:** 2023-08-29

**Authors:** Hangnyoung Choi, Jae Han Kim, Hwiyoung Kim, Keun-Ah Cheon

**Affiliations:** ^1^Department of Child and Adolescent Psychiatry, Severance Hospital, Yonsei University College of Medicine, Seoul, Republic of Korea; ^2^Institute of Behavioral Science in Medicine, Yonsei University College of Medicine, Yonsei University Health System, Seoul, Republic of Korea; ^3^Yonsei University College of Medicine, Severance Hospital, Yonsei University Health System, Seoul, Republic of Korea; ^4^Center of Clinical Imaging Data Science, Department of Radiology, Yonsei University College of Medicine, Seoul, Republic of Korea; ^5^Department of Biomedical System Informatics, Yonsei University College of Medicine, Seoul, Republic of Korea

**Keywords:** autism spectrum disorder, parenting stress, artificial intelligence, machine learning, predictor

## Abstract

**Introduction:**

Previous studies have investigated predictive factors for parenting stress in caregivers of autism spectrum disorder (ASD) patients using traditional statistical approaches, but their study settings and results were inconsistent. Herein, this study aimed to identify major predictors for parenting stress in this population by developing explainable machine learning models.

**Methods:**

Study participants were collected from the Department of Child and Adolescent Psychiatry, Severance Hospital, Yonsei University College of Medicine, Seoul, the Republic of Korea between March 2016 and October 2020. A total of 36 model features were used, which include subscales of the Minnesota Multiphasic Personality Inventory-2 (MMPI-2) for caregivers’ psychopathology, Social Responsiveness Scale-2 for core symptoms, and Child Behavior Checklist (CBCL) for behavioral problems. Machine learning classifiers [eXtreme Gradient Boosting (XGBoost), random forest (RF), logistic regression, and support vector machine (SVM) classifier] were generated to predict severe total parenting stress and its subscales (parental distress, parent-child dysfunctional interaction, and difficult child). Model performance was assessed by area under the receiver operating curve (AUC), sensitivity, specificity, accuracy, positive predictive value, and negative predictive value. We utilized the SHapley Additive exPlanations tree explainer to investigate major predictors.

**Results:**

A total of 496 participants were included [mean age of ASD patients 6.39 (SD 2.24); 413 men (83.3%)]. The best-performing models achieved an AUC of 0.831 (RF model; 95% CI 0.740–0.910) for parental distress, 0.814 (SVM model; 95% CI 0.720–0.896) for parent-child dysfunctional interaction, 0.813 (RF model; 95% CI 0.724–0.891) for difficult child, and 0.862 (RF model; 95% CI 0.783–0.930) for total parenting stress on the test set. For the total parenting stress, ASD patients’ aggressive behavior and anxious/depressed, and caregivers’ depression, social introversion, and psychasthenia were the top 5 leading predictors.

**Conclusion:**

By using explainable machine learning models (XGBoost and RF), we investigated major predictors for each subscale of the parenting stress index in caregivers of ASD patients. Identified predictors for parenting stress in this population might help alert clinicians whether a caregiver is at a high risk of experiencing severe parenting stress and if so, providing timely interventions, which could eventually improve the treatment outcome for ASD patients.

## 1. Introduction

Autism spectrum disorder (ASD) is one of the neurodevelopmental disorders that is characterized by two core symptoms: difficulties with social communication and interaction and the presence of repetitive and restricted behaviors or interests (American Psychiatric Association, 2013). Parents of ASD patients were found to experience greater parenting stress than typically developing individuals and even other disabilities (Hayes and Watson, 2013). It is an important issue because high-level parenting stress is associated with the lower effectiveness of parent-mediated intervention (Osborne et al., 2008). Therefore, helping stressed parents can be beneficial in improving the outcome of treatment for ASD patients.

Numerous studies have explored associated factors for parenting stress in caregivers of ASD patients, and personality traits and mood problems of caregivers (Falk et al., 2014; Leonardi et al., 2021), ASD core symptoms (Miranda et al., 2019; Scibelli et al., 2021), and behavioral problems of ASD patients (Yorke et al., 2018; Miranda et al., 2019; Siu et al., 2019; Scibelli et al., 2021) were found to be significantly linked with parenting stress. Recently, associated factors for each dimension of parenting stress (parental distress, parent-child dysfunctional interaction, and difficult child) were investigated (Mello et al., 2022). However, previous studies have utilized different combinations of study variables and statistical tests, leading to inconsistent results and difficult interpretations (Voliovitch et al., 2021; Mello et al., 2022). Moreover, some studies did not address the study variables at the same level; for example, the total score was used for ASD core symptoms, but subscales for behavioral problems (Siu et al., 2019; Mello et al., 2022). Lastly, none of them have attempted to apply machine learning (ML) methods which offer distinct advantages over traditional approaches since ML can handle multi-dimensional and non-linear relationships (Schwalbe and Wahl, 2020).

Herein, our study aimed at identifying predictive features for parenting stress (parental distress, parent-child dysfunctional interaction, difficult child, and total parenting stress) in caregivers of ASD patients by developing explainable ML models. Additionally, we included only subscales of the Minnesota Multiphasic Personality Inventory-2 (MMPI-2) for caregivers’ psychopathology (Graham, 1990; Han et al., 2006), Social Responsiveness Scale-2 (SRS-2) for ASD core symptoms (Constantino, 2012; Chun et al., 2021), Child Behavior Checklist (CBCL) for behavioral problems (Han and Yoo, 1995; Achenbach, 1999), and other additional features as model features. We expected that the identified predictive features would help alert clinicians to whether a caregiver is at a high risk of severe parenting stress and provide timely interventions to stressful parents, which would eventually enhance the treatment outcome for ASD patients.

## 2. Materials and methods

**Figure 1:**
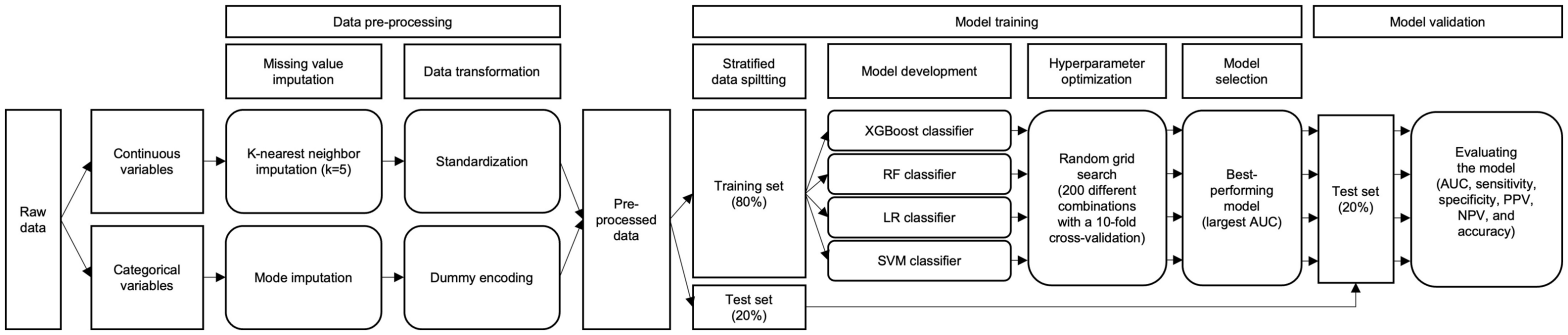
Overall process of the ML models. AUC, area under the receiver operating curve; LR, logistic regression; ML, machine learning; NPV, negative predictive value; PPV, positive predictive value; RF, random forest; SVM, support vector machine; XGBoost, eXtreme Gradient Boosting.

We followed the STROBE guideline (Supplementary material, pp. 6–7) (von Elm et al., 2007). The present study was approved by the Institutional Review Board of the Severance Hospital of Yonsei University, Seoul, the Republic of Korea. Informed consent was waived since we used retrospective and deidentified patient data (IRB number: 4-2022-0803). The overall process of ML models is displayed in Figure 1.

### 2.1. Participants recruitment

Study participants were retrospectively collected from the Department of Child and Adolescent Psychiatry, Severance Hospital, Yonsei University College of Medicine, Seoul, the Republic of Korea, between March 2016 and October 2020. Child and adolescent psychiatrists conducted a semi-structured interview to confirm ASD based on DSM-5. Patients under 19 who were identified as having ASD and their primary caregiver were included.

Patients under the following conditions were excluded: those who did not report SRS-2, CBCL, or MMPI-2; those who had organic brain diseases (e.g., epilepsy, encephalitis, and demyelinating disease); and those who had a comorbid mental disorder (e.g., bipolar and related disorders and schizophrenia spectrum and other psychotic disorders).

### 2.2. Outcome variables

The outcome of interest was “parenting stress” in the primary caregiver of an ASD patient. Parenting stress was assessed by the Parenting Stress Index-Short Form (PSI-SF), which contained 36 items (Abidin, 1990; Lee et al., 2008). A total of four scales (parental distress, parent-child dysfunctional interaction, difficult child, and total parenting stress) were set as outcome variables for prediction. As the models were designed to distinguish those with severe and mild-to-moderate levels of parenting stress, we established the threshold for severity at the 80th percentile following the formal documentation (Abidin, 1990).

### 2.3. Model features

Our dataset included a total of 36 variables which are listed in Table 1 as model features. Both SRS-2 and CBCL were rated by a caregiver. We noted that subscales of the CBCL were different by age (1.5–5 versus 6–18); hence we used only common ones (anxious/depressed, aggressive behavior, attention problems, somatic complaints, withdrawn, and other problems) when using the overall sample. The definitions of each model feature are provided in Supplementary material, pp. 8–10.

**Table 1:** The list of model features.

Caregivers variables	- Assistant caregiver status (yes or no) - Current working status (yes or no) - The existence of another child with a mental disorder (yes or no) - Age of caregiver - The order of birth - The number of children - *T*-score of MMPI-2 clinical scales (hypochondriasis, depression, hysteria, psychopathy, masculinity/femininity, paranoia, psychasthenia, schizophrenia, hypomania, and social introversion)
ASD patients variables	- Sex (male or female) - Family history of mental disorder (yes or no) - History of the major disease (yes or no) - Psychotropic medication status (drug-free, monotherapy of antipsychotics, combined therapy of antipsychotics, or other psychotic medication) - Mode of delivery (vaginal delivery or cesarean section) - Age of the patient - Gestational age - Birth weight - Full-scale intelligence quotient - *T*-score of SRS-2 subscales (social awareness, social cognition, social communication, social motivation, and autistic mannerisms) - *T*-score of CBCL syndrome subscales (anxious/depressed, aggressive behavior, attention problems, somatic complaints, withdrawn, and other problems)

CBCL, Child Behavior Checklist; MMPI, Minnesota Multiphasic Personality Inventory; SRS, Social Responsiveness Scale.

### 2.4. Data pre-processing

Missing value imputation was performed using *k*-nearest neighbor imputation with *k* = 5 for continuous features and mode imputation for categorical features. Then, continuous features were standardized to address the potential multicollinearity amongst included variables and categorical features were transformed into dummy variables. Items with missing values and their portion are provided in Supplementary material, pp. 11–12.

### 2.5. Model development

The datasets were randomly partitioned into two groups: a training set (80%) and a test set (20%). To avoid data shifting between two subsets, random data split was stratified with respect to the outcome variable. Four supervised ML classifiers—eXtreme Gradient Boosting (XGBoost), random forest (RF), logistic regression, and support vector machine (SVM) classifier—were generated for each outcome (parental distress, parent-child dysfunctional interaction, difficult child, and total parenting stress), that is, 16 models in total were developed. Hyperparameter optimization was performed by random grid search of 200 different combinations with 10-fold cross-validation (Bergstra and Bengio, 2012). We assessed the model performance with the area under the receiver operating curve (AUC) and selected the best-performing model (i.e., the model that presented the largest AUC). Then, we validated the model with the remaining 20% test set.

We performed the subgroup analyses for different forms of CBCL (1.5–5 and 6–18) and comorbid attention-deficit/hyperactivity disorder (ADHD) status (with and without ADHD). We utilized the SHapley Additive exPlanations (SHAP) tree explainer method for RF and XGBoost classifiers to investigate major predictors (Lundberg et al., 2020).

### 2.6. Statistical analysis

We utilized the *t*-test for continuous variables (e.g., age, subscales of CBCL, SRS-2, and MMPI-2) and the *χ*^2^ test for categorical variables (e.g., sex, assistant caregiver status, and psychotropic medication status) to assess statistical differences of included variables between subgroups. Multicollinearity refers to a condition in which two or more variables show a strong correlation, which can be problematic in some ML models since it hinders the ability of models to distinguish their individual impacts on the dependent variable. We calculated variance inflation factors for each continuous variable in each sample to detect whether multicollinearity exists (Neter et al., 1996). Conventionally, a variance inflation factor greater than 5 is considered indicative of a problematic level of multicollinearity. Model performance was assessed by AUC, sensitivity, specificity, positive predictive value, negative predictive value, and accuracy. The formulas for each metric are displayed in Supplementary material, p. 13. The 95% confidence intervals (CIs) for each estimate were obtained using a bootstrap of 10,000 resamples. Bootstrap is a statistical method that involves drawing multiple random samples with replacements from the original data to create new datasets, allowing us to estimate the uncertainty related to a point estimate.

Statistical analyses were two-tailed, and *p* < 0.05 was deemed to indicate statistical significance. Statistical analyses were performed using R software (version 4.1.3), and all ML models were implemented using Python (version 3.8.1).

## 3. Results

### 3.1. Study dataset

A total of 496 ASD patients and their caregivers were included [mean age of ASD patients 6.39 (standard deviation 2.24); 413 men (83.3%)]. Detailed participants’ information is displayed in Table 2. None of the included variables showed significant multicollinearity (Supplementary material, p. 14).

**Table 2:** Sample information.

	Overall sample, mean (SD) (*n* = 496)	CBCL, mean (SD)			ADHD, mean (SD)		
		CBCL 1.5–5 (*n* = 194)	CBCL 6–18 (*n* = 302)	*p* ^a^	With ADHD (*n* = 168)	Without ADHD (*n* = 328)	*p* ^a^
Age of caregiver	39.04 (4.31)	37.23 (3.96)	40.19 (4.12)	<0.001	39.66 (4.35)	38.72 (4.25)	0.029
*Assistant caregiver status*							
Yes (%)	106 (21.4)	58 (29.9)	48 (15.9)	<0.001	30 (17.9)	76 (23.2)	0.185
No (%)	390 (78.6)	136 (70.1)	254 (84.1)		138 (82.1)	252 (76.8)	
*Current working status*							
Yes (%)	188 (37.9)	74 (38.1)	114 (37.7)	0.916	63 (37.5)	125 (38.1)	0.926
No (%)	308 (62.1)	120 (61.9)	188 (62.3)		105 (62.5)	203 (61.9)	
The order of birth	1.26 (0.47)	1.30 (0.49)	1.23 (0.45)	0.08	1.20 (0.43)	1.29 (0.49)	0.036
Number of children	1.71 (0.62)	1.68 (0.60)	1.73 (0.63)	0.349	1.77 (0.63)	1.67 (0.61)	0.088
*Existence of another child with mental disorder*							
Yes (%)	17 (3.4)	9 (4.6)	8 (2.6)	0.353	8 (4.8)	9 (2.7)	0.357
No (%)	479 (96.6)	185 (95.4)	294 (97.4)		160 (95.2)	319 (97.3)	
*MMPI (T-score)*							
Hypochondriasis (code 1)	50.49 (7.72)	49.63 (7.31)	51.04 (7.94)	0.047	51.67 (8.08)	49.88 (7.47)	0.015
Depression (code 2)	56.86 (11.24)	55.80 (10.71)	57.53 (11.54)	0.095	58.29 (11.19)	56.12 (11.22)	0.043
Hysteria (code 3)	51.21 (8.32)	50.02 (7.50)	51.98 (8.74)	0.01	52.12 (8.69)	50.75 (8.10)	0.083
Psychopathy (code 4)	50.50 (9.97)	49.00 (8.69)	51.46 (10.61)	0.007	51.45 (10.15)	50.01 (9.85)	0.127
Masculinity/Femininity (code 5)	45.39 (8.85)	45.78 (9.27)	45.14 (8.58)	0.431	45.04 (8.70)	45.56 (8.94)	0.535
Paranoia (code 6)	47.95 (8.13)	47.78 (7.69)	48.06 (8.41)	0.712	47.64 (8.82)	48.11 (7.76)	0.545
Psychasthenia (code 7)	50.96 (10.23)	50.43 (9.92)	51.29 (10.43)	0.358	51.65 (10.99)	50.60 (9.82)	0.281
Schizophrenia (code 8)	47.17 (8.21)	46.73 (7.58)	47.45 (8.58)	0.341	47.40 (8.19)	47.04 (8.22)	0.642
Hypomania (code 9)	44.91 (8.53)	45.57 (8.36)	44.49 (8.62)	0.169	44.43 (8.22)	45.16 (8.69)	0.37
Social Introversion (code 0)	52.38 (11.05)	51.80 (10.72)	52.76 (11.26)	0.346	52.83 (11.05)	52.16 (11.06)	0.522
Age of the patient	6.39 (2.24)	4.35 (0.80)	7.70 (1.86)	<0.001	7.48 (2.18)	5.83 (2.05)	<0.001
*Sex*							
Male (%)	413 (83.3)	159 (82.0)	254 (84.1)	0.616	147 (87.5)	266 (81.1)	0.093
Female (%)	83 (16.7)	35 (18.0)	48 (15.9)		21 (12.5)	62 (18.9)	
*Family history of mental disorders*							
Yes (%)	94 (19.0)	35 (18.0)	59 (19.5)	1	31 (18.5)	63 (19.2)	0.826
No (%)	402 (82.0)	159 (82.0)	243 (80.5)		137 (81.5)	265 (80.8)	
*History of major disease*							
Yes (%)	71 (14.3)	23 (11.9)	48 (15.9)	0.275	22 (13.1)	49 (14.9)	0.654
No (%)	425 (85.7)	171 (88.1)	254 (84.1)		146 (86.9)	279 (85.1)	
*Psychotropic medication status*							
Drug free (%)	412 (83.1)	179 (92.3)	233 (77.2)	<0.001	120 (71.4)	292 (89.0)	<0.001
Monotherapy of antipsychotics (%)	60 (12.1)	15 (7.7)	45 (14.9)		29 (17.3)	31 (9.5)	
Combined therapy of antipsychotics (%)	8 (1.6)	0 (0.0)	8 (2.6)		6 (3.6)	2 (0.6)	
Other psychotic medication (%)	16 (3.2)	0 (0.0)	16 (5.3)		13 (7.7)	3 (0.9)	
Gestational age	38.97 (2.13)	39.20 (1.97)	38.82 (2.22)	0.063	38.97 (1.81)	38.97 (2.27)	0.99
Birth weight	3.17 (0.49)	3.19 (0.52)	3.16 (0.47)	0.493	3.18 (0.39)	3.17 (0.53)	0.757
*Mode of delivery*							
Vaginal delivery (%)	289 (58.3)	116 (59.8)	173 (57.3)	0.505	97 (57.7)	192 (58.5)	1
Caesarean section (%)	207 (41.7)	78 (40.2)	129 (42.7)		71 (42.3)	136 (41.5)	
FSIQ	73.43 (20.30)	64.28 (17.98)	79.12 (19.60)	<0.001	82.77 (18.48)	68.60 (19.53)	<0.001
*SRS (T-score)*							
Social awareness	61.81 (14.08)	63.72 (14.50)	60.58 (13.69)	0.015	60.66 (12.64)	62.39 (14.75)	0.195
Social cognition	66.91 (13.55)	67.04 (12.13)	66.83 (14.41)	0.87	64.94 (13.54)	67.92 (13.46)	0.02
Social communication	76.84 (17.71)	77.76 (16.53)	76.25 (18.43)	0.352	73.55 (15.66)	78.52 (18.47)	0.003
Social motivation	69.42 (16.35)	71.29 (16.16)	68.21 (16.39)	0.04	66.10 (14.56)	71.12 (16.97)	0.001
Autistic mannerisms	77.96 (17.86)	74.56 (17.21)	80.15 (17.96)	0.001	77.73 (16.95)	78.08 (18.34)	0.837
*CBCL (T-score)*							
Anxious/depressed	59.27 (9.38)	55.10 (7.30)	61.95 (9.60)	<0.001	61.40 (9.11)	58.17 (9.34)	<0.001
Aggressive behavior	63.53 (9.65)	65.28 (8.70)	62.41 (10.07)	0.001	61.38 (9.79)	64.64 (9.40)	<0.001
Attention problems	56.28 (7.28)	54.91 (6.30)	57.16 (7.73)	0.001	56.86 (7.56)	55.98 (7.13)	0.199
Somatic complaints	64.26 (9.53)	61.29 (7.74)	66.16 (10.07)	<0.001	66.71 (9.77)	63.00 (9.16)	<0.001
Withdrawn	60.48 (8.74)	58.36 (7.61)	61.85 (9.15)	<0.001	62.79 (9.32)	59.30 (8.19)	<0.001
Other problems	61.24 (8.16)	61.79 (8.04)	60.89 (8.22)	0.23	62.26 (7.68)	60.72 (8.36)	0.046
*CBCL 1.5–5 (T-score)*							
Emotionally reactivity		57.51 (8.19)					
Sleep problems		55.23 (7.58)					
*CBCL 6–18 (T-score)*							
Social problems			67.93 (7.86)				
Thought problems			63.79 (8.15)				
Rule-breaking behavior			60.64 (7.15)				
*PSI*							
Parental distress	59.35 (28.47)	60.08 (29.26)	58.88 (27.99)	0.646	61.84 (25.94)	58.07 (29.64)	0.163
Parent-child dysfunctional interaction	69.35 (28.82)	67.32 (27.61)	70.66 (29.53)	0.209	69.51 (28.98)	69.27 (28.77)	0.933
Difficult child	71.50 (24.76)	68.18 (24.47)	73.64 (24.75)	0.016	74.17 (23.85)	70.14 (25.14)	0.087
Total parenting stress	70.09 (27.16)	68.03 (25.99)	71.41 (27.85)	0.176	72.05 (26.13)	69.09 (27.66)	0.25

ADHD, attention-deficit/hyperactivity disorder; CBCL, Child Behavior Checklist; FSIQ, full scale intelligence quotient; MMPI, Minnesota Multiphasic Personality Inventory; PSI, parenting stress index; SD, standard deviation; SRS, social responsiveness scale.

a *p*-value for group difference and *p* < 0.05 indicates statistically significant between-group difference.

### 3.2. Model performance and major predictors

**Figure 2:**
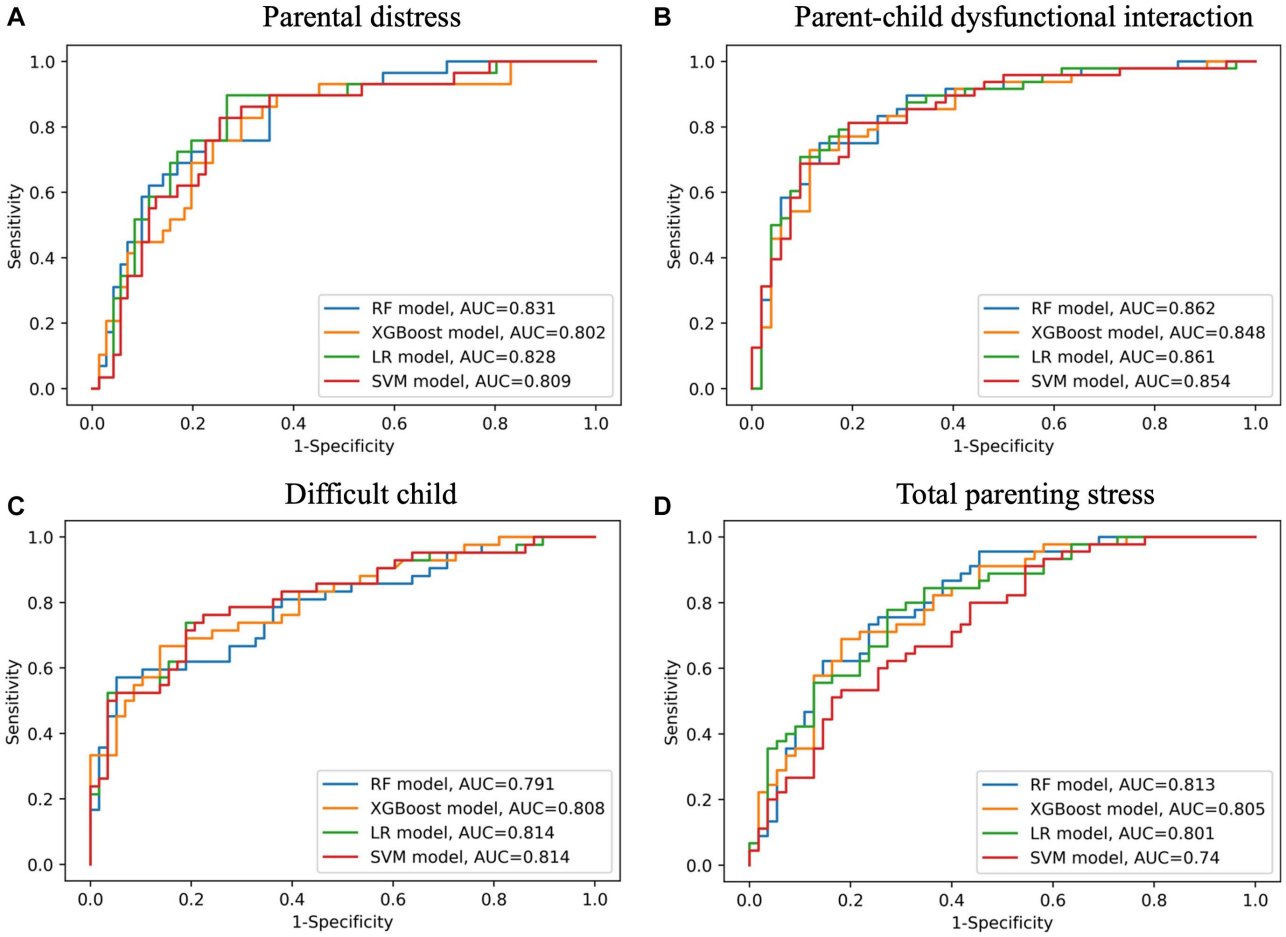
Receiver operating characteristic curves on the test set for subscales of parenting stress index (**A** - parental distress, **B** - parent-child dysfunctional interaction, **C** - difficult child, **D** - total parenting stress). AUC, area under curve; LR, logistic regression; RF, random forest; SVM, support vector machine; XGBoost, eXtreme Gradient Boosting.

Among the total 496 participants, 396 (80%) were assigned to the training set and 100 (20%) to the test set. Receiver operating characteristic curves for parental distress, parent-child dysfunctional interaction, difficult child, and total parenting stress for the test set are presented in Figure 2.

**Figure 3:**
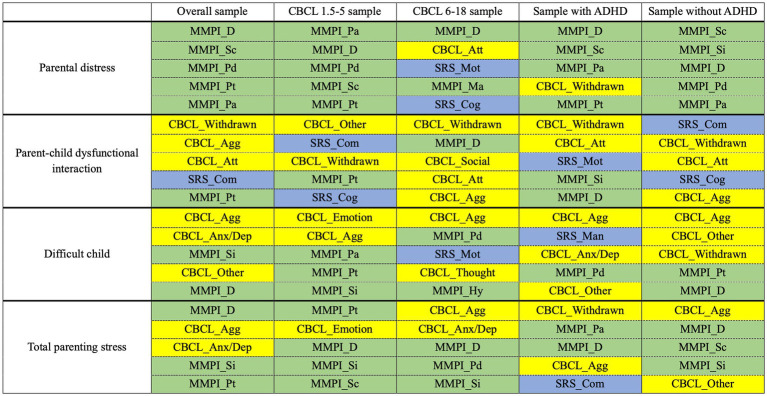
Top 5 predictors for each outcome based on the tested samples. Results of the model (RF or XGBoost model) with the higher AUC on test set were presented. To enhance visibility, we applied color-coding to the cells according to the following scheme: blue for SRS-2 subscales, green for MMPI-2 clinical scales, and yellow for CBCL syndrome subscales. ADHD, attention-deficit/hyperactivity disorder; AUC, area under the receiver operating curve; CBCL, Child Behavior Checklist; CBCL_Agg, aggressive behavior; CBCL_Anx/Dep, anxious/depressed; CBCL_Att, attention problems; CBCL_Emotion, emotionally reactivity; CBCL_Other, other problems; CBCL_Social, social problems; CBCL_Thought, thought problems; CBCL_Withdrawn, withdrawn; MMPI, Minnesota Multiphasic Personality Inventory; MMPI_D, depression (code 2); MMPI_Hy, hysteria (code 3); MMPI_Ma, hypomania (code 9); MMPI_Pa, paranoia (code 6); MMPI_Pd, psychopathy (code 4); MMPI_Pt, psychasthenia (code 7); MMPI_Sc, schizophrenia (code 8); MMPI_Si, social introversion (code 0); SRS, social responsiveness scale; SRS_Cog, social cognition; SRS_Com, social communication; SRS_Man, autistic mannerisms; SRS_Mot, social motivations.

For predicting parental distress, the RF model (AUC 0.831, 95% CI 0.740–0.910; sensitivity 0.655, 95% CI 0.478–0.821; specificity 0.859, 95% CI 0.773–0.934) outperformed the other models on the test set. The top 5 predictors of the RF model were caregivers’ MMPI-2 scores of depression, schizophrenia, psychopathy, psychasthenia, and paranoia (Table 3 and Figure 3).

**Table 3:** Model performances on the test set with the top 10 major predictors for subscales of parenting stress index (*n* = 496).^a^

	Parental distress				Parent-child dysfunctional interaction				Difficult child				Total parenting stress			
	RF model	XGBoost model	LR model	SVM model	RF model	XGBoost model	LR model	SVM model	RF model	XGBoost model	LR model	SVM model	RF model	XGBoost model	LR model	SVM model
ROC AUC	0.831	0.802	0.828	0.809	0.791	0.808	0.814	0.814	0.813	0.805	0.801	0.740	0.862	0.848	0.861	0.854
Sensitivity	0.655	0.483	0.586	0.552	0.595	0.667	0.619	0.595	0.733	0.711	0.600	0.578	0.708	0.729	0.792	0.708
Specificity	0.859	0.859	0.845	0.887	0.828	0.845	0.828	0.828	0.745	0.782	0.782	0.745	0.865	0.865	0.827	0.808
PPV	0.655	0.583	0.607	0.667	0.714	0.757	0.722	0.714	0.702	0.727	0.692	0.650	0.829	0.833	0.809	0.773
NPV	0.859	0.803	0.833	0.829	0.738	0.778	0.750	0.738	0.774	0.768	0.705	0.683	0.763	0.776	0.811	0.750
Accuracy	0.800	0.750	0.770	0.790	0.730	0.770	0.740	0.730	0.740	0.750	0.700	0.670	0.790	0.800	0.810	0.760
Top 10 predictors (ordered by importance)^b^	MMPI_D MMPI_Sc MMPI_Pd MMPI_Pt MMPI_Pa				CBCL_Withdrawn CBCL_Agg CBCL_Att SRS_Com MMPI_Pt				CBCL_Agg CBCL_Anx/Dep MMPI_Si CBCL_Other MMPI_D				MMPI_D CBCL_Agg CBCL_Anx/Dep MMPI_Si MMPI_Pt			
	CBCL_Other MMPI_Si MMPI_Ma SRS_Cog CBCL_Att				MMPI_D SRS_Cog MMPI_Pd FSIQ MMPI_Si				SRS_Man CBCL_Att SRS_Cog CBCL_Withdrawn Age of caregiver				CBCL_Att MMPI_Sc CBCL_Withdrawn SRS_Mot CBCL_Other			

AUC, area under the curve; CBCL, Child Behavior Checklist; CBCL_Agg, aggressive behavior; CBCL_Anx/Dep, anxious/depressed; CBCL_Att, attention problems; CBCL_Other, other problems; CBCL_Withdrawn, withdrawn; FSIQ, full scale intelligence quotient; LR, logistic regression; MMPI, Minnesota Multiphasic Personality Inventory; MMPI_D, depression (code 2); MMPI_Ma, hypomania (code 9); MMPI_Pa, paranoia (code 6); MMPI_Pd, psychopathy (code 4); MMPI_Pt, psychasthenia (code 7); MMPI_Sc, schizophrenia (code 8); MMPI_Si, social introversion (code 0); NPV, negative predictive value; PPV, positive predictive value; RF, random forest; ROC, receiver operating characteristic; SRS, social responsiveness scale; SRS_Cog, social cognition; SRS_Com, social communication; SRS_Man, autistic mannerisms; SRS_Mot, social motivations; SVM, support vector machine; XGBoost, eXtreme Gradient Boosting.

a The 95% confidence intervals for each estimate are presented in Supplementary material, pp. 15–26.

b Results of the model (RF or XGBoost model) with the higher ROC AUC on test set were presented.

For predicting parent-child dysfunctional interaction, the SVM model showed the largest AUC with the narrowest 95% CI (AUC 0.814, 95% CI 0.720–0.896; sensitivity 0.595, 95% CI 0.444–0.744; specificity 0.828, 95% CI 0.724–0.918) on the test set. Among the models providing feature importance, the XGBoost model (AUC 0.808, 95% CI 0.715–0.891; sensitivity 0.667, 95% CI 0.522–0.810; specificity 0.845, 95% CI 0.745–0.933) was the best. The top 5 predictors of the XGBoost model were ASD patients’ CBCL scores of withdrawn, aggressive behavior, and attention problems, ASD patients’ SRS-2 scores of social communication, and caregivers’ MMPI-2 scores of psychasthenia (Table 3 and Figure 3).

For predicting difficult child, the RF model (AUC 0.813, 95% CI 0.724–0.891; sensitivity 0.733, 95% CI 0.600–0.857; specificity 0.745, 95% CI 0.627–0.857) showed the best performance on the test set. The top 5 predictors of the RF model were ASD patients’ CBCL scores of aggressive behavior, anxious/depressed, and other problems and caregivers’ MMPI-2 scores of social introversion and depression (Table 3 and Figure 3).

For predicting total parenting stress, the performance of the RF model (AUC 0.862, 95% CI 0.783–0.930; sensitivity 0.708, 95% CI 0.578–0.833; specificity 0.865, 95% CI 0.764–0.951) was the best on the test set. The top 5 predictors of the RF model were ASD patients’ CBCL scores of aggressive behavior and anxious/depressed, and caregivers’ MMPI-2 scores of depression, social introversion, and psychasthenia (Table 3 and Figure 3).

Detailed results for each estimate on both training and test sets and the SHAP summary plots for RF and XGBoost are provided in Supplementary material, pp. 15–26.

### 3.3. Subgroup analyses

#### 3.3.1. Results of CBCL subgroups

In both subgroups of CBCL 1.5–5 and 6–18, the prediction of parental distress and parent-child dysfunctional interaction was unsuccessful, showing low sensitivity (ranging from 0.000 to 0.679) and high specificity (ranging from 0.606 to 1.000), while the model performances were retained for difficult child and total parenting stress. There seemed to be a difference in the trend of major predictors for total parenting stress between CBCL 1.5–5 and CBCL 6–18. For CBCL 1.5–5, caregivers’ MMPI-2 scores including psychasthenia, depression, social introversion, and schizophrenia were given high priority. However, in the case of CBCL 6–18, ASD patients’ CBCL scores of aggressive behavior and anxious/depressed were shown to be more critical than the caregivers’ MMPI-2 scores (Table 4 and Figure 3).

**Table 4:** Model performances on the test set with the top 10 major predictors in samples subgrouped by CBCL.^a^

		Parental distress				Parent-child dysfunctional interaction				Difficult child				Total parenting stress			
		RF model	XGBoost model	LR model	SVM model	RF model	XGBoost model	LR model	SVM model	RF model	XGBoost model	LR model	SVM model	RF model	XGBoost model	LR model	SVM model
CBCL 1.5–5 sample (*n* = 194)	ROC AUC	0.861	0.889	0.846	0.815	0.714	0.720	0.660	0.686	0.897	0.925	0.811	0.853	0.845	0.810	0.788	0.815
	Sensitivity	0.583	0.417	0.250	0.000	0.429	0.214	0.000	0.000	0.667	0.733	0.333	0.400	0.625	0.625	0.375	0.000
	Specificity	1.000	1.000	1.000	1.000	0.760	0.960	1.000	1.000	0.958	0.917	1.000	0.917	0.870	0.826	0.957	1.000
	PPV	1.000	1.000	1.000	NA	0.500	0.750	NA	NA	0.909	0.846	1.000	0.750	0.769	0.714	0.857	NA
	NPV	0.844	0.794	0.750	0.692	0.704	0.686	0.641	0.641	0.821	0.846	0.706	0.710	0.769	0.760	0.688	0.590
	Accuracy	0.872	0.821	0.769	0.692	0.641	0.692	0.641	0.641	0.846	0.846	0.744	0.718	0.769	0.744	0.718	0.590
	Top 10 predictors (ordered by importance)^b^	MMPI_Pa MMPI_D MMPI_Pd MMPI_Sc MMPI_Pt				CBCL_Other SRS_Com CBCL_Withdrawn MMPI_Pt SRS_Cog				CBCL_Emotion CBCL_Agg MMPI_Pa MMPI_Pt MMPI_Si				MMPI_Pt CBCL_Emotion MMPI_D MMPI_Si MMPI_Sc			
		MMPI_Si SRS_Cog SRS_Man CBCL_Withdrawn CBCL_Att				SRS_Mot MMPI_Ma MMPI_D CBCL_Att CBCL_Sleep				MMPI_D Age of caregiver CBCL_Withdrawn CBCL_Sleep MMPI_Sc				CBCL_Withdrawn CBCL_Agg CBCL_Att MMPI_Pa SRS_Com			
CBCL 6–18 sample (*n* = 302)	ROC AUC	0.803	0.709	0.787	0.770	0.745	0.750	0.720	0.726	0.805	0.814	0.777	0.726	0.847	0.747	0.839	0.842
	Sensitivity	0.412	0.529	0.412	0.412	0.679	0.679	0.571	0.643	0.867	0.867	0.867	0.900	0.812	0.656	0.656	0.656
	Specificity	0.932	0.886	0.818	0.841	0.606	0.606	0.667	0.697	0.548	0.645	0.645	0.484	0.793	0.690	0.828	0.897
	PPV	0.700	0.643	0.467	0.500	0.594	0.594	0.593	0.643	0.650	0.703	0.703	0.628	0.812	0.700	0.808	0.875
	NPV	0.804	0.830	0.783	0.787	0.690	0.690	0.647	0.697	0.810	0.833	0.833	0.833	0.793	0.645	0.686	0.703
	Accuracy	0.787	0.787	0.705	0.721	0.639	0.639	0.623	0.672	0.705	0.754	0.754	0.689	0.803	0.672	0.738	0.770
	Top 10 predictors (ordered by importance)^b^	MMPI_D CBCL_Att SRS_Mot MMPI_Ma SRS_Cog				CBCL_Withdrawn MMPI_D CBCL_Social CBCL_Att CBCL_Agg				CBCL_Agg MMPI_Pd SRS_Mot CBCL_Thought MMPI_Hy				CBCL_Agg CBCL_Anx/Dep MMPI_D MMPI_Pd MMPI_Si			
		MMPI_Pd CBCL_Som CBCL_Other CBCL_Withdrawn CBCL_Agg				SRS_Com SRS_Man CBCL_Other SRS_Cog MMPI_Si				CBCL_Anx/Dep Age of the patient MMPI_D SRS_Man FSIQ				CBCL_Withdrawn SRS_Mot MMPI_Sc SRS_Com CBCL_Thought			

AUC, area under the curve; CBCL, Child Behavior Checklist; CBCL_Agg, aggressive behavior; CBCL_Anx/Dep, anxious/depressed; CBCL_Att, attention problems; CBCL_Emotion, emotionally reactivity; CBCL_Other, other problems; CBCL_Sleep, sleep problems; CBCL_Social, social problems; CBCL_Som, somatic complaints; CBCL_Thought, thought problems; CBCL_Withdrawn, withdrawn; FSIQ, full scale intelligence quotient; LR, logistic regression; MMPI, Minnesota Multiphasic Personality Inventory; MMPI_D, depression (code 2); MMPI_Hy, hysteria (code 3); MMPI_Ma, hypomania (code 9); MMPI_Pa, paranoia (code 6); MMPI_Pd, psychopathy (code 4); MMPI_Pt, psychasthenia (code 7); MMPI_Sc, schizophrenia (code 8); MMPI_Si, social introversion (code 0); NA, not available; NPV, negative predictive value; PPV, positive predictive value; RF, random forest; ROC, receiver operating characteristic; SRS, social responsiveness scale; SRS_Cog, social cognition; SRS_Com, social communication; SRS_Man, autistic mannerisms; SRS_Mot, social motivations; SVM, support vector machine classifier; XGBoost = eXtreme Gradient Boosting.

a The 95% confidence intervals for each estimate are presented in Supplementary material, pp. 27–50.

b Results of the model (RF or XGBoost model) with the higher ROC AUC on test set were presented.

Detailed results for each estimate on both training and test sets and the SHAP summary plots for RF and XGBoost are displayed in Supplementary material, pp. 27–50.

#### 3.3.2. Results of ADHD subgroups

For the sample with ADHD, the performance was only retained for total parenting stress: the RF model showed an AUC of 0.865 (95% CI 0.726–0.969), sensitivity of 0.882 (95% CI 0.706–1.000), and specificity of 0.706 (95% CI 0.474–0.917). Notably, ASD patients’ SRS-2 scores of social communication arose in the top 5 predictors of the RF model in this population. For the sample without ADHD, the performance was only retained for difficult child: the RF model showed an AUC of 0.854 (95% CI 0.755–0.933), sensitivity of 0.714 (95% CI 0.538–0.875), and specificity of 0.842 (95% CI 0.714–0.949) (Table 5 and Figure 3).

**Table 5:** Model performances on the test set with the top 10 major predictors in samples subgrouped by ADHD.^a^

		Parental distress				Parent-child dysfunctional interaction				Difficult child				Total parenting stress			
		RF model	XGBoost model	LR model	SVM model	RF model	XGBoost model	LR model	SVM model	RF model	XGBoost model	LR model	SVM model	RF model	XGBoost model	LR model	SVM model
Sample with ADHD (*n* = 168)	ROC AUC	0.794	0.818	0.739	0.771	0.884	0.874	0.839	0.877	0.709	0.678	0.657	0.311	0.865	0.834	0.886	0.886
	Sensitivity	0.455	0.455	0.364	0.545	0.667	0.467	0.600	0.533	0.706	0.765	0.588	0.588	0.882	0.824	0.882	1.000
	Specificity	1.000	1.000	0.957	0.870	0.842	0.895	0.895	1.000	0.588	0.647	0.529	0.529	0.706	0.706	0.529	0.000
	PPV	1.000	1.000	0.800	0.667	0.769	0.778	0.818	1.000	0.632	0.684	0.556	0.556	0.750	0.737	0.652	0.500
	NPV	0.793	0.793	0.759	0.800	0.762	0.680	0.739	0.731	0.667	0.733	0.562	0.562	0.857	0.800	0.818	NA
	Accuracy	0.824	0.824	0.765	0.765	0.765	0.706	0.765	0.794	0.647	0.706	0.559	0.559	0.794	0.765	0.706	0.500
	Top 10 predictors (ordered by importance)^b^	MMPI_D MMPI_Sc MMPI_Pa CBCL_Withdrawn MMPI_Pt				CBCL_Withdrawn CBCL_Att SRS_Mot MMPI_Si MMPI_D				CBCL_Agg SRS_Man CBCL_Anx/Dep MMPI_Pd CBCL_Other				CBCL_Withdrawn MMPI_Pa MMPI_D CBCL_Agg SRS_Com			
		SRS_Man SRS_Mot MMPI_Si SRS_Com SRS_Cog				MMPI_Pa SRS_Com CBCL_Agg SRS_Cog SRS_Man				MMPI_D SRS_Cog MMPI_Sc Age of caregiver MMPI_Si				MMPI_Pt MMPI_Sc MMPI_Si SRS_Mot MMPI_Hs			
Sample without ADHD (*n* = 328)	ROC AUC	0.781	0.765	0.828	0.843	0.734	0.718	0.762	0.745	0.854	0.827	0.831	0.836	0.759	0.755	0.755	0.753
	Sensitivity	0.389	0.222	0.333	0.500	0.630	0.667	0.667	0.000	0.714	0.679	0.679	0.000	0.742	0.742	0.710	0.677
	Specificity	0.938	0.958	1.000	0.938	0.718	0.744	0.744	1.000	0.842	0.789	0.816	1.000	0.629	0.657	0.657	0.657
	PPV	0.700	0.667	1.000	0.750	0.607	0.643	0.643	NA	0.769	0.704	0.731	NA	0.639	0.657	0.647	0.636
	NPV	0.804	0.767	0.800	0.833	0.737	0.763	0.763	0.591	0.800	0.769	0.775	0.576	0.733	0.742	0.719	0.697
	Accuracy	0.788	0.758	0.818	0.818	0.682	0.712	0.712	0.591	0.788	0.742	0.758	0.576	0.682	0.697	0.682	0.667
	Top 10 predictors (ordered by importance)^b^	MMPI_Sc MMPI_Si MMPI_D MMPI_Pd MMPI_Pa				SRS_Com CBCL_Withdrawn CBCL_Att SRS_Cog CBCL_Agg				CBCL_Agg CBCL_Other CBCL_Withdrawn MMPI_Pt MMPI_D				CBCL_Agg MMPI_D MMPI_Sc MMPI_Si CBCL_Other			
		CBCL_Agg SRS_Cog MMPI_Ma CBCL_Att FSIQ				CBCL_Other MMPI_D SRS_Man MMPI_Pt SRS_Mot				CBCL_Anx/Dep CBCL_Att MMPI_Si SRS_Cog MMPI_Pa				CBCL_Anx/Dep MMPI_Pt MMPI_Pd CBCL_Withdrawn CBCL_Att			

ADHD, attention-deficit/hyperactivity disorder; AUC, area under the curve; CBCL, Child Behavior Checklist; CBCL_Agg, aggressive behavior; CBCL_Anx/Dep, anxious/depressed; CBCL_Att, attention problems; CBCL_Other, other problems; CBCL_Withdrawn, withdrawn; FSIQ, full scale intelligence quotient; LR, logistic regression; MMPI, Minnesota Multiphasic Personality Inventory; MMPI_D, depression (code 2); MMPI_Hs, hypochondriasis (code 1); MMPI_Ma, hypomania (code 9); MMPI_Pa, paranoia (code 6); MMPI_Pd, psychopathy (code 4); MMPI_Pt, psychasthenia (code 7); MMPI_Sc, schizophrenia (code 8); MMPI_Si, social introversion (code 0); NA, not available; NPV, negative predictive value; PPV, positive predictive value; RF, random forest, ROC; receiver operating characteristic; SRS, social responsiveness scale; SRS_Cog, social cognition; SRS_Com, social communication; SRS_Man, autistic mannerisms; SRS_Mot, social motivations; SVM, support vector machine; XGBoost, eXtreme Gradient Boosting.

a The 95% confidence intervals for each estimate are presented in Supplementary material, pp. 51–74.

b Results of the model (RF or XGBoost model) with the higher ROC AUC on test set were presented.

Detailed results for each estimate on both training and test sets and the SHAP summary plots for RF and XGBoost are displayed in Supplementary material, pp. 51–74.

## 4. Discussion

We evaluated the ML models predicting severe parenting stress and its components (parental distress, parent-child dysfunctional interaction, and difficult child) in caregivers of ASD patients and investigated major predictors. Our key findings were that our ML models could predict severe parental distress, parent-child dysfunctional interaction, difficult child, and total parenting stress with AUC values greater than 0.80. Moreover, we also identified major predictors for each outcome of interest by utilizing explainable ML models, which provided valuable insights into the underlying factors contributing to severe parenting stress in caregivers of ASD patients.

Parental distress measures a parent’s experiences of their role as parents (Abidin, 1990). Among the top 10 predictors for parental distress, seven were associated with the personality traits of caregivers [depression (code 2), schizophrenia (code 8), psychopathy (code 4), psychasthenia (code 7), paranoia (code 6), social introversion (code 0), and hypomania (code 9)], which means that caregivers’ perceived hardship related to the role as parents may be determined primarily by their psychopathology. However, an observational study using regression analysis reported that ASD patients’ emotional problems (regression coefficient = 0.31) may also play a significant role in parental distress (Mello et al., 2022). Since our main ML models using the overall sample only included common subscales of CBCL between 1.5–5 and 6–18, and thereby scores of emotional problems were excluded, the potential impact of patients’ behavioral problems on caregivers’ parental distress should not be ignored. Indeed, our subgroup analysis that used CBCL 6–18 sample showed that the patients’ behavioral problems were also essential predictors in predicting parental distress in caregivers of ASD patients. Nevertheless, when considering that most previous studies only utilized ASD patient factors as associated/predictive factors for parental distress (Scibelli et al., 2021; Mello et al., 2022), our study provided a new insight into the understanding of parental distress by utilizing explainable ML models with model features related to caregivers’ psychopathology. However, interpretation needs caution since MMPI-2 clinical scales should not be independently addressed (Levak et al., 2012). A comprehensive approach for significant MMPI-2 clinical scales might be appropriate. For example, we may expect that a caregiver of 2-4-8 code type (the top 3 predictors for parental distress) would experience substantial difficulties in their role as parents as to the vulnerability to substance abuse, poor impulse control, emotional dysregulation, posttraumatic stress disorder symptoms, thought disorder, and borderline personality disorder, which might represent moderate to severe psychopathology and require major psychiatric interventions (Archer et al., 1995; Bell-Pringle et al., 1997; Donovan et al., 1998).

Parent-child dysfunctional interaction measures parents’ feelings about the interaction with their child with ASD (Abidin, 1990). Interestingly, our findings implied that behavioral problems of children (withdrawn, aggressive behavior, and attention problems) contributed more to caregivers’ negative feelings on their interaction with the children compared to ASD core symptoms, even though the latter ones seemed to be more directly associated with their interaction. Furthermore, a cross-sectional study observed that the correlation coefficient (*r*) between total ASD core symptoms and parent-child dysfunctional interaction (0.305) was attenuated when considering its individual subscales. Specifically, the correlations with parent-child dysfunctional interaction were estimated to be 0.184 for reciprocal social interaction, 0.212 for social communication, and 0.288 for repetitive and restricted behaviors (Scibelli et al., 2021). However, a separate study that employed a regression analysis highlighted the significance of ASD core symptoms on parent-child dysfunctional interaction (ASD core symptom severity: regression coefficient = 0.48, *p* < 0.001) (Mello et al., 2022). This indicates that the impact of ASD core symptoms should not be underestimated and remains a significant concern. In fact, two of the top 5 predictors were related to ASD core symptoms (social communication and cognition) in the subgroup analysis using the CBCL 1.5–5 sample. Taken together, our results suggested that both ASD core symptoms and behavioral problems of ASD patients are important in predicting parent-child dysfunctional interaction.

Difficult child measures a parent’s perception of whether the child is easy or difficult to nurture (Abidin, 1990). Our models found that ASD patients’ behavioral problems (aggressive behavior, anxious/depressed, other problems, attention problems, and withdrawn), caregivers’ psychopathology [social introversion (code 0) and depression (code 2)], and ASD patients’ core symptoms (autistic mannerisms and social cognition) mainly contributed to the caregivers’ perception that their child was difficult to care. These results are consistent with the previous studies that have reported associated/predictive factors for difficult child in parents of ASD patients. Scibelli et al. found that difficult child (as measured by PSI-SF) was significantly correlated with ASD core symptoms (total symptoms: *r* = 0.409; repetitive and restricted behavior: *r* = 0.409) and behavioral problems (social problems: *r* = 0.485; thought problems: *r* = 0.671; attention problems: *r* = 0.502; rule-breaking behavior: *r* = 0.498; aggressive behavior: *r* = 0.555) in ASD without cognitive impairment (Scibelli et al., 2021). Mello et al. (2022) have also reported significant impacts of ASD core symptoms (regression coefficient = 0.46) and aggressive behavior (regression coefficient = 0.36) on difficult child. Together with our findings, this suggested that the severity of core symptoms and behavioral problems seemed to directly contribute to the difficulties faced by caregivers in their upbringing responsibilities. However, our study was the first to report that caregivers’ personality traits were also major predictors for difficult child, even outranking ASD core symptoms. That is, the caregivers’ personality traits also significantly affect how they perceive difficulty in raising their child. When considering a caregiver of the 2-0 code type, for example, one may be challenged by their child’s aspects that make one feels tough to raise, such as aggressive behavior, because these people tend to represent chronic depression, guilty, social withdrawal, and lack of confidence (Levak et al., 2012). Additionally, higher scale scores of 2-0 code type are clinically associated with unipolar depression or remitted depression, and pessimism, the negative cognition of which might lead to a bias that affects measuring a child as a more difficult child (Wetzler et al., 1995; Suzuki et al., 2014).

Total parenting stress measures the overall stress level in the role of parents (Abidin, 1990). The main contributors to the overall stress of caregivers were the ASD patients’ behavioral problems (aggressive behavior, anxious/depressed, attention problems, withdrawn, and other problems) and caregivers’ psychopathology [depression (code 2), social introversion (code 0), psychasthenia (code 7), and schizophrenia (code 8)], whereas ASD core symptoms showed less predictive power. This suggested that individualized interventions for caregivers targeting their mental health in the context of their psychopathologic profile might be helpful in alleviating parenting stress and subsequently improving treatment outcomes for ASD patients (Osborne et al., 2008). Indeed, the current interventions of mainstream generally aimed at maximizing ASD patients’ functioning by improving their core symptoms or specific behavioral problems (Lai et al., 2014). Psychological interventions targeting parenting stress were also suggested, of which the efficacy was confirmed by a meta-analysis of 16 randomized controlled trials with moderate certainty of evidence (standardized mean difference −0.33, 95% CI −0.46 to −0.19) (Kulasinghe et al., 2022). However, none of the meta-analyzed trials have included caregivers’ psychopathology as a treatment target. Our findings that caregivers’ psychopathology was also a reliable predictor of total parenting stress suggested that interventions may benefit from including caregivers’ psychopathology as a novel therapeutic target.

Meanwhile, there is a tendency that in the CBCL 1.5–5 sample, model features associated with the caregivers’ psychopathology were ranked higher than behavioral problems; however, this was inverted in the CBCL 6–18 sample. This might result from the tendency that the severity of behavioral problems tended to be higher in the CBCL 6–18 sample than in CBCL 1.5–5. For subgroup analysis by comorbid ADHD, the model features related to ASD core symptoms rose in rank in the sample with ADHD compared to the main analysis. This may indicate that addictive deficits in the social domain of both ASD and ADHD have more contributed to parenting stress than the ousted predictors (Mikami et al., 2019).

This study has some limitations. First, the performance of ML models was not confirmed by the external validation set. Second, given that generated models did not show the perfect performance, failing to achieve an AUC larger than 0.90, it could be hypothesized that some potential predictors of parenting stress, such as family income, may have been missed. Third, the thorough interpretation of the MMPI was not possible because the MMPI should be addressed in the context of validity scales, but only clinical scales were employed as model features. Fourth, the SRS-2 and CBCL were rated by a caregiver, possibly leading to the overestimation of the ASD patients’ status, especially in those with high parenting stress (Schwartzman et al., 2021). Lastly, the study period was insufficient to investigate the impact of the COVID-19 pandemic on the parenting stress of this population, which calls for further studies.

In conclusion, we identified major predictors for each component of parenting stress in ASD patients’ primary caregivers using explainable ML models. This study revealed specific components of caregivers’ psychopathology, ASD patients’ core symptoms, and behavioral problems which mainly contribute to parenting stress. Our ML models and the identified predictors would be helpful in alerting physicians whether a caregiver is at a high risk of experiencing severe parenting stress and if so, providing timely interventions, which could eventually improve the treatment outcome for ASD patients.

## Data availability statement

The raw data supporting the conclusions of this article will be made available by the authors, without undue reservation.

## Ethics statement

The studies involving humans were approved by Institutional Review Board of the Severance Hospital of Yonsei University, Seoul, the Republic of Korea. The studies were conducted in accordance with the local legislation and institutional requirements. The ethics committee/institutional review board waived the requirement of written informed consent for participation from the participants or the participants’ legal guardians/next of kin because informed consent was waived since the authors used retrospective and deidentified patient data.

## Author contributions

HC, JK, HK, and K-AC conceived and designed the study. HC managed data collection and checked data eligibility. JK performed the machine learning-based analysis. HC and HK inspected the completion of the analysis. HC and JK drafted the manuscript. HK and K-AC provided critical revision of the drafted manuscript. All authors contributed to the article and approved the submitted version.

## Conflict of interest

The authors declare that the research was conducted in the absence of any commercial or financial relationships that could be construed as a potential conflict of interest.

## Publisher’s note

All claims expressed in this article are solely those of the authors and do not necessarily represent those of their affiliated organizations, or those of the publisher, the editors and the reviewers. Any product that may be evaluated in this article, or claim that may be made by its manufacturer, is not guaranteed or endorsed by the publisher.

## References

[ref1] Abidin R. R. (1990). Parenting stress index-short form. Pediatric Psychology Press, Charlottesville, VA.

[ref2] Achenbach T. M. (1999). The Child Behavior Checklist and related instruments, The use of psychological testing for treatment planning and outcomes assessment, 2 Mahwah, NJ: Lawrence Erlbaum Associates Publishers, 429–466.

[ref3] Archer R. P. Griffin R. Aiduk R. (1995). MMPI-2 clinical correlates for ten common codes. J. Pers. Assess. 65, 391–407. doi: 10.1207/s15327752jpa6503_1, PMID: 16367707

[ref4] American Psychiatric Association (2013). Diagnostic and statistical manual of mental disorders: DSM-5. Arlington, VA: American Psychiatric Association.

[ref5] Bell-Pringle V. J. Pate J. L. Brown R. C. (1997). Assessment of borderline personality disorder using the MMPI-2 and the personality assessment inventory. Assessment 4, 131–139. doi: 10.1177/107319119700400203

[ref6] Bergstra J. Bengio Y. (2012). Random search for hyper-parameter optimization. J. Mach. Learn. Res. 13, 281–305.

[ref7] Chun J. Bong G. Han J. H. Oh M. Yoo H. J. (2021). Validation of social responsiveness scale for Korean preschool children with autism. Psychiatry Investig. 18, 831–840. doi: 10.30773/pi.2021.0182, PMID: 34500507 PMC8473854

[ref8] Constantino J. N. (2012). Social responsiveness scale (SRS-2). 2nd Edn. Los Angeles: Western Psychological Services.

[ref9] Donovan J. M. Soldz S. Kelley H. F. Penk W. E. (1998). Four addictions: the MMPI and discriminant function analysis. J. Addict. Dis. 17, 41–55. doi: 10.1300/J069v17n02_04 9567225

[ref10] Falk N. H. Norris K. Quinn M. G. (2014). The factors predicting stress, anxiety and depression in the parents of children with autism. J. Autism Dev. Disord. 44, 3185–3203. doi: 10.1007/s10803-014-2189-4, PMID: 25022253

[ref11] Graham J. R. (1990). MMPI-2: assessing personality and psychopathology. New York: Oxford University Press.

[ref12] Han K. Lim J. Kim Z. Min B. Lee J. Moon K. (2006). Korean MMPI-2 standardization study. Korean J. Clin. Psychol. 25, 533–564.

[ref13] Han M. H. Yoo A. J. (1995). The validation of the Child Behavior Checklist. Korean J Child Stud 16, 5–21.

[ref14] Hayes S. A. Watson S. L. (2013). The impact of parenting stress: a meta-analysis of studies comparing the experience of parenting stress in parents of children with and without autism spectrum disorder. J. Autism Dev. Disord. 43, 629–642. doi: 10.1007/s10803-012-1604-y, PMID: 22790429

[ref15] Kulasinghe K. Whittingham K. Mitchell A. E. Boyd R. N. (2022). Psychological interventions targeting mental health and the mother-child relationship in autism: systematic review and meta-analysis. Dev. Med. Child Neurol. 65, 329–345. doi: 10.1111/dmcn.15432, PMID: 36208472 PMC10953452

[ref16] Lai M. C. Lombardo M. V. Baron-Cohen S. (2014). Autism. Lancet 383, 896–910. doi: 10.1016/s0140-6736(13)61539-1 24074734

[ref17] Lee K.-S. Chung K. Park J. Kim H.-J. (2008). Reliability and validity study for the Korean version of parenting stress index short form (K-PSI-SF). Korean J. Woman Psychol. 13, 363–377. doi: 10.18205/kpa.2008.13.3.007

[ref18] Leonardi E. Cerasa A. Servidio R. Costabile A. Famà F. I. Carrozza C.. (2021). The route of stress in parents of young children with and without autism: a path-analysis study. Int. J. Environ. Res. Public Health 18:10887. doi: 10.3390/ijerph182010887, PMID: 34682634 PMC8535200

[ref19] Levak R. W. Siegel L. Nichols D. S. (2012). Therapeutic feedback with the MMPI-2: a positive psychology approach. Routledge/Taylor & Francis Group..

[ref20] Lundberg S. M. Erion G. Chen H. DeGrave A. Prutkin J. M. Nair B.. (2020). From local explanations to global understanding with explainable AI for trees. Nat. Mach. Intell. 2, 56–67. doi: 10.1038/s42256-019-0138-9, PMID: 32607472 PMC7326367

[ref21] Mello C. Rivard M. Morin D. Patel S. Morin M. (2022). Symptom severity, internalized and externalized behavioral and emotional problems: links with parenting stress in mothers of children recently diagnosed with autism. J. Autism Dev. Disord. 52, 2400–2413. doi: 10.1007/s10803-021-05131-4, PMID: 34120257

[ref22] Mikami A. Y. Miller M. Lerner M. D. (2019). Social functioning in youth with attention-deficit/hyperactivity disorder and autism spectrum disorder: transdiagnostic commonalities and differences. Clin. Psychol. Rev. 68, 54–70. doi: 10.1016/j.cpr.2018.12.005 30658861

[ref23] Miranda A. Mira A. Berenguer C. Rosello B. Baixauli I. (2019). Parenting stress in mothers of children with autism without intellectual disability. Mediation of behavioral problems and coping strategies. Front. Psychol. 10:464. doi: 10.3389/fpsyg.2019.00464, PMID: 30906274 PMC6418028

[ref24] Neter J. Kutner M. H. Nachtsheim C. J. Wasserman W. (1996). Applied linear statistical models. New York: WCB McGraw-Hill.

[ref25] Osborne L. A. McHugh L. Saunders J. Reed P. (2008). Parenting stress reduces the effectiveness of early teaching interventions for autistic spectrum disorders. J. Autism Dev. Disord. 38, 1092–1103. doi: 10.1007/s10803-007-0497-7, PMID: 18027079

[ref26] Schwalbe N. Wahl B. (2020). Artificial intelligence and the future of global health. Lancet 395, 1579–1586. doi: 10.1016/s0140-6736(20)30226-9, PMID: 32416782 PMC7255280

[ref27] Schwartzman J. M. Hardan A. Y. Gengoux G. W. (2021). Parenting stress in autism spectrum disorder may account for discrepancies in parent and clinician ratings of child functioning. Autism 25, 1601–1614. doi: 10.1177/1362361321998560, PMID: 33691519

[ref28] Scibelli F. Fucà E. Guerrera S. Lupi E. Alfieri P. Valeri G.. (2021). Clinical and individual features associated with maternal stress in young adolescents with autism spectrum disorder. Autism Res. 14, 1935–1947. doi: 10.1002/aur.2539, PMID: 34013607

[ref29] Siu Q. K. Y. Yi H. Chan R. C. H. Chio F. H. N. Chan D. F. Y. Mak W. W. S. (2019). The role of child problem behaviors in autism spectrum symptoms and parenting stress: a primary school-based study. J. Autism Dev. Disord. 49, 857–870. doi: 10.1007/s10803-018-3791-7, PMID: 30367345

[ref30] Suzuki M. Takahashi M. Muneoka K. Sato K. Hashimoto K. Shirayama Y. (2014). A study of remitted and treatment-resistant depression using MMPI and including pessimism and optimism scales. PLoS One 9:e109137. doi: 10.1371/journal.pone.0109137, PMID: 25279466 PMC4184846

[ref31] Voliovitch Y. Leventhal J. M. Fenick A. M. Gupta A. R. Feinberg E. Hickey E. J.. (2021). Parenting stress and its associated components prior to an autism spectrum disorder (ASD) diagnostic evaluation. J. Autism Dev. Disord. 51, 3432–3442. doi: 10.1007/s10803-020-04804-w 33387245

[ref32] von Elm E. Altman D. G. Egger M. Pocock S. J. Gøtzsche P. C. Vandenbroucke J. P. (2007). Strengthening the reporting of observational studies in epidemiology (STROBE) statement: guidelines for reporting observational studies. BMJ 335, 806–808. doi: 10.1136/bmj.39335.541782.AD 17947786 PMC2034723

[ref33] Wetzler S. Khadivi A. Oppenheim S. (1995). The psychological assessment of depression: unipolars versus bipolars. J. Pers. Assess. 65, 557–566. doi: 10.1207/s15327752jpa6503_14, PMID: 8609588

[ref34] Yorke I. White P. Weston A. Rafla M. Charman T. Simonoff E. (2018). The association between emotional and behavioral problems in children with autism spectrum disorder and psychological distress in their parents: a systematic review and meta-analysis. J. Autism Dev. Disord. 48, 3393–3415. doi: 10.1007/s10803-018-3605-y, PMID: 29777471 PMC6153902

