# Supplementary material for: Identifying major predictors for parenting stress in a caregiver of autism spectrum disorder using machine learning models
Source: Front Neurosci. 2023 Aug 29;17:1229155. doi: 10.3389/fnins.2023.1229155 (PMC10495987; doi:10.3389/fnins.2023.1229155)
Supplement: Supplementary file 1 [file Data_Sheet_1.DOCX]

**Supplementary materials**

**Identifying major predictors for parenting stress in a caregiver of autism spectrum disorder using machine learning models**

Table of Contents

[eAppendix 1. STROBE Statement—checklist of items that should be included in reports of observational studies 6](#_Toc142240329)

[eAppendix 2. Definitions and measurements for model features 8](#_Toc142240330)

[eTable 2.1. Caregiver variables 8](#_Toc142240331)

[eTable 2.2. Autism spectrum disorder (ASD) patient variables 9](#_Toc142240332)

[eAppendix 3. Missing value information 11](#_Toc142240333)

[eAppendix 4. Formulas for variance inflation factor, area under the receiver operating curve, sensitivity, specificity, positive predictive value, negative predictive value, and accuracy 13](#_Toc142240334)

[eAppendix 5. Variance inflation factor calculation for continuous features 14](#_Toc142240335)

[eAppendix 6. Model information and detailed results (Sample: Overall sample [n=496], Outcome: Parental distress) 15](#_Toc142240336)

[eTable 6.1. Model features (Variables are on the second columns) 15](#_Toc142240337)

[eTable 6.2. Performance of each model on the training set 16](#_Toc142240338)

[eTable 6.3. Performance of each model on the test set 16](#_Toc142240339)

[eFigure 6.4. SHapley Additive exPlanations (SHAP) summary plot for RF and XGBoost models (only top 20 predictors were presented) 17](#_Toc142240340)

[eFigure 6.5. Receiver operating characteristic curves (ROC) for the training and test set 17](#_Toc142240341)

[eAppendix 7. Model information and detailed results (Sample: Overall sample [n=496] Outcome: Parent-child dysfunctional interaction) 18](#_Toc142240342)

[eTable 7.1. Model features (Variables are on the second columns) 18](#_Toc142240343)

[eTable 7.2. Performance of each model on the training set 19](#_Toc142240344)

[eTable 7.3. Performance of each model on the test set 19](#_Toc142240345)

[eFigure 7.4. SHapley Additive exPlanations (SHAP) summary plot for RF and XGBoost models (only top 20 predictors were presented) 20](#_Toc142240346)

[eFigure 7.5. Receiver operating characteristic curves (ROC) for the training and test set 20](#_Toc142240347)

[eAppendix 8. Model information and detailed results (Sample: Overall sample [n=496], Outcome: Difficult child) 21](#_Toc142240348)

[eTable 8.1. Model features (Variables are on the second columns) 21](#_Toc142240349)

[eTable 8.2. Performance of each model on the training set 22](#_Toc142240350)

[eTable 8.3. Performance of each model on the test set 22](#_Toc142240351)

[eFigure 8.4. SHapley Additive exPlanations (SHAP) summary plot for RF and XGBoost models (only top 20 predictors were presented) 23](#_Toc142240352)

[eFigure 8.5. Receiver operating characteristic curves (ROC) for the training and test set 23](#_Toc142240353)

[eAppendix 9. Model information and detailed results (Sample: Overall sample [n=496], Outcome: Total parenting stress) 24](#_Toc142240354)

[eTable 9.1. Model features (Variables are on the second columns) 24](#_Toc142240355)

[eTable 9.2. Performance of each model on the training set 25](#_Toc142240356)

[eTable 9.3. Performance of each model on the test set 25](#_Toc142240357)

[eFigure 9.4. SHapley Additive exPlanations (SHAP) summary plot for RF and XGBoost models (only top 20 predictors were presented) 26](#_Toc142240358)

[eFigure 9.5. Receiver operating characteristic curves (ROC) for the training and test set 26](#_Toc142240359)

[eAppendix 10. Model information and detailed results (Sample: CBCL 1.5-5 sample [n=194], Outcome: Parental distress) 27](#_Toc142240360)

[eTable 10.1. Model features (Variables are on the second columns) 27](#_Toc142240361)

[eTable 10.2. Performance of each model on the training set 28](#_Toc142240362)

[eTable 10.3. Performance of each model on the test set 28](#_Toc142240363)

[eFigure 10.4. SHapley Additive exPlanations (SHAP) summary plot for RF and XGBoost models (only top 20 predictors were presented) 29](#_Toc142240364)

[eFigure 10.5. Receiver operating characteristic curves (ROC) for the training and test set 29](#_Toc142240365)

[eAppendix 11. Model information and detailed results (Sample: CBCL 1.5-5 sample [n=194], Outcome: Parent-child dysfunctional interaction) 30](#_Toc142240366)

[eTable 11.1. Model features (Variables are on the second columns) 30](#_Toc142240367)

[eTable 11.2. Performance of each model on the training set 31](#_Toc142240368)

[eTable 11.3. Performance of each model on the test set 31](#_Toc142240369)

[eFigure 11.4. SHapley Additive exPlanations (SHAP) summary plot for RF and XGBoost models (only top 20 predictors were presented) 32](#_Toc142240370)

[eFigure 11.5. Receiver operating characteristic curves (ROC) for the training and test set 32](#_Toc142240371)

[eAppendix 12. Model information and detailed results (Sample: CBCL 1.5-5 sample [n=194], Outcome: Difficult child) 33](#_Toc142240372)

[eTable 12.1. Model features (Variables are on the second columns) 33](#_Toc142240373)

[eTable 12.2. Performance of each model on the training set 34](#_Toc142240374)

[eTable 12.3. Performance of each model on the test set 34](#_Toc142240375)

[eFigure 12.4. SHapley Additive exPlanations (SHAP) summary plot for RF and XGBoost models (only top 20 predictors were presented) 35](#_Toc142240376)

[eFigure 12.5. Receiver operating characteristic curves (ROC) for the training and test set 35](#_Toc142240377)

[eAppendix 13. Model information and detailed results (Sample: CBCL 1.5-5 sample [n=194], Outcome: Total parenting stress) 36](#_Toc142240378)

[eTable 13.1. Model features (Variables are on the second columns) 36](#_Toc142240379)

[eTable 13.2. Performance of each model on the training set 37](#_Toc142240380)

[eTable 13.3. Performance of each model on the test set 37](#_Toc142240381)

[eFigure 13.4. SHapley Additive exPlanations (SHAP) summary plot for RF and XGBoost models (only top 20 predictors were presented) 38](#_Toc142240382)

[eFigure 13.5. Receiver operating characteristic curves (ROC) for the training and test set 38](#_Toc142240383)

[eAppendix 14. Model information and detailed results (Sample: CBCL 6-18 sample [n=302], Outcome: Parental distress) 39](#_Toc142240384)

[eTable 14.1. Model features (Variables are on the second columns) 39](#_Toc142240385)

[eTable 14.2. Performance of each model on the training set 40](#_Toc142240386)

[eTable 14.3. Performance of each model on the test set 40](#_Toc142240387)

[eFigure 14.4. SHapley Additive exPlanations (SHAP) summary plot for RF and XGBoost models (only top 20 predictors were presented) 41](#_Toc142240388)

[eFigure 14.5. Receiver operating characteristic curves (ROC) for the training and test set 41](#_Toc142240389)

[eAppendix 15. Model information and detailed results (Sample: CBCL 6-18 sample [n=302], Outcome: Parent-child dysfunctional interaction) 42](#_Toc142240390)

[eTable 15.1. Model features (Variables are on the second columns) 42](#_Toc142240391)

[eTable 15.2. Performance of each model on the training set 43](#_Toc142240392)

[eTable 15.3. Performance of each model on the test set 43](#_Toc142240393)

[eFigure 15.4. SHapley Additive exPlanations (SHAP) summary plot for RF and XGBoost models (only top 20 predictors were presented) 44](#_Toc142240394)

[eFigure 15.5. Receiver operating characteristic curves (ROC) for the training and test set 44](#_Toc142240395)

[eAppendix 16. Model information and detailed results (Sample: CBCL 6-18 sample [n=302], Outcome: Difficult child) 45](#_Toc142240396)

[eTable 16.1. Model features (Variables are on the second columns) 45](#_Toc142240397)

[eTable 16.2. Performance of each model on the training set 46](#_Toc142240398)

[eTable 16.3. Performance of each model on the test set 46](#_Toc142240399)

[eFigure 16.4. SHapley Additive exPlanations (SHAP) summary plot for RF and XGBoost models (only top 20 predictors were presented) 47](#_Toc142240400)

[eFigure 16.5. Receiver operating characteristic curves (ROC) for the training and test set 47](#_Toc142240401)

[eAppendix 17. Model information and detailed results (Sample: CBCL 6-18 sample [n=302], Outcome: Total parenting stress) 48](#_Toc142240402)

[eTable 17.1. Model features (Variables are on the second columns) 48](#_Toc142240403)

[eTable 17.2. Performance of each model on the training set 49](#_Toc142240404)

[eTable 17.3. Performance of each model on the test set 49](#_Toc142240405)

[eFigure 17.4. SHapley Additive exPlanations (SHAP) summary plot for RF and XGBoost models (only top 20 predictors were presented) 50](#_Toc142240406)

[eFigure 17.5. Receiver operating characteristic curves (ROC) for the training and test set 50](#_Toc142240407)

[eAppendix 18. Model information and detailed results (Sample: with ADHD [n=168], Outcome: Parental distress) 51](#_Toc142240408)

[eTable 18.1. Model features (Variables are on the second columns) 51](#_Toc142240409)

[eTable 18.2. Performance of each model on the training set 52](#_Toc142240410)

[eTable 18.3. Performance of each model on the test set 52](#_Toc142240411)

[eFigure 18.4. SHapley Additive exPlanations (SHAP) summary plot for RF and XGBoost models (only top 20 predictors were presented) 53](#_Toc142240412)

[eFigure 18.5. Receiver operating characteristic curves (ROC) for the training and test set 53](#_Toc142240413)

[eAppendix 19. Model information and detailed results (Sample: with ADHD [n=168], Outcome: Parent-child dysfunctional interaction) 54](#_Toc142240414)

[eTable 19.1. Model features (Variables are on the second columns) 54](#_Toc142240415)

[eTable 19.2. Performance of each model on the training set 55](#_Toc142240416)

[eTable 19.3. Performance of each model on the test set 55](#_Toc142240417)

[eFigure 19.4. SHapley Additive exPlanations (SHAP) summary plot for RF and XGBoost models (only top 20 predictors were presented) 56](#_Toc142240418)

[eFigure 19.5. Receiver operating characteristic curves (ROC) for the training and test set 56](#_Toc142240419)

[eAppendix 20. Model information and detailed results (Sample: with ADHD [n=168], Outcome: Difficult child) 57](#_Toc142240420)

[eTable 20.1. Model features (Variables are on the second columns) 57](#_Toc142240421)

[eTable 20.2. Performance of each model on the training set 58](#_Toc142240422)

[eTable 20.3. Performance of each model on the test set 58](#_Toc142240423)

[eFigure 20.4. SHapley Additive exPlanations (SHAP) summary plot for RF and XGBoost models (only top 20 predictors were presented) 59](#_Toc142240424)

[eFigure 20.5. Receiver operating characteristic curves (ROC) for the training and test set 59](#_Toc142240425)

[eAppendix 21. Model information and detailed results (Sample: with ADHD [n=168], Outcome: Total parenting stress) 60](#_Toc142240426)

[eTable 21.1. Model features (Variables are on the second columns) 60](#_Toc142240427)

[eTable 21.2. Performance of each model on the training set 61](#_Toc142240428)

[eTable 21.3. Performance of each model on the test set 61](#_Toc142240429)

[eFigure 21.4. SHapley Additive exPlanations (SHAP) summary plot for RF and XGBoost models (only top 20 predictors were presented) 62](#_Toc142240430)

[eFigure 21.5. Receiver operating characteristic curves (ROC) for the training and test set 62](#_Toc142240431)

[eAppendix 22. Model information and detailed results (Sample: without ADHD [n=328], Outcome: Parental distress) 63](#_Toc142240432)

[eTable 22.1. Model features (Variables are on the second columns) 63](#_Toc142240433)

[eTable 22.2. Performance of each model on the training set 64](#_Toc142240434)

[eTable 22.3. Performance of each model on the test set 64](#_Toc142240435)

[eFigure 22.4. SHapley Additive exPlanations (SHAP) summary plot for RF and XGBoost models (only top 20 predictors were presented) 65](#_Toc142240436)

[eFigure 22.5. Receiver operating characteristic curves (ROC) for the training and test set 65](#_Toc142240437)

[eAppendix 23. Model information and detailed results (Sample: without ADHD [n=328], Outcome: Parent-child dysfunctional interaction) 66](#_Toc142240438)

[eTable 23.1. Model features (Variables are on the second columns) 66](#_Toc142240439)

[eTable 23.2. Performance of each model on the training set 67](#_Toc142240440)

[eTable 23.3. Performance of each model on the test set 67](#_Toc142240441)

[eFigure 23.4. SHapley Additive exPlanations (SHAP) summary plot for RF and XGBoost models (only top 20 predictors were presented) 68](#_Toc142240442)

[eFigure 23.5. Receiver operating characteristic curves (ROC) for the training and test set 68](#_Toc142240443)

[eAppendix 24. Model information and detailed results (Sample: without ADHD [n=328], Outcome: Difficult child) 69](#_Toc142240444)

[eTable 24.1. Model features (Variables are on the second columns) 69](#_Toc142240445)

[eTable 24.2. Performance of each model on the training set 70](#_Toc142240446)

[eTable 24.3. Performance of each model on the test set 70](#_Toc142240447)

[eFigure 24.4. SHapley Additive exPlanations (SHAP) summary plot for RF and XGBoost models (only top 20 predictors were presented) 71](#_Toc142240448)

[eFigure 24.5. Receiver operating characteristic curves (ROC) for the training and test set 71](#_Toc142240449)

[eAppendix 25. Model information and detailed results (Sample: without ADHD [n=328], Outcome: Total parenting stress) 72](#_Toc142240450)

[eTable 25.1. Model features (Variables are on the second columns) 72](#_Toc142240451)

[eTable 25.2. Performance of each model on the training set 73](#_Toc142240452)

[eTable 25.3. Performance of each model on the test set 73](#_Toc142240453)

[eFigure 25.4. SHapley Additive exPlanations (SHAP) summary plot for (only top 20 predictors were presented) 74](#_Toc142240454)

[eFigure 25.5. Receiver operating characteristic curves (ROC) for the training and test set 74](#_Toc142240455)

# **eAppendix 1. STROBE Statement—checklist of items that should be included in reports of observational studies**

|  | **Item No.** | **Recommendation** | **Page  No.** |
| --- | --- | --- | --- |
| **Title and abstract** | 1 | (*a*) Indicate the study’s design with a commonly used term in the title or the abstract | Title page |
|  |  | (*b*) Provide in the abstract an informative and balanced summary of what was done and what was found | Abstract |
| **Introduction** | | | |
| Background/rationale | 2 | Explain the scientific background and rationale for the investigation being reported | Manuscript #3 |
| Objectives | 3 | State specific objectives, including any prespecified hypotheses | Manuscript #3 |
| **Methods** | | | |
| Study design | 4 | Present key elements of study design early in the paper | Manuscript #3-4 |
| Setting | 5 | Describe the setting, locations, and relevant dates, including periods of recruitment, exposure, follow-up, and data collection | Manuscript #3-4 |
| Participants | 6 | (*a*) *Cohort study*—Give the eligibility criteria, and the sources and methods of selection of participants. Describe methods of follow-up  *Case-control study*—Give the eligibility criteria, and the sources and methods of case ascertainment and control selection. Give the rationale for the choice of cases and controls  *Cross-sectional study*—Give the eligibility criteria, and the sources and methods of selection of participants | Manuscript #3-4 |
|  |  | (*b*) *Cohort study*—For matched studies, give matching criteria and number of exposed and unexposed  *Case-control study*—For matched studies, give matching criteria and the number of controls per case | Not applicable |
| Variables | 7 | Clearly define all outcomes, exposures, predictors, potential confounders, and effect modifiers. Give diagnostic criteria, if applicable | Manuscript #4 |
| Data sources/ measurement | 8* | For each variable of interest, give sources of data and details of methods of assessment (measurement). Describe comparability of assessment methods if there is more than one group | Appendix #8-10 |
| Bias | 9 | Describe any efforts to address potential sources of bias | Not applicable |
| Study size | 10 | Explain how the study size was arrived at | Not applicable |
| Quantitative variables | 11 | Explain how quantitative variables were handled in the analyses. If applicable, describe which groupings were chosen and why | Manuscript #4 |
| Statistical methods | 12 | (*a*) Describe all statistical methods, including those used to control for confounding | Manuscript #5 |
|  |  | (*b*) Describe any methods used to examine subgroups and interactions | Manuscript #5 |
|  |  | (*c*) Explain how missing data were addressed | Manuscript #4 |
|  |  | (*d*) *Cohort study*—If applicable, explain how loss to follow-up was addressed  *Case-control study*—If applicable, explain how matching of cases and controls was addressed  *Cross-sectional study*—If applicable, describe analytical methods taking account of sampling strategy | Not applicable |
|  |  | (*e*) Describe any sensitivity analyses | Manuscript #5 |
| Participants | 13* | (a) Report numbers of individuals at each stage of study—eg numbers potentially eligible, examined for eligibility, confirmed eligible, included in the study, completing follow-up, and analysed | Manuscript #5-6 |
|  |  | (b) Give reasons for non-participation at each stage | Not applicable |
|  |  | (c) Consider use of a flow diagram | Not applicable |
| Descriptive data | 14* | (a) Give characteristics of study participants (eg demographic, clinical, social) and information on exposures and potential confounders | Table 1 |
|  |  | (b) Indicate number of participants with missing data for each variable of interest | Appendix #11-12 |
|  |  | (c) *Cohort study*—Summarise follow-up time (eg, average and total amount) | Not applicable |
| Outcome data | 15* | *Cohort study*—Report numbers of outcome events or summary measures over time | *-* |
|  |  | *Case-control study—*Report numbers in each exposure category, or summary measures of exposure | *-* |
|  |  | *Cross-sectional study—*Report numbers of outcome events or summary measures | Manuscript #5-6 |
| Main results | 16 | (*a*) Give unadjusted estimates and, if applicable, confounder-adjusted estimates and their precision (eg, 95% confidence interval). Make clear which confounders were adjusted for and why they were included | Manuscript #5-6 |
|  |  | (*b*) Report category boundaries when continuous variables were categorized | Not applicable |
|  |  | (*c*) If relevant, consider translating estimates of relative risk into absolute risk for a meaningful time period | Not applicable |
| Other analyses | 17 | Report other analyses done—eg analyses of subgroups and interactions, and sensitivity analyses | Manuscript #6 |
| **Discussion** | | | |
| Key results | 18 | Summarise key results with reference to study objectives | Manuscript #7 |
| Limitations | 19 | Discuss limitations of the study, taking into account sources of potential bias or imprecision. Discuss both direction and magnitude of any potential bias | Manuscript #8-9 |
| Interpretation | 20 | Give a cautious overall interpretation of results considering objectives, limitations, multiplicity of analyses, results from similar studies, and other relevant evidence | Manuscript #7-8 |
| Generalisability | 21 | Discuss the generalisability (external validity) of the study results | Manuscript #7-8 |
| **Other information** | | | |
| Funding | 22 | Give the source of funding and the role of the funders for the present study and, if applicable, for the original study on which the present article is based | Acknowledgement |

*Give information separately for cases and controls in case-control studies and, if applicable, for exposed and unexposed groups in cohort and cross-sectional studies.

von Elm E, Altman DG, Egger M, Pocock SJ, Gøtzsche PC, Vandenbroucke JP. Strengthening the Reporting of Observational Studies in Epidemiology (STROBE) statement: guidelines for reporting observational studies. Bmj 2007; 335(7624): 806-8

# **eAppendix 2. Definitions and measurements for model features**

## eTable 2.1. Caregiver variables

| Assistant caregiver status | - Categorical variable: yes or no  - Definition: Is the caregiver has an assistant caregiver? |
| --- | --- |
| Current working status | - Categorical variable: yes or no  - Definition: Is the caregiver in a working condition? |
| Existence of another child with a mental disorder | - Categorical variable: yes or no  - Definition: Are there any children raised by the caregiver who have mental disorder other than the ASD patient? |
| Age of caregiver | - Continuous variable  - Definition: the age of the caregiver at the time of study |
| The order of birth | - Continuous variable  - Definition: *N* of the following sentence (the ASD patient is a *N-*th child of a caregiver) |
| Number of children | - Continuous variable  - Definition: How many children the caregiver has? |
| Minnesota Multiphasic Personality Inventory-2 (MMPI-2) clinical scales | - Continuous variables  - Description: The MMPI-2 is a validated tool for measuring one's personality and psychopathology. The MMPI-2 contains 567 items.  - Definitions of MMPI-2 clinical scales:   - Hypochondriasis: measures one's vague and non-specific complaints about bodily symptoms - Depression: measures one's depressive symptoms (poor morale, dissatisfaction for one's life, etc.) - Hysteria: measures one's poor physical health, shyness, cynicism, headaches, and neuroticism - Psychopathy: measures one's general social maladjustments - Masculinity/femininity: measures one's adherence to gender stereotypes for both men and women - Paranoia: measures one's moral self-righteousness, interpersonal sensitivity, and suspicion - Psychasthenia: measures the inability of a person to withstand a particular action or thought - Schizophrenia: measures one's tendencies toward bizarre thinking and social isolation - Hypomania: measures one's excitability, elevated but unstable mood, and flight of ideas - Social introversion: measures one's orientation (introversion or extroversion) |

## eTable 2.2. Autism spectrum disorder (ASD) patient variables

| Sex | - Categorical variable: male or female  - Definition: sex of the ASD patient |
| --- | --- |
| Family history of mental disorder | - Categorical variable: yes or no  - Definition: Is the ASD patient has a family member with any mental disorder? |
| History of the major disease | - Categorical variable: yes or no  - Definition: Is the ASD patient has suffered a disease that required operation or hospitalization? |
| Psychotropic medication status | - Categorical variable: drug-free, monotherapy of antipsychotics, combined therapy of antipsychotics, or other psychotic medication  - Definitions:   - Drug-free: the ASD patient was not taking any psychotropic medication at the time of study - Monotherapy of antipsychotics: the ASD patient was taking only one kind of antipsychotics at the time of study - Combined therapy of antipsychotics: the ASD patient was taking multiple kind of antipsychotics at the time of study - Other psychotic medication: the ASD patient was taking any psychotropic drug(s) other than antipsychotics at the time of study |
| Mode of delivery | - Categorical variable: vaginal delivery or cesarean section  - Definition: Which mode was the ASD patient born with, vaginal delivery or cesarean section? |
| Age of the patient | - Continuous variable  - Definition: the ASD patient's age at the time of study |
| Gestational age | - Continuous variable  - Definition: gestational age of the ASD patient |
| Birth weight | - Continuous variable  - Definition: the ASD patient's weight at the time of birth |
| Full-scale intelligence quotient (FSIQ) | - Continuous variable  - Definition: the ASD patient’s complete cognitive capacity at the time of study |
| Social responsiveness scale-2 (SRS-2) subscales | - Continuous variables  - Description: The SRS-2 is a validated tool for assessing symptoms that is associated with autism. It contains 65 items.  - Definitions of SRS-2 subscales   - Social awareness: measures the ASD patient's ability to understand social cues - Social cognition: measures the ASD patient's ability to interpret social behaviors - Social communication: measures the ASD patient's ability to engage in reciprocal communication in social situation - Social motivation: measures the ASD patient's motivation to join the social interaction - Autistic mannerisms: measures the ASD patient's restricted interests and stereotypy |
| Child behavior checklist (CBCL) syndrome subscales | - Continuous variables  - Description: The CBCL is a validated tool for evaluating problematic behaviors in children and often used for ASD patients in both clinical and research contexts. There are two forms: one for preschoolers (CBCL 1.5-5) and the other for school-aged children (CBCL 6-18). CBCL 1.5-5 contains 100 items, and CBCL 6-18 contains 118 items.  - Definitions of CBCL syndrome subscales (common ones between CBCL 1.5-5 and 6-18)   - Anxious/depressed: measures one's disposition to be anxious, overly concerned, or emotionally depressed - Aggressive behavior: measures one's disposition to be aggressive and rebellious - Attention problems: measures one's problems with attention and related behaviors - Somatic complaints: measures one's subjective physical symptoms without medical signs - Withdrawn: measures one's disposition to be socially inhibited and having passive attitude - Other problems: measures one's problematic behaviors that do not belong to any other syndrome subscales but appear with significant frequency   - Definitions of CBCL 1.5-5 only syndrome subscales   - Emotionally reactivity: measures one's tendency to be intensive and emotionally aroused to emotional challenges - Sleep problems: measures one's difficulties related to sleep   - Definitions of CBCL 6-18 only syndrome subscales   - Social problems: measures one's developmental problems and social immaturity - Thought problems: measures one's unrealistic and bizarre thoughts and behaviors related to them - Rule-breaking behavior: measures one's delinquent behaviors |

# **eAppendix 3. Missing value information**

|  | Overall sample, n (%) (n=496) | CBCL, n (%) | | ADHD, n (%) | |
| --- | --- | --- | --- | --- | --- |
|  |  | CBCL 1.5-5 (n=194) | CBCL 6-18 (n=302) | With (n=168) | Without (n=328) |
| Age of caregiver | **52 (10.5)** | **21 (11.0)** | **31 (10.0)** | **17 (10.1)** | **35 (10.7)** |
| Assistant caregiver status | **16 (3.2)** | **9 (4.6)** | **7 (2.3)** | **4 (2.4)** | **12 (3.7)** |
| Current working status | **58 (11.7)** | **24 (12.4)** | **34 (11.3)** | **19 (11.3)** | **39 (11.9)** |
| The order of birth | 0 (0.0) | 0 (0.0) | 0 (0.0) | 0 (0.0) | 0 (0.0) |
| Number of children | 0 (0.0) | 0 (0.0) | 0 (0.0) | 0 (0.0) | 0 (0.0) |
| Existence of another child with mental disorder | **1 (0.2)** | 0 (0.0) | **1 (0.3)** | **1 (0.6)** | 0 (0.0) |
| MMPI (T-score) |  |  |  |  |  |
| Hypochondriasis | 0 (0.0) | 0 (0.0) | 0 (0.0) | 0 (0.0) | 0 (0.0) |
| Depression | 0 (0.0) | 0 (0.0) | 0 (0.0) | 0 (0.0) | 0 (0.0) |
| Hysteria | 0 (0.0) | 0 (0.0) | 0 (0.0) | 0 (0.0) | 0 (0.0) |
| Psychopathy | 0 (0.0) | 0 (0.0) | 0 (0.0) | 0 (0.0) | 0 (0.0) |
| Masculinity/Femininity | 0 (0.0) | 0 (0.0) | 0 (0.0) | 0 (0.0) | 0 (0.0) |
| Paranoia | 0 (0.0) | 0 (0.0) | 0 (0.0) | 0 (0.0) | 0 (0.0) |
| Psychasthenia | 0 (0.0) | 0 (0.0) | 0 (0.0) | 0 (0.0) | 0 (0.0) |
| Schizophrenia | 0 (0.0) | 0 (0.0) | 0 (0.0) | 0 (0.0) | 0 (0.0) |
| Hypomania | 0 (0.0) | 0 (0.0) | 0 (0.0) | 0 (0.0) | 0 (0.0) |
| Social Introversion | 0 (0.0) | 0 (0.0) | 0 (0.0) | 0 (0.0) | 0 (0.0) |
| Age of patient | 0 (0.0) | 0 (0.0) | 0 (0.0) | 0 (0.0) | 0 (0.0) |
| Sex | 0 (0.0) | 0 (0.0) | 0 (0.0) | 0 (0.0) | 0 (0.0) |
| Family history of mental disorders | **29 (5.8)** | **19 (9.8)** | **10 (3.3)** | **7 (4.2)** | **22 (6.7)** |
| History of major disease | **8 (1.6)** | **4 (2.1)** | **4 (1.3)** | **2 (1.2)** | **6 (1.8)** |
| Psychotropic medication status | 0 (0.0) | 0 (0.0) | 0 (0.0) | 0 (0.0) | 0 (0.0) |
| Gestational age | **45 (9.1)** | **14 (7.0)** | **31 (10.0)** | **20 (11.9)** | **25 (7.6)** |
| Birth weight | **35 (7.1)** | **16 (8.0)** | **19 (6.0)** | **12 (7.1)** | **23 (7.0)** |
| Mode of delivery | **32 (6.5)** | **14 (7.2)** | **18 (6.0)** | **12 (7.1)** | **20 (6.1)** |
| FSIQ | **6 (1.2)** | **6 (3.0)** | 0 (0.0) | **1 (0.6)** | **5 (1.5)** |
| SRS (T-score) |  |  |  |  |  |
| Social awareness | 0 (0.0) | 0 (0.0) | 0 (0.0) | 0 (0.0) | 0 (0.0) |
| Social cognition | 0 (0.0) | 0 (0.0) | 0 (0.0) | 0 (0.0) | 0 (0.0) |
| Social communication | 0 (0.0) | 0 (0.0) | 0 (0.0) | 0 (0.0) | 0 (0.0) |
| Social motivation | 0 (0.0) | 0 (0.0) | 0 (0.0) | 0 (0.0) | 0 (0.0) |
| Autistic mannerisms | 0 (0.0) | 0 (0.0) | 0 (0.0) | 0 (0.0) | 0 (0.0) |
| CBCL (T-score) |  |  |  |  |  |
| Anxious/depressed | 0 (0.0) | 0 (0.0) | 0 (0.0) | 0 (0.0) | 0 (0.0) |
| Aggressive behavior | 0 (0.0) | 0 (0.0) | 0 (0.0) | 0 (0.0) | 0 (0.0) |
| Attention problems | 0 (0.0) | 0 (0.0) | 0 (0.0) | 0 (0.0) | 0 (0.0) |
| Somatic complaints | 0 (0.0) | 0 (0.0) | 0 (0.0) | 0 (0.0) | 0 (0.0) |
| Withdrawn | 0 (0.0) | 0 (0.0) | 0 (0.0) | 0 (0.0) | 0 (0.0) |
| Other problems | 0 (0.0) | 0 (0.0) | 0 (0.0) | 0 (0.0) | 0 (0.0) |
| CBCL 1.5-5 (T-score) |  |  |  |  |  |
| Emotionally reactivity | 0 (0.0) | 0 (0.0) | 0 (0.0) | 0 (0.0) | 0 (0.0) |
| Sleep problems | 0 (0.0) | 0 (0.0) | 0 (0.0) | 0 (0.0) | 0 (0.0) |
| CBCL 6-18 (T-score) |  |  |  |  |  |
| Social problems | 0 (0.0) | 0 (0.0) | 0 (0.0) | 0 (0.0) | 0 (0.0) |
| Thought problems | 0 (0.0) | 0 (0.0) | 0 (0.0) | 0 (0.0) | 0 (0.0) |
| Rule-breaking behavior | 0 (0.0) | 0 (0.0) | 0 (0.0) | 0 (0.0) | 0 (0.0) |
| PSI |  |  |  |  |  |
| Parental distress | 0 (0.0) | 0 (0.0) | 0 (0.0) | 0 (0.0) | 0 (0.0) |
| Parent-child dysfunctional interaction | 0 (0.0) | 0 (0.0) | 0 (0.0) | 0 (0.0) | 0 (0.0) |
| Difficult child | 0 (0.0) | 0 (0.0) | 0 (0.0) | 0 (0.0) | 0 (0.0) |
| Total parenting stress | 0 (0.0) | 0 (0.0) | 0 (0.0) | 0 (0.0) | 0 (0.0) |
| * Missing values were highlighted with bold  Abbreviations: ADHD=attention-deficit/hyperactivity disorder, CBCL=child behavior checklist, FSIQ=full scale intelligence quotient, MMPI=Minnesota Multiphasic Personality Inventory, PSI=parenting stress index, SD=standard deviation, SRS=social responsiveness scale | | | | | |

# **eAppendix 4. Formulas for variance inflation factor, area under the receiver operating curve, sensitivity, specificity, positive predictive value, negative predictive value, and accuracy**

**1. Variance inflation factor**

$${Variance inflation factor}_{i}= \frac{1}{1-R_{i}^{2}}$$

R_i_^2^ = the coefficient of determination for the regression model with the *i*th independent variable on the remaining ones

**2. Area under the receiver operating curve (AUROC)**

$$AUROC= 1 - \int_{0}^{1} (1-TPR)\times FPR dTPR$$

TPR = true positive rate

FPR = false positive rate

**3. Sensitivity**

$$Sensitivity= \frac{TP}{TP+FN}$$

TP = true positive

FN = false negative

**4. Specificity**

$$Specificity= \frac{TN}{TN+FP}$$

TN = true negative

FP = false positive

**5. Positive predictive value (PPV)**

$$PPV= \frac{TP}{TP+FP}$$

TP = true positive

FP = false positive

**6. Negative predictive value (NPV)**

$$NPV= \frac{TN}{TN+FN}$$

TN = true negative

FN = false negative

​

**6. Accuracy**

$$Accuracy= \frac{TP+TN}{TP+TN+FP+FN}$$

TP = true positive

TN = true negative

FP = false positive

FN = false negative

# **eAppendix 5. Variance inflation factor calculation for continuous features**

|  | Overall sample, n (%) (n=496) | CBCL, n (%) | | ADHD, n (%) | |
| --- | --- | --- | --- | --- | --- |
|  |  | CBCL 1.5-5 (n=194) | CBCL 6-18 (n=302) | With (n=168) | Without (n=328) |
| Age of caregiver | 1.328100103 | 1.21683276 | 1.32154809 | 1.47818223 | 1.38412588 |
| The order of birth | 1.38464456 | 1.63747073 | 1.43822986 | 1.42872323 | 1.47480947 |
| Number of children | 1.32141622 | 1.65917738 | 1.29162735 | 1.30243065 | 1.49131966 |
| MMPI (T-score) |  |  |  |  |  |
| Hypochondriasis | 2.69030249 | 2.98090466 | 2.80877733 | 2.82202298 | 2.79836466 |
| Depression | 3.29692133 | 2.99782422 | 3.74881065 | 3.77784991 | 3.32863437 |
| Hysteria | 3.19511041 | 3.30219083 | 3.25589665 | 3.296105 | 3.33589341 |
| Psychopathy | 2.07683498 | 1.84055648 | 2.40879049 | 2.15998201 | 2.18378947 |
| Masculinity/Femininity | 1.15345209 | 1.19943478 | 1.33395136 | 1.48402914 | 1.16764758 |
| Paranoia | 2.0538148 | 1.86845747 | 2.40129807 | 3.05332692 | 1.89908027 |
| Psychasthenia | 3.77436547 | 4.85216277 | 3.71274271 | 4.9251616 | 3.73292488 |
| Schizophrenia | 3.7725289 | 4.74285693 | 3.83389231 | 4.32794182 | 3.78480263 |
| Hypomania | 1.57600991 | 1.95262512 | 1.52873142 | 1.46403474 | 1.76890074 |
| Social Introversion | 2.72548731 | 2.88021529 | 2.87127723 | 3.0770664 | 2.77122783 |
| Age of patient | 1.7343944 | 1.19492412 | 1.48882369 | 1.59969954 | 1.84480557 |
| Gestational age | 1.65541438 | 1.75072794 | 1.7172887 | 1.5598499 | 1.79395961 |
| Birth weight | 1.61000863 | 1.75115103 | 1.61373783 | 1.51611491 | 1.75945733 |
| FSIQ | 1.48300538 | 1.45934134 | 1.4473346 | 1.33707042 | 1.45808249 |
| SRS (T-score) |  |  |  |  |  |
| Social awareness | 2.39427667 | 2.573166 | 2.63901968 | 2.88009649 | 2.37466077 |
| Social cognition | 3.23991034 | 2.89247688 | 3.89125635 | 3.5315031 | 3.311269 |
| Social communication | 3.96908575 | 5.07636924 | 3.93299892 | 4.86340139 | 4.04144448 |
| Social motivation | 2.55758267 | 2.51288292 | 2.99993135 | 2.49234013 | 2.74344348 |
| Autistic mannerisms | 2.47749655 | 2.58875341 | 2.87654823 | 2.93380698 | 2.50259427 |
| CBCL (T-score) |  |  |  |  |  |
| Anxious/depressed | 2.15825398 | 2.40611544 | 2.23088472 | 2.60734014 | 2.05765852 |
| Aggressive behavior | 2.28356794 | 2.48881085 | 3.175078 | 2.69966726 | 2.17146278 |
| Attention problems | 1.9951402 | 1.77192373 | 2.53021872 | 2.50741092 | 1.94874762 |
| Somatic complaints | 1.59829161 | 1.64466112 | 1.77332942 | 1.87609899 | 1.60462628 |
| Withdrawn | 2.11811194 | 2.42107869 | 2.41777019 | 1.97138492 | 2.29429633 |
| Other problems | 2.10224083 | 3.37041151 | 2.1088584 | 1.97946259 | 2.34168061 |
| CBCL 1.5-5 (T-score) |  |  |  |  |  |
| Emotionally reactivity |  | 3.69679062 |  |  |  |
| Sleep problems |  | 1.64406101 |  |  |  |
| CBCL 6-18 (T-score) |  |  |  |  |  |
| Social problems |  |  | 2.3922338 |  |  |
| Thought problems |  |  | 2.28530668 |  |  |
| Rule-breaking behavior |  |  | 2.31402069 |  |  |
| Abbreviations: ADHD=attention-deficit/hyperactivity disorder, CBCL=child behavior checklist, FSIQ=full scale intelligence quotient, MMPI=Minnesota Multiphasic Personality Inventory, PSI=parenting stress index, SD=standard deviation, SRS=social responsiveness scale | | | | | |

# **eAppendix 6. Model information and detailed results (Sample: Overall sample [n=496], Outcome: Parental distress)**

## eTable 6.1. Model features (Variables are on the second columns)

| **Caregiver variables** | - Assistant caregiver status [Yes/No]  - Current working status [Yes/No]  - Minnesota Multiphasic Personality Inventory (MMPI) Hypochondriasis (T-score)  - MMPI Depression (T-score)  - MMPI Hysteria (T-score)  - MMPI Psychopathy (T-score)  - MMPI Masculinity/Femininity (T-score)  - MMPI Paranoia (T-score)  - MMPI Psychasthenia (T-score)  - MMPI Schizophrenia (T-score)  - MMPI Hypomania (T-score)  - MMPI Social Introversion (T-score)  - The order of birth  - Number of children |
| --- | --- |
| **ASD patient variables** | - Age  - Sex [Male/Female]  - Family history of mental disorder [Yes/No]  - History of major disease [Yes/No]  - Psychotropic medication status [Drug free/Monotherapy of antipsychotics/Combined therapy of antipsychotics/Other psychotic medication]  - Gestational age  - Birth weight  - Mode of delivery [Vaginal delivery/Caesarean section]  - Existence of another child with mental disorder [Yes/No]  - Full-Scale Intelligence Quotient (FSIQ)  - Social responsiveness scale (SRS) Social awareness (T-score)  - SRS Social cognition (T-score)  - SRS Social communication (T-score)  - SRS Social motivation (T-score)  - SRS Autistic mannerisms (T-score)  - Child Behavior Checklist (CBCL) Anxious/depressed (T-score)  - CBCL Withdrawn/depressed (T-score)  - CBCL Somatic complaints (T-score)  - CBCL Attention problems (T-score)  - CBCL Aggressive behavior (T-score)  - CBCL Other problems (T-score) |

## eTable 6.2. Performance of each model on the training set

|  | **RF model** | **XGBoost model** | **LR model** | **SVM model** |
| --- | --- | --- | --- | --- |
| **ROC AUC (95% CI)** | 0.945 (0.923-0.965) | 0.915 (0.886-0.941) | 0.848 (0.806-0.888) | 0.843 (0.799-0.884) |
| **Sensitivity (95% CI)** | 0.591 (0.500-0.681) | 0.539 (0.448-0.631) | 0.496 (0.406-0.587) | 0.487 (0.398-0.578) |
| **Specificity (95% CI)** | 0.972 (0.951-0.989) | 0.961 (0.937-0.982) | 0.932 (0.901-0.960) | 0.936 (0.906-0.964) |
| **PPV (95% CI)** | 0.895 (0.822-0.958) | 0.849 (0.764-0.927) | 0.750 (0.649-0.844) | 0.757 (0.656-0.851) |
| **NPV (95% CI)** | 0.853 (0.814-0.891) | 0.836 (0.796-0.875) | 0.819 (0.776-0.860) | 0.817 (0.774-0.858) |
| **Accuracy (95% CI)** | 0.861 (0.828-0.894) | 0.838 (0.803-0.874) | 0.806 (0.765-0.843) | 0.806 (0.765-0.843) |
| Abbreviations: AUC=area under curve, CI=confidence interval, NA=not available, NPV=negative predictive value, LR=logistic regression, RF=random forest, ROC=receiver operating characteristic, PPV=positive predictive value, SVM=support vector machine, XGBoost=Extreme Gradient Boosting. | | | | |

## eTable 6.3. Performance of each model on the test set

|  | **RF model** | **XGBoost model** | **LR model** | **SVM model** |
| --- | --- | --- | --- | --- |
| **ROC AUC (95% CI)** | 0.831 (0.740-0.910) | 0.802 (0.699-0.890) | 0.828 (0.731-0.911) | 0.809 (0.711-0.894) |
| **Sensitivity (95% CI)** | 0.655 (0.478-0.821) | 0.483 (0.296-0.667) | 0.586 (0.400-0.769) | 0.552 (0.367-0.735) |
| **Specificity (95% CI)** | 0.859 (0.773-0.934) | 0.859 (0.773-0.933) | 0.845 (0.757-0.926) | 0.887 (0.808-0.957) |
| **PPV (95% CI)** | 0.655 (0.478-0.826) | 0.583 (0.375-0.781) | 0.607 (0.419-0.789) | 0.667 (0.467-0.857) |
| **NPV (95% CI)** | 0.859 (0.773-0.934) | 0.803 (0.707-0.890) | 0.833 (0.743-0.917) | 0.829 (0.739-0.910) |
| **Accuracy (95% CI)** | 0.800 (0.720-0.880) | 0.750 (0.660-0.830) | 0.770 (0.690-0.850) | 0.790 (0.710-0.870) |
| Abbreviations: AUC=area under curve, CI=confidence interval, NA=not available, NPV=negative predictive value, LR=logistic regression, RF=random forest, ROC=receiver operating characteristic, PPV=positive predictive value, SVM=support vector machine, XGBoost=Extreme Gradient Boosting. | | | | |

##

## eFigure 6.4. SHapley Additive exPlanations (SHAP) summary plot for RF and XGBoost models (only top 20 predictors were presented)

| <RF model> | <XGBoost model> |
| --- | --- |
| 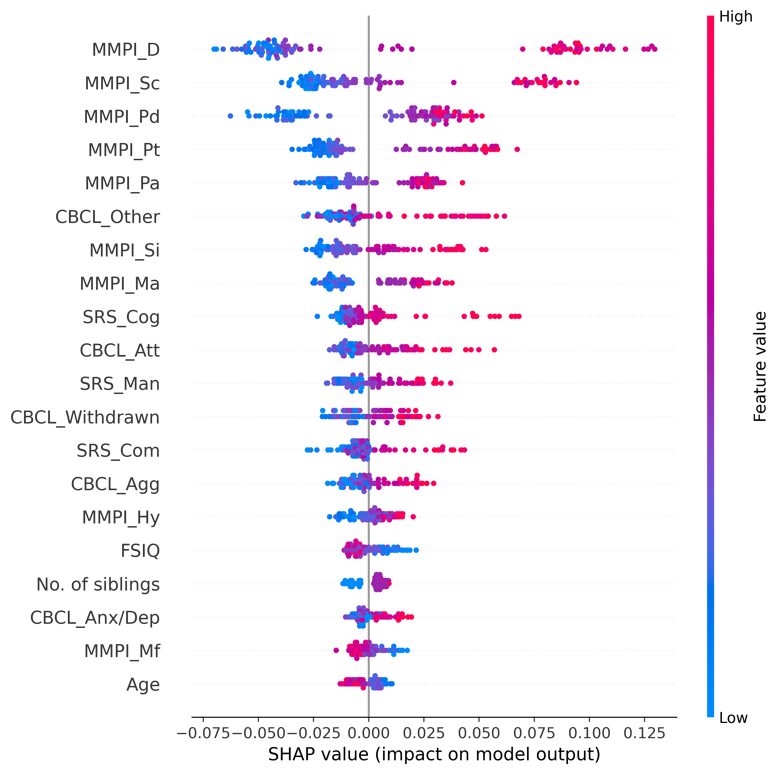 | 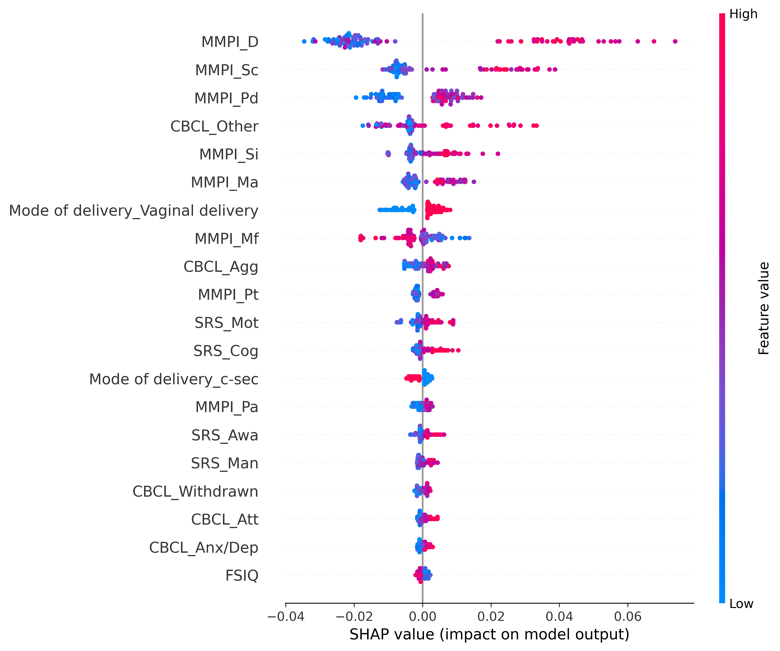 |

## eFigure 6.5. Receiver operating characteristic curves (ROC) for the training and test set

| <Training set> | <Test set> |
| --- | --- |
| 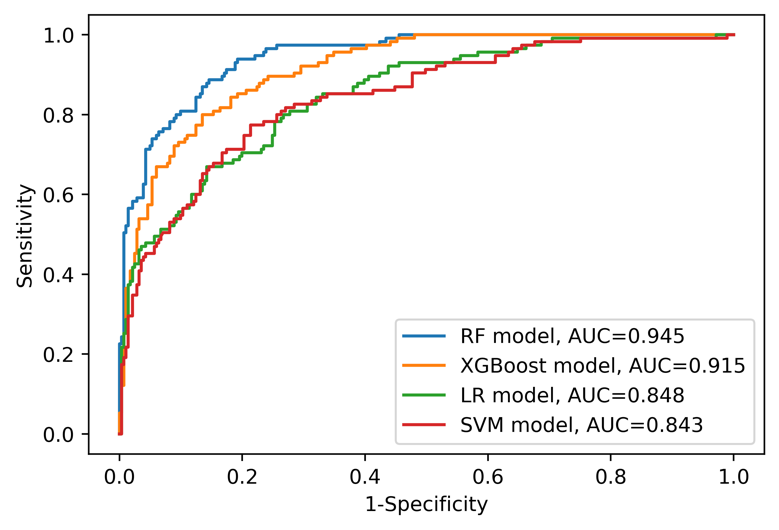 | 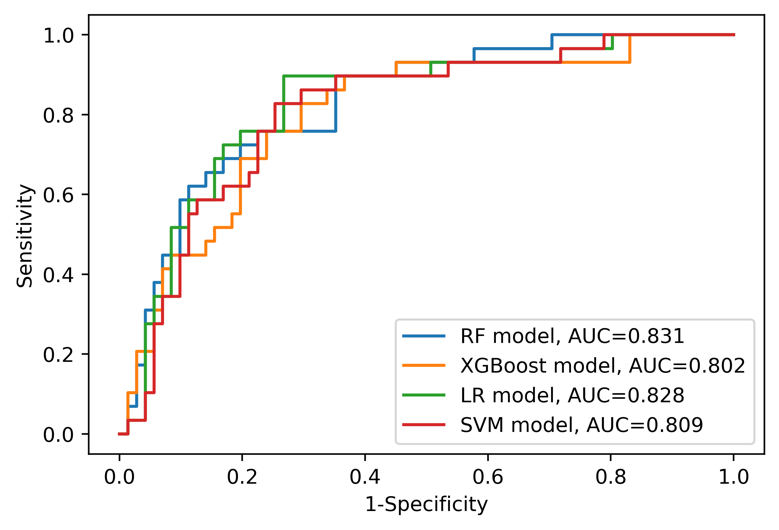 |

Abbreviations: AUC=area under curve, LR=logistic regression, RF=random forest, SVM=support vector machine, XGBoost=Extreme Gradient Boosting.

# **eAppendix 7. Model information and detailed results (Sample: Overall sample [n=496] Outcome: Parent-child dysfunctional interaction)**

## eTable 7.1. Model features (Variables are on the second columns)

| **Caregiver variables** | - Assistant caregiver status [Yes/No]  - Current working status [Yes/No]  - Minnesota Multiphasic Personality Inventory (MMPI) Hypochondriasis (T-score)  - MMPI Depression (T-score)  - MMPI Hysteria (T-score)  - MMPI Psychopathy (T-score)  - MMPI Masculinity/Femininity (T-score)  - MMPI Paranoia (T-score)  - MMPI Psychasthenia (T-score)  - MMPI Schizophrenia (T-score)  - MMPI Hypomania (T-score)  - MMPI Social Introversion (T-score)  - The order of birth  - Number of children |
| --- | --- |
| **ASD patient variables** | - Age  - Sex [Male/Female]  - Family history of mental disorder [Yes/No]  - History of major disease [Yes/No]  - Psychotropic medication status [Drug free/Monotherapy of antipsychotics/Combined therapy of antipsychotics/Other psychotic medication]  - Gestational age  - Birth weight  - Mode of delivery [Vaginal delivery/Caesarean section]  - Existence of another child with mental disorder [Yes/No]  - Full-Scale Intelligence Quotient (FSIQ)  - Social responsiveness scale (SRS) Social awareness (T-score)  - SRS Social cognition (T-score)  - SRS Social communication (T-score)  - SRS Social motivation (T-score)  - SRS Autistic mannerisms (T-score)  - Child Behavior Checklist (CBCL) Anxious/depressed (T-score)  - CBCL Withdrawn/depressed (T-score)  - CBCL Somatic complaints (T-score)  - CBCL Attention problems (T-score)  - CBCL Aggressive behavior (T-score)  - CBCL Other problems (T-score) |

## eTable 7.2. Performance of each model on the training set

|  | **RF model** | **XGBoost model** | **LR model** | **SVM model** |
| --- | --- | --- | --- | --- |
| **ROC AUC (95% CI)** | 0.960 (0.943-0.975) | 0.904 (0.873-0.932) | 0.805 (0.761-0.846) | 0.800 (0.755-0.842) |
| **Sensitivity (95% CI)** | 0.826 (0.766-0.881) | 0.725 (0.655-0.790) | 0.557 (0.480-0.633) | 0.545 (0.468-0.621) |
| **Specificity (95% CI)** | 0.943 (0.912-0.972) | 0.908 (0.870-0.945) | 0.830 (0.780-0.877) | 0.838 (0.790-0.885) |
| **PPV (95% CI)** | 0.914 (0.868-0.957) | 0.852 (0.791-0.909) | 0.705 (0.627-0.781) | 0.711 (0.632-0.789) |
| **NPV (95% CI)** | 0.882 (0.839-0.920) | 0.819 (0.770-0.865) | 0.720 (0.665-0.772) | 0.716 (0.662-0.769) |
| **Accuracy (95% CI)** | 0.894 (0.864-0.924) | 0.831 (0.793-0.866) | 0.715 (0.669-0.758) | 0.715 (0.669-0.760) |
| Abbreviations: AUC=area under curve, CI=confidence interval, NA=not available, NPV=negative predictive value, LR=logistic regression, RF=random forest, ROC=receiver operating characteristic, PPV=positive predictive value, SVM=support vector machine, XGBoost=Extreme Gradient Boosting. | | | | |

##

## eTable 7.3. Performance of each model on the test set

|  | **RF model** | **XGBoost model** | **LR model** | **SVM model** |
| --- | --- | --- | --- | --- |
| **ROC AUC (95% CI)** | 0.791 (0.694-0.878) | 0.808 (0.715-0.891) | 0.814 (0.720-0.897) | 0.814 (0.720-0.896) |
| **Sensitivity (95% CI)** | 0.595 (0.444-0.744) | 0.667 (0.522-0.810) | 0.619 (0.469-0.767) | 0.595 (0.444-0.744) |
| **Specificity (95% CI)** | 0.828 (0.723-0.923) | 0.845 (0.745-0.933) | 0.828 (0.724-0.918) | 0.828 (0.724-0.918) |
| **PPV (95% CI)** | 0.714 (0.559-0.862) | 0.757 (0.610-0.892) | 0.722 (0.568-0.865) | 0.714 (0.559-0.860) |
| **NPV (95% CI)** | 0.738 (0.630-0.842) | 0.778 (0.672-0.877) | 0.750 (0.641-0.855) | 0.738 (0.629-0.844) |
| **Accuracy (95% CI)** | 0.730 (0.640-0.810) | 0.770 (0.680-0.850) | 0.740 (0.650-0.820) | 0.730 (0.640-0.810) |
| Abbreviations: AUC=area under curve, CI=confidence interval, NA=not available, NPV=negative predictive value, LR=logistic regression, RF=random forest, ROC=receiver operating characteristic, PPV=positive predictive value, SVM=support vector machine, XGBoost=Extreme Gradient Boosting. | | | | |

##

## eFigure 7.4. SHapley Additive exPlanations (SHAP) summary plot for RF and XGBoost models (only top 20 predictors were presented)

| <RF model> | <XGBoost model> |
| --- | --- |
| 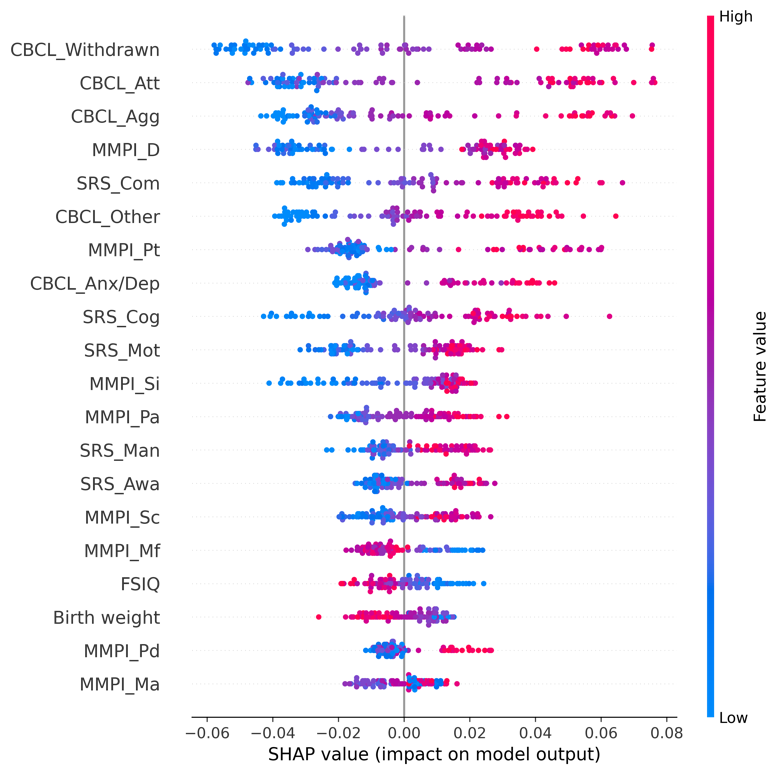 | 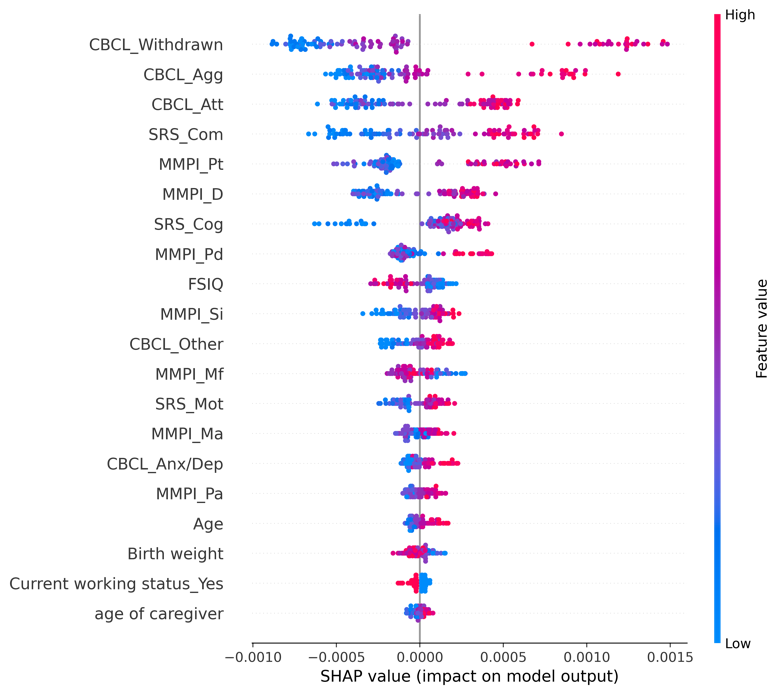 |

## eFigure 7.5. Receiver operating characteristic curves (ROC) for the training and test set

| <Training set> | <Test set> |
| --- | --- |
| 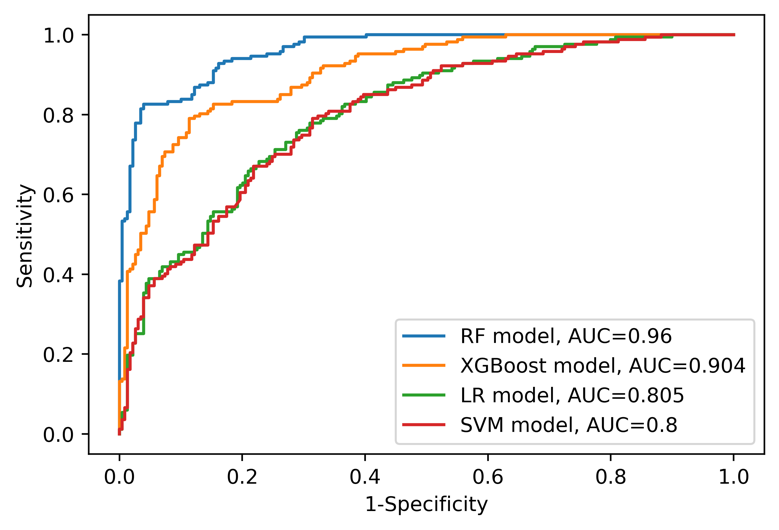 | 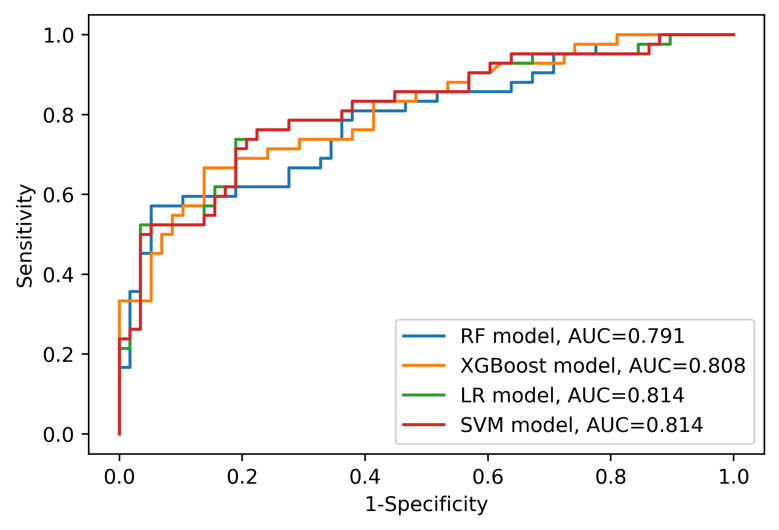 |

Abbreviations: AUC=area under curve, LR=logistic regression, RF=random forest, SVM=support vector machine, XGBoost=Extreme Gradient Boosting.

# **eAppendix 8. Model information and detailed results (Sample: Overall sample [n=496], Outcome: Difficult child)**

## eTable 8.1. Model features (Variables are on the second columns)

| **Caregiver variables** | - Assistant caregiver status [Yes/No]  - Current working status [Yes/No]  - Minnesota Multiphasic Personality Inventory (MMPI) Hypochondriasis (T-score)  - MMPI Depression (T-score)  - MMPI Hysteria (T-score)  - MMPI Psychopathy (T-score)  - MMPI Masculinity/Femininity (T-score)  - MMPI Paranoia (T-score)  - MMPI Psychasthenia (T-score)  - MMPI Schizophrenia (T-score)  - MMPI Hypomania (T-score)  - MMPI Social Introversion (T-score)  - The order of birth  - Number of children |
| --- | --- |
| **ASD patient variables** | - Age  - Sex [Male/Female]  - Family history of mental disorder [Yes/No]  - History of major disease [Yes/No]  - Psychotropic medication status [Drug free/Monotherapy of antipsychotics/Combined therapy of antipsychotics/Other psychotic medication]  - Gestational age  - Birth weight  - Mode of delivery [Vaginal delivery/Caesarean section]  - Existence of another child with mental disorder [Yes/No]  - Full-Scale Intelligence Quotient (FSIQ)  - Social responsiveness scale (SRS) Social awareness (T-score)  - SRS Social cognition (T-score)  - SRS Social communication (T-score)  - SRS Social motivation (T-score)  - SRS Autistic mannerisms (T-score)  - Child Behavior Checklist (CBCL) Anxious/depressed (T-score)  - CBCL Withdrawn/depressed (T-score)  - CBCL Somatic complaints (T-score)  - CBCL Attention problems (T-score)  - CBCL Aggressive behavior (T-score)  - CBCL Other problems (T-score) |

## eTable 8.2. Performance of each model on the training set

|  | **RF model** | **XGBoost model** | **LR model** | **SVM model** |
| --- | --- | --- | --- | --- |
| **ROC AUC (95% CI)** | 0.988 (0.980-0.994) | 0.889 (0.855-0.919) | 0.837 (0.796-0.875) | 0.790 (0.744-0.833) |
| **Sensitivity (95% CI)** | 0.928 (0.888-0.963) | 0.761 (0.697-0.821) | 0.678 (0.608-0.744) | 0.639 (0.569-0.707) |
| **Specificity (95% CI)** | 0.949 (0.917-0.976) | 0.866 (0.819-0.909) | 0.833 (0.783-0.881) | 0.806 (0.752-0.856) |
| **PPV (95% CI)** | 0.938 (0.901-0.971) | 0.825 (0.765-0.881) | 0.772 (0.705-0.835) | 0.732 (0.662-0.800) |
| **NPV (95% CI)** | 0.940 (0.907-0.970) | 0.813 (0.762-0.863) | 0.756 (0.701-0.809) | 0.728 (0.671-0.782) |
| **Accuracy (95% CI)** | 0.939 (0.914-0.962) | 0.818 (0.780-0.856) | 0.763 (0.720-0.806) | 0.730 (0.684-0.773) |
| Abbreviations: AUC=area under curve, CI=confidence interval, NA=not available, NPV=negative predictive value, LR=logistic regression, RF=random forest, ROC=receiver operating characteristic, PPV=positive predictive value, SVM=support vector machine, XGBoost=Extreme Gradient Boosting. | | | | |

## eTable 8.3. Performance of each model on the test set

|  | **RF model** | **XGBoost model** | **LR model** | **SVM model** |
| --- | --- | --- | --- | --- |
| **ROC AUC (95% CI)** | 0.813 (0.724-0.891) | 0.805 (0.716-0.884) | 0.801 (0.711-0.882) | 0.740 (0.638-0.831) |
| **Sensitivity (95% CI)** | 0.733 (0.600-0.857) | 0.711 (0.571-0.837) | 0.600 (0.452-0.739) | 0.578 (0.429-0.721) |
| **Specificity (95% CI)** | 0.745 (0.627-0.857) | 0.782 (0.667-0.887) | 0.782 (0.667-0.887) | 0.745 (0.627-0.857) |
| **PPV (95% CI)** | 0.702 (0.568-0.827) | 0.727 (0.590-0.857) | 0.692 (0.543-0.833) | 0.650 (0.500-0.795) |
| **NPV (95% CI)** | 0.774 (0.660-0.880) | 0.768 (0.655-0.875) | 0.705 (0.588-0.815) | 0.683 (0.564-0.797) |
| **Accuracy (95% CI)** | 0.740 (0.650-0.820) | 0.750 (0.660-0.830) | 0.700 (0.610-0.790) | 0.670 (0.580-0.760) |
| Abbreviations: AUC=area under curve, CI=confidence interval, NA=not available, NPV=negative predictive value, LR=logistic regression, RF=random forest, ROC=receiver operating characteristic, PPV=positive predictive value, SVM=support vector machine, XGBoost=Extreme Gradient Boosting. | | | | |

## eFigure 8.4. SHapley Additive exPlanations (SHAP) summary plot for RF and XGBoost models (only top 20 predictors were presented)

| <RF model> | <XGBoost model> |
| --- | --- |
| 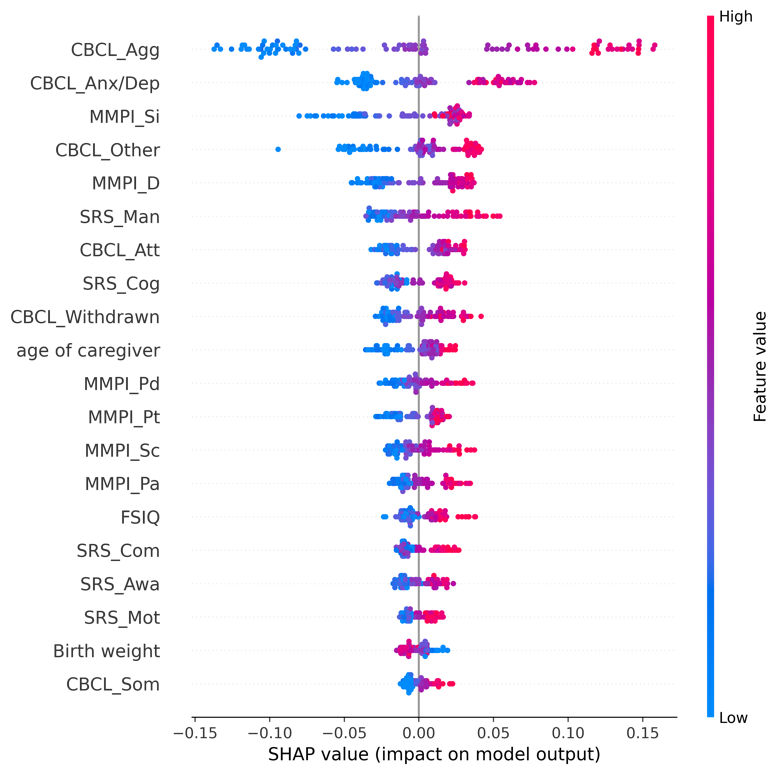 | 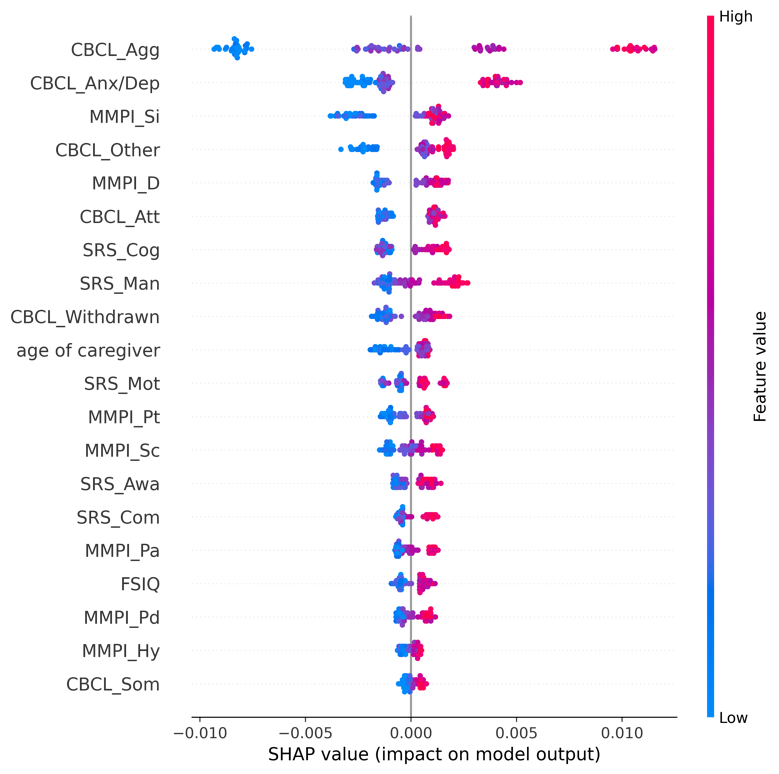 |

## eFigure 8.5. Receiver operating characteristic curves (ROC) for the training and test set

| <Training set> | <Test set> |
| --- | --- |
| 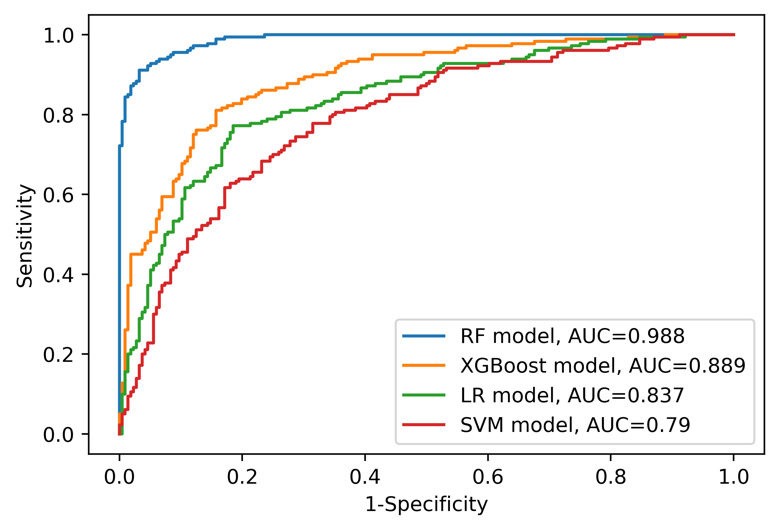 | 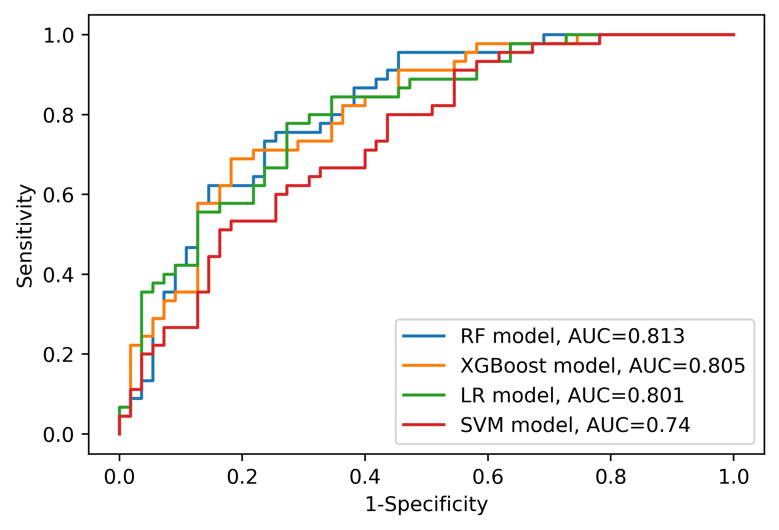 |

Abbreviations: AUC=area under curve, LR=logistic regression, RF=random forest, SVM=support vector machine, XGBoost=Extreme Gradient Boosting.

# **eAppendix 9. Model information and detailed results (Sample: Overall sample [n=496], Outcome: Total parenting stress)**

## eTable 9.1. Model features (Variables are on the second columns)

| **Caregiver variables** | - Assistant caregiver status [Yes/No]  - Current working status [Yes/No]  - Minnesota Multiphasic Personality Inventory (MMPI) Hypochondriasis (T-score)  - MMPI Depression (T-score)  - MMPI Hysteria (T-score)  - MMPI Psychopathy (T-score)  - MMPI Masculinity/Femininity (T-score)  - MMPI Paranoia (T-score)  - MMPI Psychasthenia (T-score)  - MMPI Schizophrenia (T-score)  - MMPI Hypomania (T-score)  - MMPI Social Introversion (T-score)  - The order of birth  - Number of children |
| --- | --- |
| **ASD patient variables** | - Age  - Sex [Male/Female]  - Family history of mental disorder [Yes/No]  - History of major disease [Yes/No]  - Psychotropic medication status [Drug free/Monotherapy of antipsychotics/Combined therapy of antipsychotics/Other psychotic medication]  - Gestational age  - Birth weight  - Mode of delivery [Vaginal delivery/Caesarean section]  - Existence of another child with mental disorder [Yes/No]  - Full-Scale Intelligence Quotient (FSIQ)  - Social responsiveness scale (SRS) Social awareness (T-score)  - SRS Social cognition (T-score)  - SRS Social communication (T-score)  - SRS Social motivation (T-score)  - SRS Autistic mannerisms (T-score)  - Child Behavior Checklist (CBCL) Anxious/depressed (T-score)  - CBCL Withdrawn/depressed (T-score)  - CBCL Somatic complaints (T-score)  - CBCL Attention problems (T-score)  - CBCL Aggressive behavior (T-score)  - CBCL Other problems (T-score) |

## eTable 9.2. Performance of each model on the training set

|  | **RF model** | **XGBoost model** | **LR model** | **SVM model** |
| --- | --- | --- | --- | --- |
| **ROC AUC (95% CI)** | 0.958 (0.940-0.974) | 0.904 (0.874-0.931) | 0.839 (0.800-0.876) | 0.859 (0.823-0.893) |
| **Sensitivity (95% CI)** | 0.848 (0.796-0.899) | 0.806 (0.748-0.861) | 0.702 (0.635-0.766) | 0.738 (0.675-0.800) |
| **Specificity (95% CI)** | 0.917 (0.877-0.953) | 0.859 (0.809-0.905) | 0.805 (0.749-0.859) | 0.824 (0.772-0.875) |
| **PPV (95% CI)** | 0.905 (0.861-0.946) | 0.842 (0.787-0.893) | 0.770 (0.706-0.832) | 0.797 (0.736-0.854) |
| **NPV (95% CI)** | 0.866 (0.819-0.911) | 0.826 (0.774-0.877) | 0.743 (0.684-0.801) | 0.772 (0.714-0.827) |
| **Accuracy (95% CI)** | 0.884 (0.851-0.914) | 0.833 (0.795-0.869) | 0.755 (0.712-0.798) | 0.783 (0.742-0.823) |
| Abbreviations: AUC=area under curve, CI=confidence interval, NA=not available, NPV=negative predictive value, LR=logistic regression, RF=random forest, ROC=receiver operating characteristic, PPV=positive predictive value, SVM=support vector machine, XGBoost=Extreme Gradient Boosting. | | | | |

## eTable 9.3. Performance of each model on the test set

|  | **RF model** | **XGBoost model** | **LR model** | **SVM model** |
| --- | --- | --- | --- | --- |
| **ROC AUC (95% CI)** | 0.862 (0.783-0.930) | 0.848 (0.764-0.921) | 0.861 (0.779-0.930) | 0.854 (0.772-0.924) |
| **Sensitivity (95% CI)** | 0.708 (0.578-0.833) | 0.729 (0.600-0.848) | 0.792 (0.673-0.900) | 0.708 (0.577-0.833) |
| **Specificity (95% CI)** | 0.865 (0.764-0.951) | 0.865 (0.764-0.951) | 0.827 (0.717-0.923) | 0.808 (0.698-0.909) |
| **PPV (95% CI)** | 0.829 (0.707-0.936) | 0.833 (0.714-0.939) | 0.809 (0.690-0.915) | 0.773 (0.644-0.891) |
| **NPV (95% CI)** | 0.763 (0.650-0.864) | 0.776 (0.661-0.875) | 0.811 (0.698-0.909) | 0.750 (0.630-0.855) |
| **Accuracy (95% CI)** | 0.790 (0.710-0.870) | 0.800 (0.720-0.870) | 0.810 (0.730-0.880) | 0.760 (0.670-0.840) |
| Abbreviations: AUC=area under curve, CI=confidence interval, NA=not available, NPV=negative predictive value, LR=logistic regression, RF=random forest, ROC=receiver operating characteristic, PPV=positive predictive value, SVM=support vector machine, XGBoost=Extreme Gradient Boosting. | | | | |

## eFigure 9.4. SHapley Additive exPlanations (SHAP) summary plot for RF and XGBoost models (only top 20 predictors were presented)

| <RF model> | <XGBoost model> |
| --- | --- |
| 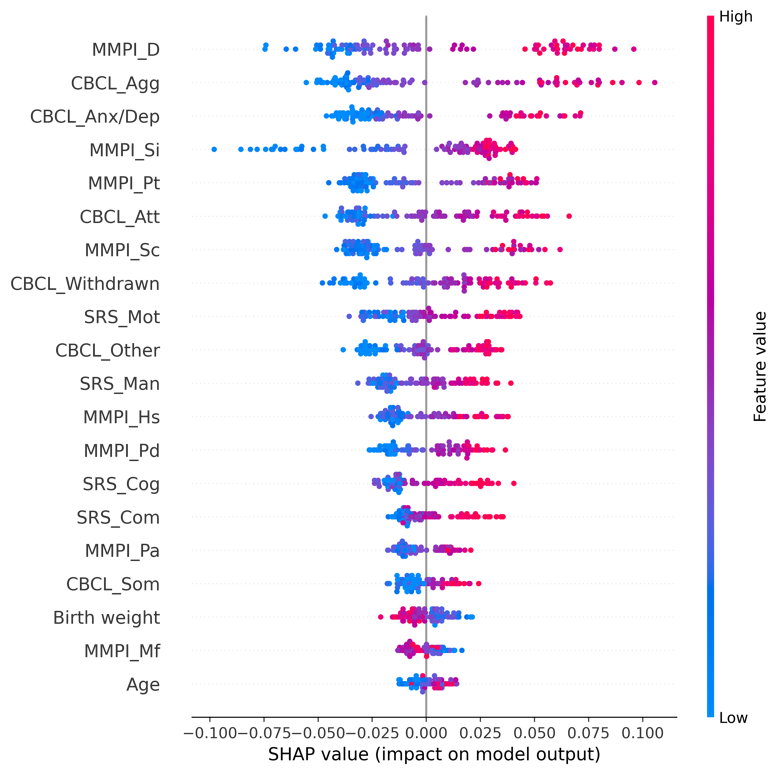 | 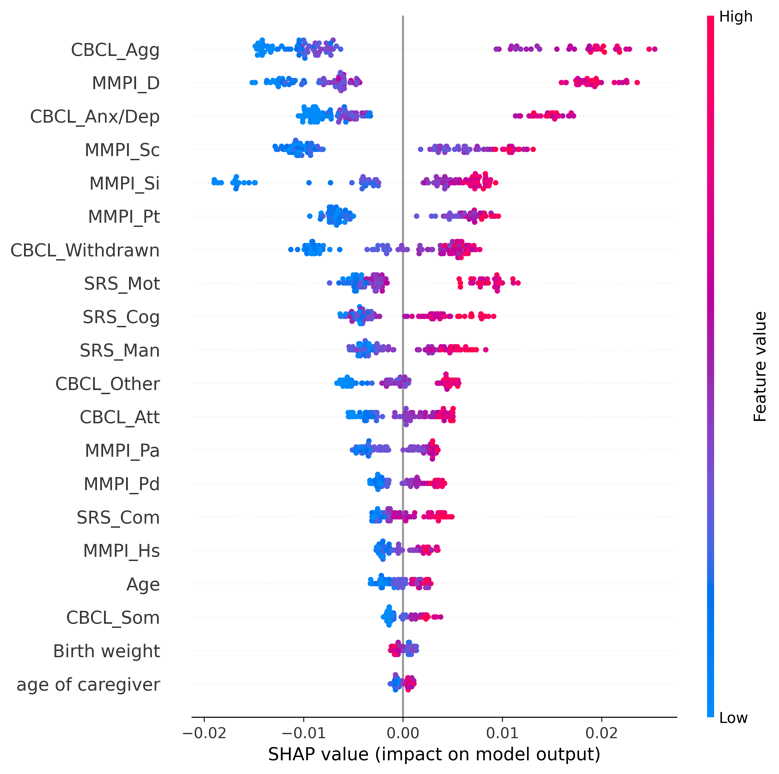 |

## eFigure 9.5. Receiver operating characteristic curves (ROC) for the training and test set

| <Training set> | <Test set> |
| --- | --- |
| 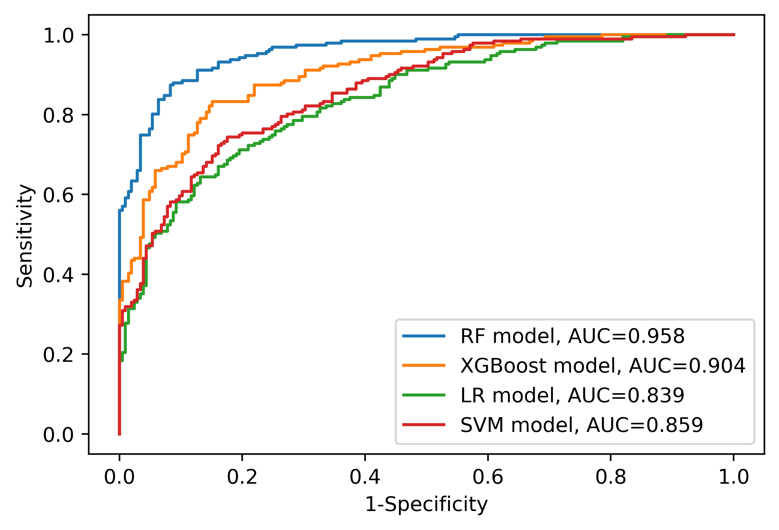 | 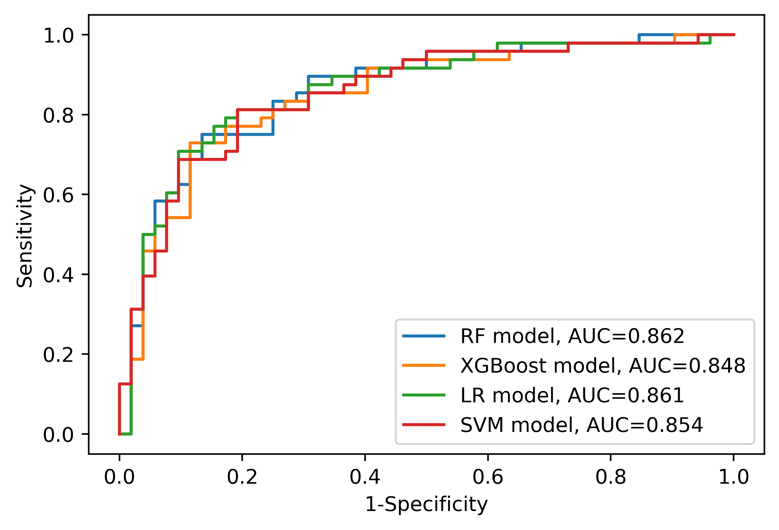 |

Abbreviations: AUC=area under curve, LR=logistic regression, RF=random forest, SVM=support vector machine, XGBoost=Extreme Gradient Boosting.

# **eAppendix 10. Model information and detailed results (Sample: CBCL 1.5-5 sample [n=194], Outcome: Parental distress)**

## eTable 10.1. Model features (Variables are on the second columns)

| **Caregiver variables** | - Assistant caregiver status [Yes/No]  - Current working status [Yes/No]  - Minnesota Multiphasic Personality Inventory (MMPI) Hypochondriasis (T-score)  - MMPI Depression (T-score)  - MMPI Hysteria (T-score)  - MMPI Psychopathy (T-score)  - MMPI Masculinity/Femininity (T-score)  - MMPI Paranoia (T-score)  - MMPI Psychasthenia (T-score)  - MMPI Schizophrenia (T-score)  - MMPI Hypomania (T-score)  - MMPI Social Introversion (T-score)  - The order of birth  - Number of children |
| --- | --- |
| **ASD patient variables** | - Age  - Sex [Male/Female]  - Family history of mental disorder [Yes/No]  - History of major disease [Yes/No]  - Psychotropic medication status [Drug free/Monotherapy of antipsychotics/Combined therapy of antipsychotics/Other psychotic medication]  - Gestational age  - Birth weight  - Mode of delivery [Vaginal delivery/Caesarean section]  - Existence of another child with mental disorder [Yes/No] [Yes/No]  - Full-Scale Intelligence Quotient (FSIQ)  - Social responsiveness scale (SRS) Social awareness (T-score)  - SRS Social cognition (T-score)  - SRS Social communication (T-score)  - SRS Social motivation (T-score)  - SRS Autistic mannerisms (T-score)  - Child Behavior Checklist (CBCL) Emotionally reactivity (T-score)  - CBCL Anxious/depressed (T-score)  - CBCL Somatic complaints (T-score)  - CBCL Withdrawn/depressed (T-score)  - CBCL Sleep problems (T-score)  - CBCL Attention problems (T-score)  - CBCL Aggressive behavior (T-score)  - CBCL Other problems (T-score) |

## eTable 10.2. Performance of each model on the training set

|  | **RF model** | **XGBoost model** | **LR model** | **SVM model** |
| --- | --- | --- | --- | --- |
| **ROC AUC (95% CI)** | 0.954 (0.921-0.981) | 0.862 (0.799-0.917) | 0.815 (0.740-0.882) | 0.937 (0.896-0.97) |
| **Sensitivity (95% CI)** | 0.574 (0.432-0.714) | 0.234 (0.120-0.360) | 0.128 (0.041-0.231) | 0.000 (0.000-0.000) |
| **Specificity (95% CI)** | 0.972 (0.937-1.000) | 0.972 (0.938-1.000) | 0.991 (0.970-1.000) | 1.000 (1.000-1.000) |
| **PPV (95% CI)** | 0.900 (0.778-1.000) | 0.786 (0.538-1.000) | 0.857 (0.500-1.000) | NA |
| **NPV (95% CI)** | 0.840 (0.771-0.902) | 0.745 (0.671-0.816) | 0.723 (0.649-0.795) | 0.697 (0.619-0.768) |
| **Accuracy (95% CI)** | 0.852 (0.794-0.903) | 0.748 (0.677-0.813) | 0.729 (0.658-0.800) | 0.697 (0.619-0.768) |
| Abbreviations: AUC=area under curve, CI=confidence interval, NA=not available, NPV=negative predictive value, LR=logistic regression, RF=random forest, ROC=receiver operating characteristic, PPV=positive predictive value, SVM=support vector machine, XGBoost=Extreme Gradient Boosting. | | | | |

## eTable 10.3. Performance of each model on the test set

|  | **RF model** | **XGBoost model** | **LR model** | **SVM model** |
| --- | --- | --- | --- | --- |
| **ROC AUC (95% CI)** | 0.861 (0.708-0.976) | 0.889 (0.760-0.982) | 0.846 (0.673-0.981) | 0.815 (0.617-0.969) |
| **Sensitivity (95% CI)** | 0.583 (0.286-0.875) | 0.417 (0.133-0.714) | 0.250 (0.000-0.500) | 0.000 (0.000-0.000) |
| **Specificity (95% CI)** | 1.000 (1.000-1.000) | 1.000 (1.000-1.000) | 1.000 (1.000-1.000) | 1.000 (1.000-1.000) |
| **PPV (95% CI)** | 1.000 (1.000-1.000) | 1.000 (1.000-1.000) | 1.000 (1.000-1.000) | NA |
| **NPV (95% CI)** | 0.844 (0.706-0.967) | 0.794 (0.647-0.917) | 0.750 (0.600-0.886) | 0.692 (0.538-0.821) |
| **Accuracy (95% CI)** | 0.872 (0.769-0.974) | 0.821 (0.692-0.923) | 0.769 (0.641-0.897) | 0.692 (0.538-0.821) |
| Abbreviations: AUC=area under curve, CI=confidence interval, NA=not available, NPV=negative predictive value, LR=logistic regression, RF=random forest, ROC=receiver operating characteristic, PPV=positive predictive value, SVM=support vector machine, XGBoost=Extreme Gradient Boosting. | | | | |

## eFigure 10.4. SHapley Additive exPlanations (SHAP) summary plot for RF and XGBoost models (only top 20 predictors were presented)

| <RF model> | <XGBoost model> |
| --- | --- |
| 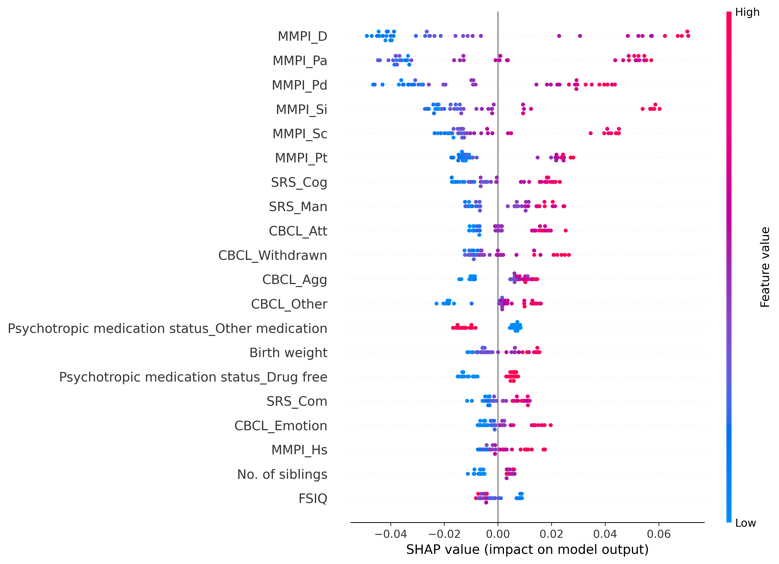 | 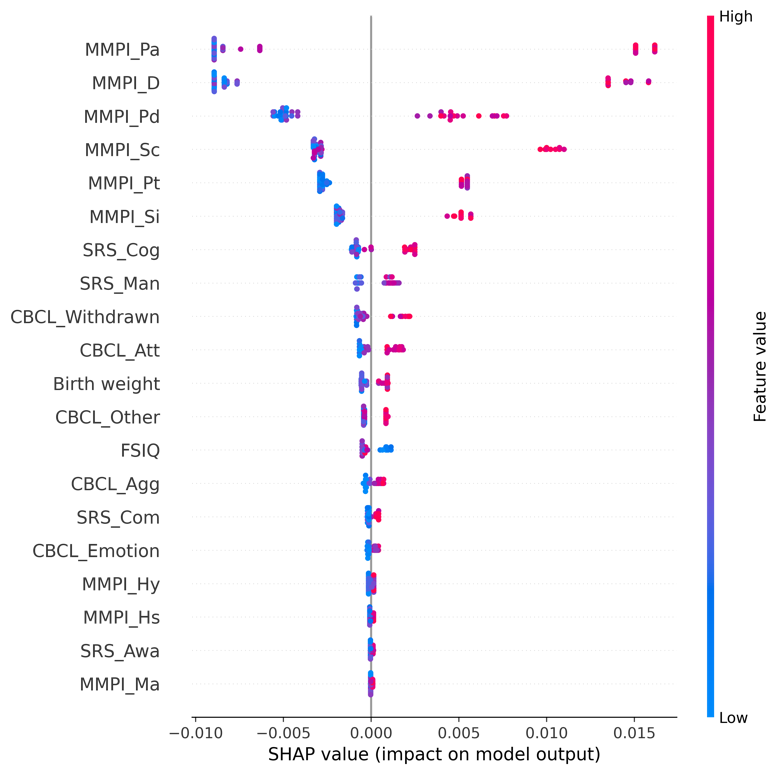 |

## eFigure 10.5. Receiver operating characteristic curves (ROC) for the training and test set

| <Training set> | <Test set> |
| --- | --- |
| 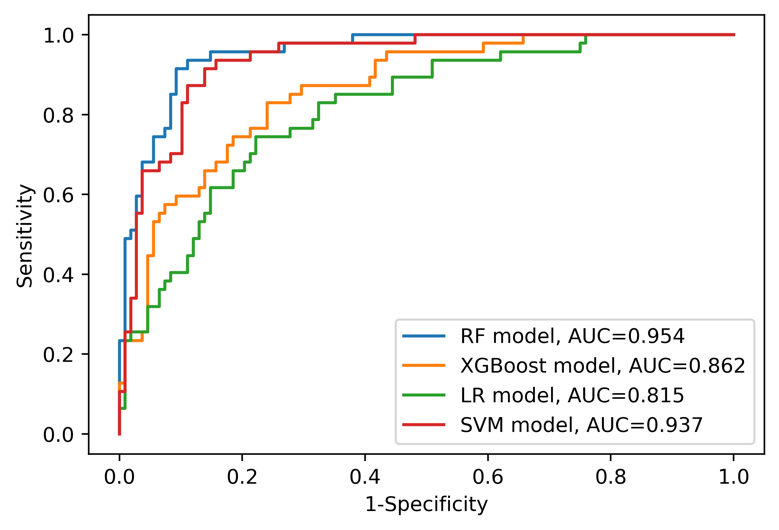 | 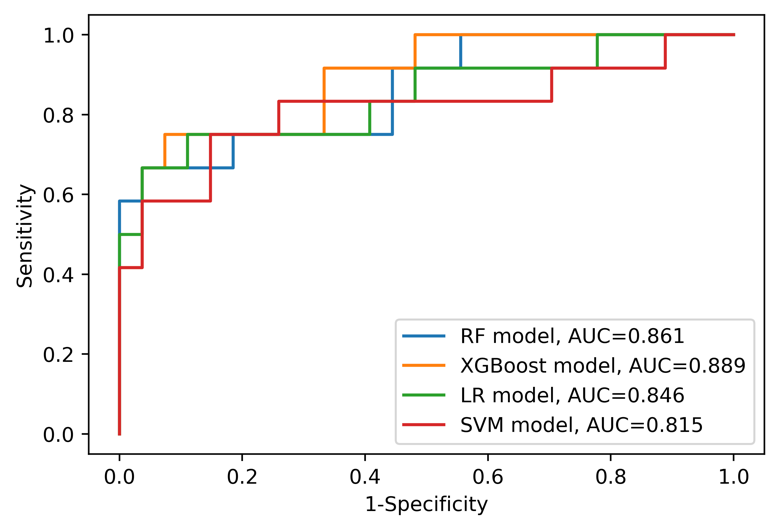 |

Abbreviations: AUC=area under curve, LR=logistic regression, RF=random forest, SVM=support vector machine, XGBoost=Extreme Gradient Boosting.

# **eAppendix 11. Model information and detailed results (Sample: CBCL 1.5-5 sample [n=194], Outcome: Parent-child dysfunctional interaction)**

## eTable 11.1. Model features (Variables are on the second columns)

| **Caregiver variables** | - Assistant caregiver status [Yes/No]  - Current working status [Yes/No]  - Minnesota Multiphasic Personality Inventory (MMPI) Hypochondriasis (T-score)  - MMPI Depression (T-score)  - MMPI Hysteria (T-score)  - MMPI Psychopathy (T-score)  - MMPI Masculinity/Femininity (T-score)  - MMPI Paranoia (T-score)  - MMPI Psychasthenia (T-score)  - MMPI Schizophrenia (T-score)  - MMPI Hypomania (T-score)  - MMPI Social Introversion (T-score)  - The order of birth  - Number of children |
| --- | --- |
| **ASD patient variables** | - Age  - Sex [Male/Female]  - Family history of mental disorder [Yes/No]  - History of major disease [Yes/No]  - Psychotropic medication status [Drug free/Monotherapy of antipsychotics/Combined therapy of antipsychotics/Other psychotic medication]  - Gestational age  - Birth weight  - Mode of delivery [Vaginal delivery/Caesarean section]  - Existence of another child with mental disorder [Yes/No] [Yes/No]  - Full-Scale Intelligence Quotient (FSIQ)  - Social responsiveness scale (SRS) Social awareness (T-score)  - SRS Social cognition (T-score)  - SRS Social communication (T-score)  - SRS Social motivation (T-score)  - SRS Autistic mannerisms (T-score)  - Child Behavior Checklist (CBCL) Emotionally reactivity (T-score)  - CBCL Anxious/depressed (T-score)  - CBCL Somatic complaints (T-score)  - CBCL Withdrawn/depressed (T-score)  - CBCL Sleep problems (T-score)  - CBCL Attention problems (T-score)  - CBCL Aggressive behavior (T-score)  - CBCL Other problems (T-score) |

## eTable 11.2. Performance of each model on the training set

|  | **RF model** | **XGBoost model** | **LR model** | **SVM model** |
| --- | --- | --- | --- | --- |
| **ROC AUC (95% CI)** | 0.981 (0.962-0.994) | 0.858 (0.789-0.919) | 0.820 (0.746-0.888) | 0.789 (0.711-0.863) |
| **Sensitivity (95% CI)** | 0.870 (0.773-0.952) | 0.389 (0.256-0.523) | 0.000 (0.000-0.000) | 0.000 (0.000-0.000) |
| **Specificity (95% CI)** | 0.980 (0.949-1.000) | 0.960 (0.918-0.991) | 1.000 (1.000-1.000) | 1.000 (1.000-1.000) |
| **PPV (95% CI)** | 0.959 (0.895-1.000) | 0.840 (0.684-0.963) | NA | NA |
| **NPV (95% CI)** | 0.934 (0.883-0.978) | 0.746 (0.669-0.819) | 0.652 (0.574-0.729) | 0.652 (0.574-0.729) |
| **Accuracy (95% CI)** | 0.942 (0.903-0.974) | 0.761 (0.690-0.826) | 0.652 (0.574-0.729) | 0.652 (0.574-0.729) |
| Abbreviations: AUC=area under curve, CI=confidence interval, NA=not available, NPV=negative predictive value, LR=logistic regression, RF=random forest, ROC=receiver operating characteristic, PPV=positive predictive value, SVM=support vector machine, XGBoost=Extreme Gradient Boosting. | | | | |

## eTable 11.3. Performance of each model on the test set

|  | **RF model** | **XGBoost model** | **LR model** | **SVM model** |
| --- | --- | --- | --- | --- |
| **ROC AUC (95% CI)** | 0.714 (0.533-0.869) | 0.720 (0.541-0.867) | 0.660 (0.475-0.826) | 0.686 (0.503-0.844) |
| **Sensitivity (95% CI)** | 0.429 (0.167-0.700) | 0.214 (0.000-0.455) | 0.000 (0.000-0.000) | 0.000 (0.000-0.000) |
| **Specificity (95% CI)** | 0.760 (0.583-0.920) | 0.960 (0.867-1.000) | 1.000 (1.000-1.000) | 1.000 (1.000-1.000) |
| **PPV (95% CI)** | 0.500 (0.200-0.786) | 0.750 (0.000-1.000) | NA | NA |
| **NPV (95% CI)** | 0.704 (0.520-0.867) | 0.686 (0.528-0.833) | 0.641 (0.487-0.795) | 0.641 (0.487-0.795) |
| **Accuracy (95% CI)** | 0.641 (0.487-0.795) | 0.692 (0.538-0.821) | 0.641 (0.487-0.795) | 0.641 (0.487-0.795) |
| Abbreviations: AUC=area under curve, CI=confidence interval, NA=not available, NPV=negative predictive value, LR=logistic regression, RF=random forest, ROC=receiver operating characteristic, PPV=positive predictive value, SVM=support vector machine, XGBoost=Extreme Gradient Boosting. | | | | |

## eFigure 11.4. SHapley Additive exPlanations (SHAP) summary plot for RF and XGBoost models (only top 20 predictors were presented)

| <RF model> | <XGBoost model> |
| --- | --- |
| 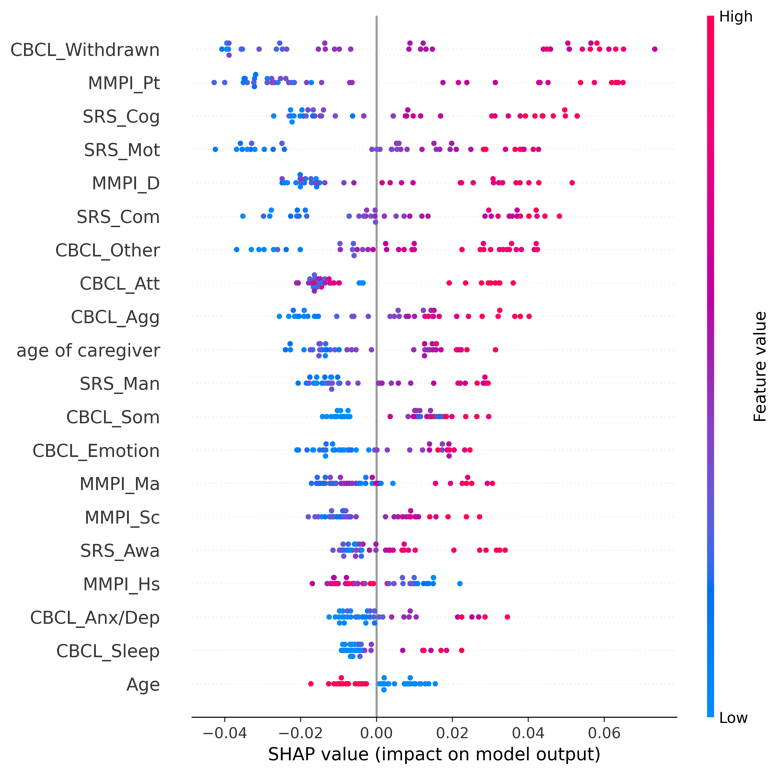 | 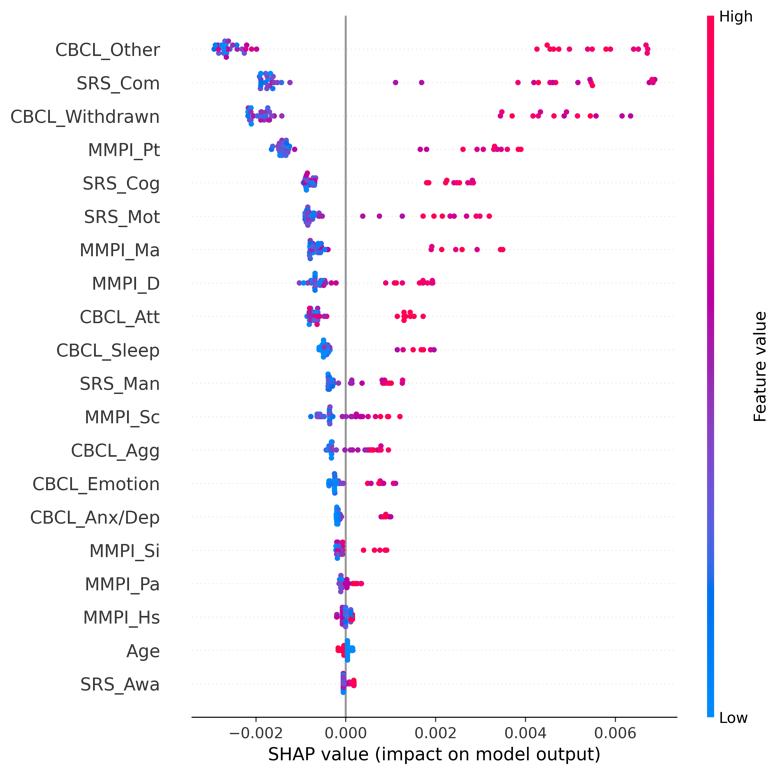 |

## eFigure 11.5. Receiver operating characteristic curves (ROC) for the training and test set

| <Training set> | <Test set> |
| --- | --- |
| 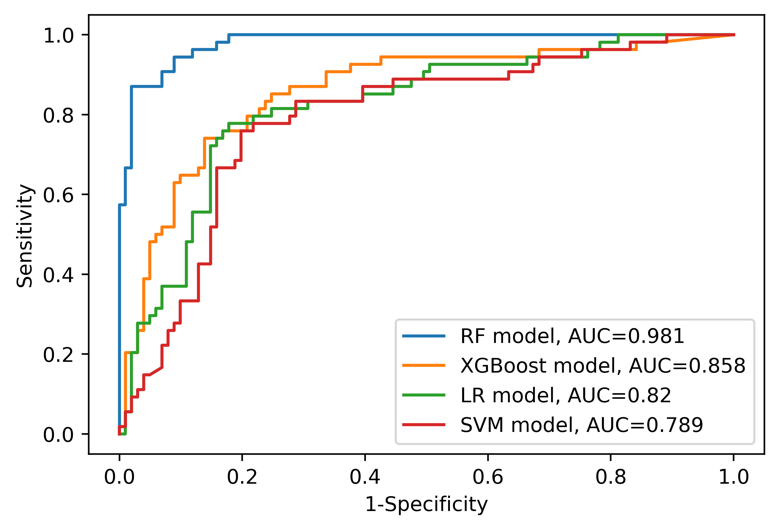 | 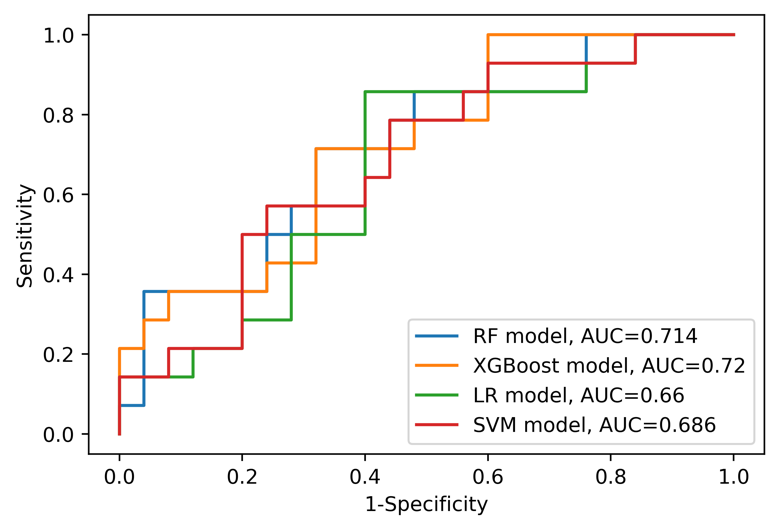 |

Abbreviations: AUC=area under curve, LR=logistic regression, RF=random forest, SVM=support vector machine, XGBoost=Extreme Gradient Boosting.

# **eAppendix 12. Model information and detailed results (Sample: CBCL 1.5-5 sample [n=194], Outcome: Difficult child)**

## eTable 12.1. Model features (Variables are on the second columns)

| **Caregiver variables** | - Assistant caregiver status [Yes/No]  - Current working status [Yes/No]  - Minnesota Multiphasic Personality Inventory (MMPI) Hypochondriasis (T-score)  - MMPI Depression (T-score)  - MMPI Hysteria (T-score)  - MMPI Psychopathy (T-score)  - MMPI Masculinity/Femininity (T-score)  - MMPI Paranoia (T-score)  - MMPI Psychasthenia (T-score)  - MMPI Schizophrenia (T-score)  - MMPI Hypomania (T-score)  - MMPI Social Introversion (T-score)  - The order of birth  - Number of children |
| --- | --- |
| **ASD patient variables** | - Age  - Sex [Male/Female]  - Family history of mental disorder [Yes/No]  - History of major disease [Yes/No]  - Psychotropic medication status [Drug free/Monotherapy of antipsychotics/Combined therapy of antipsychotics/Other psychotic medication]  - Gestational age  - Birth weight  - Mode of delivery [Vaginal delivery/Caesarean section]  - Existence of another child with mental disorder [Yes/No] [Yes/No]  - Full-Scale Intelligence Quotient (FSIQ)  - Social responsiveness scale (SRS) Social awareness (T-score)  - SRS Social cognition (T-score)  - SRS Social communication (T-score)  - SRS Social motivation (T-score)  - SRS Autistic mannerisms (T-score)  - Child Behavior Checklist (CBCL) Emotionally reactivity (T-score)  - CBCL Anxious/depressed (T-score)  - CBCL Somatic complaints (T-score)  - CBCL Withdrawn/depressed (T-score)  - CBCL Sleep problems (T-score)  - CBCL Attention problems (T-score)  - CBCL Aggressive behavior (T-score)  - CBCL Other problems (T-score) |

## eTable 12.2. Performance of each model on the training set

|  | **RF model** | **XGBoost model** | **LR model** | **SVM model** |
| --- | --- | --- | --- | --- |
| **ROC AUC (95% CI)** | 0.981 (0.962-0.994) | 0.957 (0.928-0.981) | 0.837 (0.769-0.899) | 0.784 (0.708-0.856) |
| **Sensitivity (95% CI)** | 0.800 (0.695-0.897) | 0.717 (0.600-0.828) | 0.533 (0.406-0.661) | 0.533 (0.404-0.661) |
| **Specificity (95% CI)** | 0.968 (0.928-1.000) | 0.947 (0.897-0.989) | 0.947 (0.899-0.989) | 0.853 (0.779-0.920) |
| **PPV (95% CI)** | 0.941 (0.867-1.000) | 0.896 (0.800-0.977) | 0.865 (0.743-0.970) | 0.696 (0.556-0.828) |
| **NPV (95% CI)** | 0.885 (0.820-0.943) | 0.841 (0.769-0.907) | 0.763 (0.683-0.838) | 0.743 (0.658-0.823) |
| **Accuracy (95% CI)** | 0.903 (0.852-0.948) | 0.858 (0.800-0.910) | 0.787 (0.723-0.852) | 0.729 (0.658-0.800) |
| Abbreviations: AUC=area under curve, CI=confidence interval, NA=not available, NPV=negative predictive value, LR=logistic regression, RF=random forest, ROC=receiver operating characteristic, PPV=positive predictive value, SVM=support vector machine, XGBoost=Extreme Gradient Boosting. | | | | |

## eTable 12.3. Performance of each model on the test set

|  | **RF model** | **XGBoost model** | **LR model** | **SVM model** |
| --- | --- | --- | --- | --- |
| **ROC AUC (95% CI)** | 0.897 (0.765-0.988) | 0.925 (0.817-0.992) | 0.811 (0.653-0.934) | 0.853 (0.704-0.961) |
| **Sensitivity (95% CI)** | 0.667 (0.412-0.900) | 0.733 (0.500-0.933) | 0.333 (0.100-0.579) | 0.400 (0.154-0.647) |
| **Specificity (95% CI)** | 0.958 (0.864-1.000) | 0.917 (0.789-1.000) | 1.000 (1.000-1.000) | 0.917 (0.789-1.000) |
| **PPV (95% CI)** | 0.909 (0.700-1.000) | 0.846 (0.615-1.000) | 1.000 (1.000-1.000) | 0.750 (0.400-1.000) |
| **NPV (95% CI)** | 0.821 (0.667-0.958) | 0.846 (0.692-0.964) | 0.706 (0.545-0.853) | 0.710 (0.543-0.862) |
| **Accuracy (95% CI)** | 0.846 (0.718-0.949) | 0.846 (0.718-0.949) | 0.744 (0.615-0.872) | 0.718 (0.564-0.846) |
| Abbreviations: AUC=area under curve, CI=confidence interval, NA=not available, NPV=negative predictive value, LR=logistic regression, RF=random forest, ROC=receiver operating characteristic, PPV=positive predictive value, SVM=support vector machine, XGBoost=Extreme Gradient Boosting. | | | | |

## eFigure 12.4. SHapley Additive exPlanations (SHAP) summary plot for RF and XGBoost models (only top 20 predictors were presented)

| <RF model> | <XGBoost model> |
| --- | --- |
| 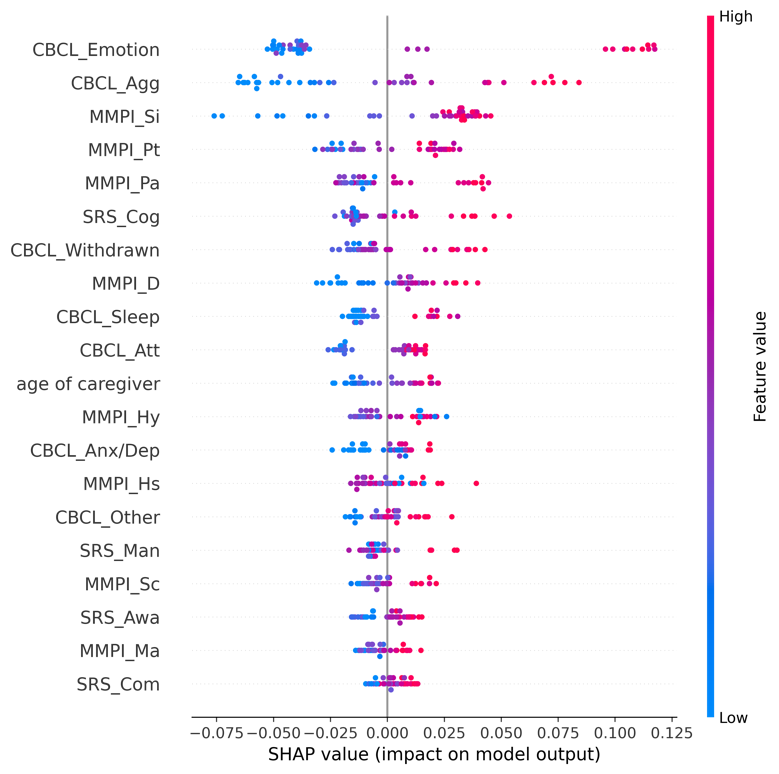 | 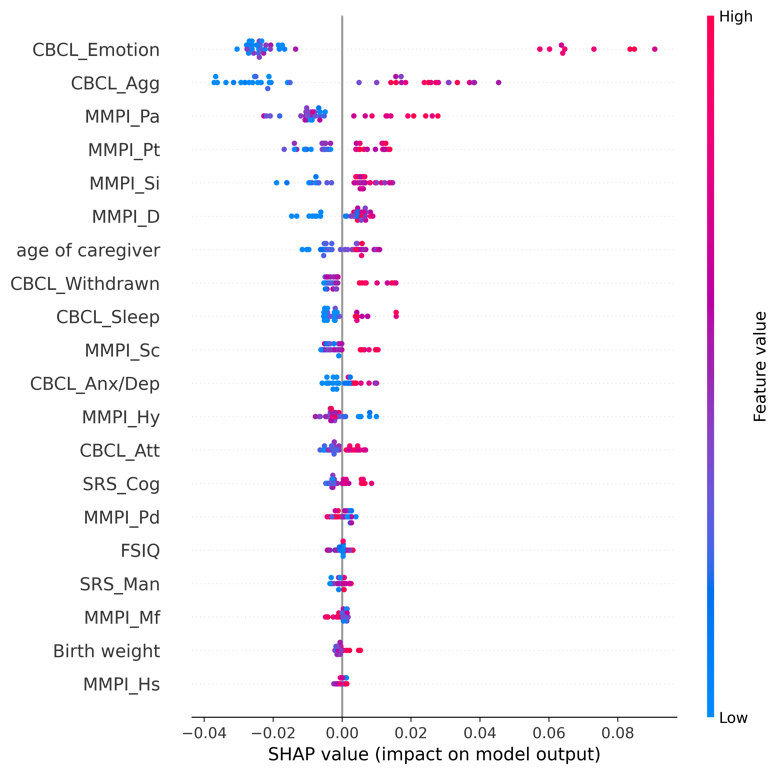 |

## eFigure 12.5. Receiver operating characteristic curves (ROC) for the training and test set

| <Training set> | <Test set> |
| --- | --- |
| 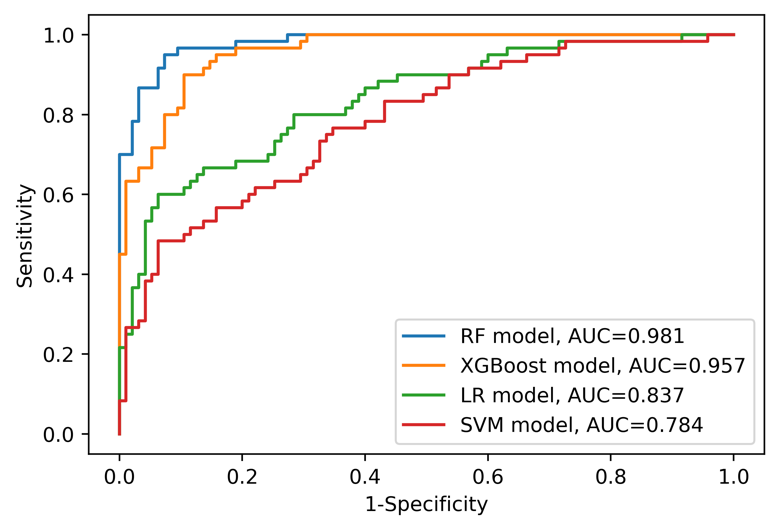 | 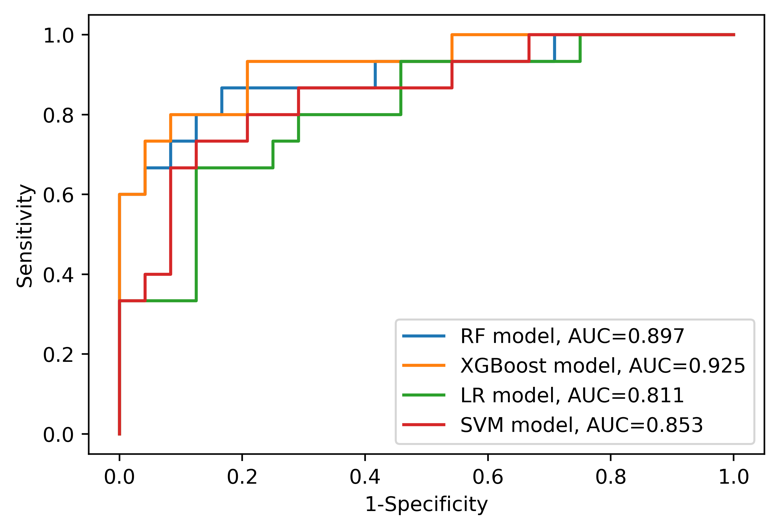 |

Abbreviations: AUC=area under curve, LR=logistic regression, RF=random forest, SVM=support vector machine, XGBoost=Extreme Gradient Boosting.

# **eAppendix 13. Model information and detailed results (Sample: CBCL 1.5-5 sample [n=194], Outcome: Total parenting stress)**

## eTable 13.1. Model features (Variables are on the second columns)

| **Caregiver variables** | - Assistant caregiver status [Yes/No]  - Current working status [Yes/No]  - Minnesota Multiphasic Personality Inventory (MMPI) Hypochondriasis (T-score)  - MMPI Depression (T-score)  - MMPI Hysteria (T-score)  - MMPI Psychopathy (T-score)  - MMPI Masculinity/Femininity (T-score)  - MMPI Paranoia (T-score)  - MMPI Psychasthenia (T-score)  - MMPI Schizophrenia (T-score)  - MMPI Hypomania (T-score)  - MMPI Social Introversion (T-score)  - The order of birth  - Number of children |
| --- | --- |
| **ASD patient variables** | - Age  - Sex [Male/Female]  - Family history of mental disorder [Yes/No]  - History of major disease [Yes/No]  - Psychotropic medication status [Drug free/Monotherapy of antipsychotics/Combined therapy of antipsychotics/Other psychotic medication]  - Gestational age  - Birth weight  - Mode of delivery [Vaginal delivery/Caesarean section]  - Existence of another child with mental disorder [Yes/No] [Yes/No]  - Full-Scale Intelligence Quotient (FSIQ)  - Social responsiveness scale (SRS) Social awareness (T-score)  - SRS Social cognition (T-score)  - SRS Social communication (T-score)  - SRS Social motivation (T-score)  - SRS Autistic mannerisms (T-score)  - Child Behavior Checklist (CBCL) Emotionally reactivity (T-score)  - CBCL Anxious/depressed (T-score)  - CBCL Somatic complaints (T-score)  - CBCL Withdrawn/depressed (T-score)  - CBCL Sleep problems (T-score)  - CBCL Attention problems (T-score)  - CBCL Aggressive behavior (T-score)  - CBCL Other problems (T-score) |

## eTable 13.2. Performance of each model on the training set

|  | **RF model** | **XGBoost model** | **LR model** | **SVM model** |
| --- | --- | --- | --- | --- |
| **ROC AUC (95% CI)** | 0.988 (0.972-0.998) | 0.944 (0.910-0.972) | 0.867 (0.806-0.920) | 0.863 (0.801-0.917) |
| **Sensitivity (95% CI)** | 0.905 (0.825-0.970) | 0.825 (0.726-0.915) | 0.524 (0.400-0.648) | 0.000 (0.000-0.000) |
| **Specificity (95% CI)** | 0.978 (0.944-1.000) | 0.870 (0.798-0.933) | 0.924 (0.866-0.971) | 1.000 (1.000-1.000) |
| **PPV (95% CI)** | 0.966 (0.912-1.000) | 0.812 (0.712-0.902) | 0.825 (0.700-0.933) | NA |
| **NPV (95% CI)** | 0.938 (0.883-0.980) | 0.879 (0.806-0.943) | 0.739 (0.655-0.817) | 0.594 (0.516-0.671) |
| **Accuracy (95% CI)** | 0.948 (0.910-0.981) | 0.852 (0.794-0.903) | 0.761 (0.690-0.826) | 0.594 (0.516-0.671) |
| Abbreviations: AUC=area under curve, CI=confidence interval, NA=not available, NPV=negative predictive value, LR=logistic regression, RF=random forest, ROC=receiver operating characteristic, PPV=positive predictive value, SVM=support vector machine, XGBoost=Extreme Gradient Boosting. | | | | |

## eTable 13.3. Performance of each model on the test set

|  | **RF model** | **XGBoost model** | **LR model** | **SVM model** |
| --- | --- | --- | --- | --- |
| **ROC AUC (95% CI)** | 0.845 (0.703-0.954) | 0.810 (0.648-0.936) | 0.788 (0.630-0.917) | 0.815 (0.667-0.935) |
| **Sensitivity (95% CI)** | 0.625 (0.375-0.857) | 0.625 (0.375-0.857) | 0.375 (0.143-0.625) | 0.000 (0.000-0.000) |
| **Specificity (95% CI)** | 0.870 (0.714-1.000) | 0.826 (0.654-0.960) | 0.957 (0.857-1.000) | 1.000 (1.000-1.000) |
| **PPV (95% CI)** | 0.769 (0.500-1.000) | 0.714 (0.455-0.933) | 0.857 (0.500-1.000) | NA |
| **NPV (95% CI)** | 0.769 (0.594-0.920) | 0.760 (0.577-0.917) | 0.688 (0.516-0.844) | 0.590 (0.436-0.744) |
| **Accuracy (95% CI)** | 0.769 (0.641-0.897) | 0.744 (0.590-0.872) | 0.718 (0.564-0.846) | 0.590 (0.436-0.744) |
| Abbreviations: AUC=area under curve, CI=confidence interval, NA=not available, NPV=negative predictive value, LR=logistic regression, RF=random forest, ROC=receiver operating characteristic, PPV=positive predictive value, SVM=support vector machine, XGBoost=Extreme Gradient Boosting. | | | | |

## eFigure 13.4. SHapley Additive exPlanations (SHAP) summary plot for RF and XGBoost models (only top 20 predictors were presented)

| <RF model> | <XGBoost model> |
| --- | --- |
| 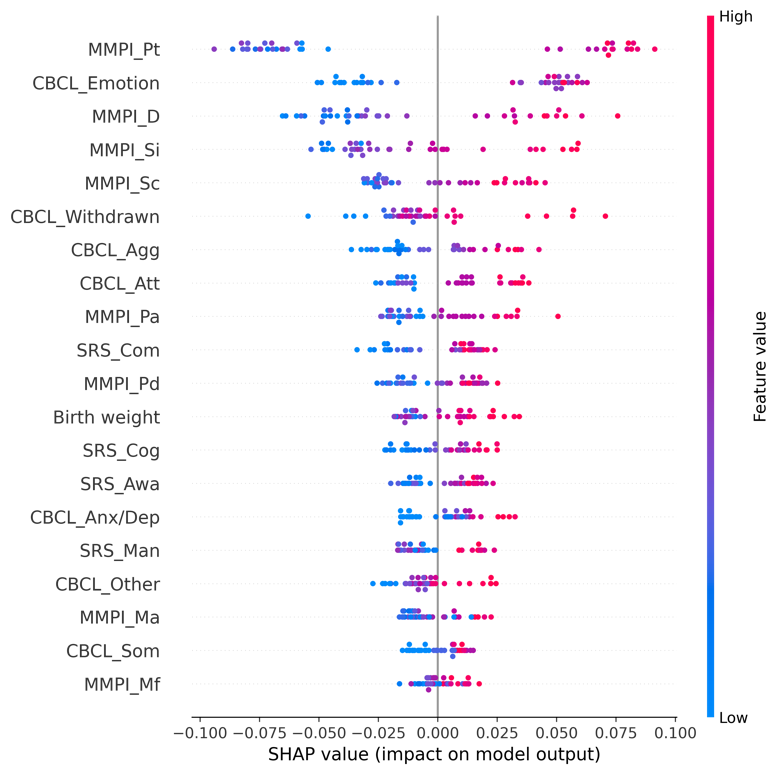 | 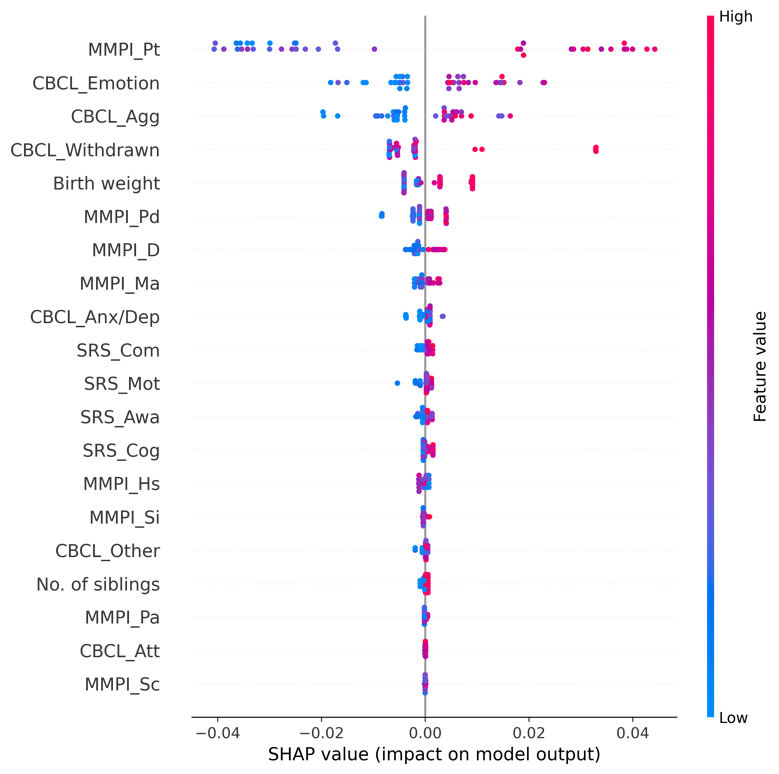 |

## eFigure 13.5. Receiver operating characteristic curves (ROC) for the training and test set

| <Training set> | <Test set> |
| --- | --- |
| 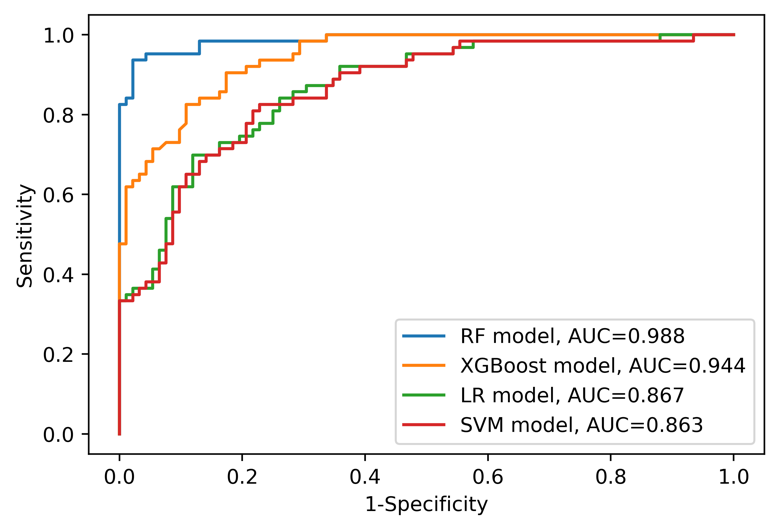 | 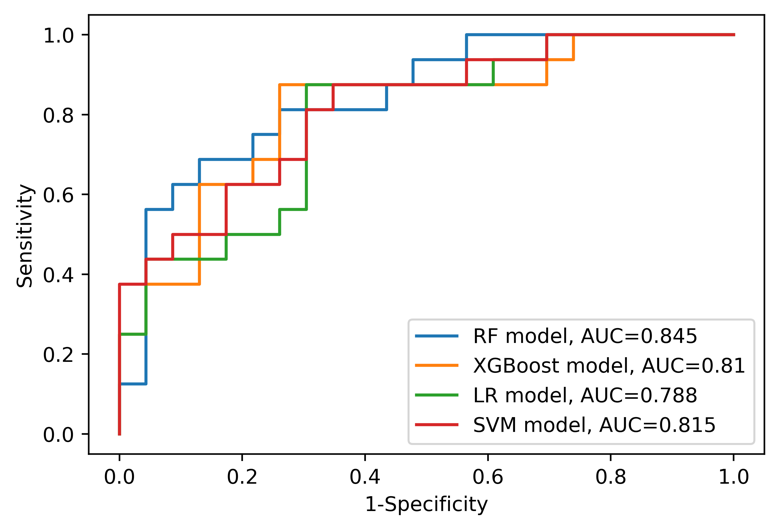 |

Abbreviations: AUC=area under curve, LR=logistic regression, RF=random forest, SVM=support vector machine, XGBoost=Extreme Gradient Boosting.

# **eAppendix 14. Model information and detailed results (Sample: CBCL 6-18 sample [n=302], Outcome: Parental distress)**

## eTable 14.1. Model features (Variables are on the second columns)

| **Caregiver variables** | - Assistant caregiver status [Yes/No]  - Current working status [Yes/No]  - Minnesota Multiphasic Personality Inventory (MMPI) Hypochondriasis (T-score)  - MMPI Depression (T-score)  - MMPI Hysteria (T-score)  - MMPI Psychopathy (T-score)  - MMPI Masculinity/Femininity (T-score)  - MMPI Paranoia (T-score)  - MMPI Psychasthenia (T-score)  - MMPI Schizophrenia (T-score)  - MMPI Hypomania (T-score)  - MMPI Social Introversion (T-score)  - The order of birth  - Number of children |
| --- | --- |
| **ASD patient variables** | - Age  - Sex [Male/Female]  - Family history of mental disorder [Yes/No]  - History of major disease [Yes/No]  - Psychotropic medication status [Drug free/Monotherapy of antipsychotics/Combined therapy of antipsychotics/Other psychotic medication]  - Gestational age  - Birth weight  - Mode of delivery [Vaginal delivery/Caesarean section]  - Existence of another child with mental disorder [Yes/No] [Yes/No]  - Full-Scale Intelligence Quotient (FSIQ)  - Social responsiveness scale (SRS) Social awareness (T-score)  - SRS Social cognition (T-score)  - SRS Social communication (T-score)  - SRS Social motivation (T-score)  - SRS Autistic mannerisms (T-score)  - Child Behavior Checklist (CBCL) Anxious/depressed (T-score)  - CBCL Withdrawn/depressed (T-score)  - CBCL Somatic complaints (T-score)  - CBCL Social problems (T-score)  - CBCL Thought problems (T-score)  - CBCL Attention problems (T-score)  - CBCL Rule-breaking behavior (T-score)  - CBCL Aggressive behavior (T-score)  - CBCL Other problems (T-score) |

## eTable 14.2. Performance of each model on the training set

|  | **RF model** | **XGBoost model** | **LR model** | **SVM model** |
| --- | --- | --- | --- | --- |
| **ROC AUC (95% CI)** | 0.964 (0.941-0.982) | 0.905 (0.866-0.939) | 0.884 (0.833-0.929) | 0.889 (0.836-0.934) |
| **Sensitivity (95% CI)** | 0.618 (0.500-0.731) | 0.618 (0.500-0.731) | 0.603 (0.483-0.719) | 0.574 (0.453-0.694) |
| **Specificity (95% CI)** | 0.988 (0.970-1.000) | 0.902 (0.856-0.944) | 0.948 (0.912-0.978) | 0.965 (0.936-0.989) |
| **PPV (95% CI)** | 0.955 (0.885-1.000) | 0.712 (0.593-0.824) | 0.820 (0.706-0.921) | 0.867 (0.759-0.957) |
| **NPV (95% CI)** | 0.868 (0.818-0.914) | 0.857 (0.803-0.906) | 0.859 (0.806-0.907) | 0.852 (0.801-0.901) |
| **Accuracy (95% CI)** | 0.884 (0.842-0.921) | 0.822 (0.772-0.867) | 0.851 (0.805-0.892) | 0.855 (0.809-0.896) |
| Abbreviations: AUC=area under curve, CI=confidence interval, NA=not available, NPV=negative predictive value, LR=logistic regression, RF=random forest, ROC=receiver operating characteristic, PPV=positive predictive value, SVM=support vector machine, XGBoost=Extreme Gradient Boosting. | | | | |

## eTable 14.3. Performance of each model on the test set

|  | **RF model** | **XGBoost model** | **LR model** | **SVM model** |
| --- | --- | --- | --- | --- |
| **ROC AUC (95% CI)** | 0.803 (0.661-0.924) | 0.709 (0.544-0.860) | 0.787 (0.659-0.895) | 0.770 (0.639-0.881) |
| **Sensitivity (95% CI)** | 0.412 (0.182-0.667) | 0.529 (0.286-0.778) | 0.412 (0.182-0.650) | 0.412 (0.182-0.650) |
| **Specificity (95% CI)** | 0.932 (0.848-1.000) | 0.886 (0.786-0.975) | 0.818 (0.698-0.927) | 0.841 (0.725-0.939) |
| **PPV (95% CI)** | 0.700 (0.400-1.000) | 0.643 (0.375-0.889) | 0.467 (0.211-0.727) | 0.500 (0.231-0.769) |
| **NPV (95% CI)** | 0.804 (0.688-0.904) | 0.830 (0.711-0.933) | 0.783 (0.659-0.891) | 0.787 (0.667-0.894) |
| **Accuracy (95% CI)** | 0.787 (0.672-0.885) | 0.787 (0.672-0.885) | 0.705 (0.590-0.820) | 0.721 (0.607-0.820) |
| Abbreviations: AUC=area under curve, CI=confidence interval, NA=not available, NPV=negative predictive value, LR=logistic regression, RF=random forest, ROC=receiver operating characteristic, PPV=positive predictive value, SVM=support vector machine, XGBoost=Extreme Gradient Boosting. | | | | |

## eFigure 14.4. SHapley Additive exPlanations (SHAP) summary plot for RF and XGBoost models (only top 20 predictors were presented)

| <RF model> | <XGBoost model> |
| --- | --- |
| 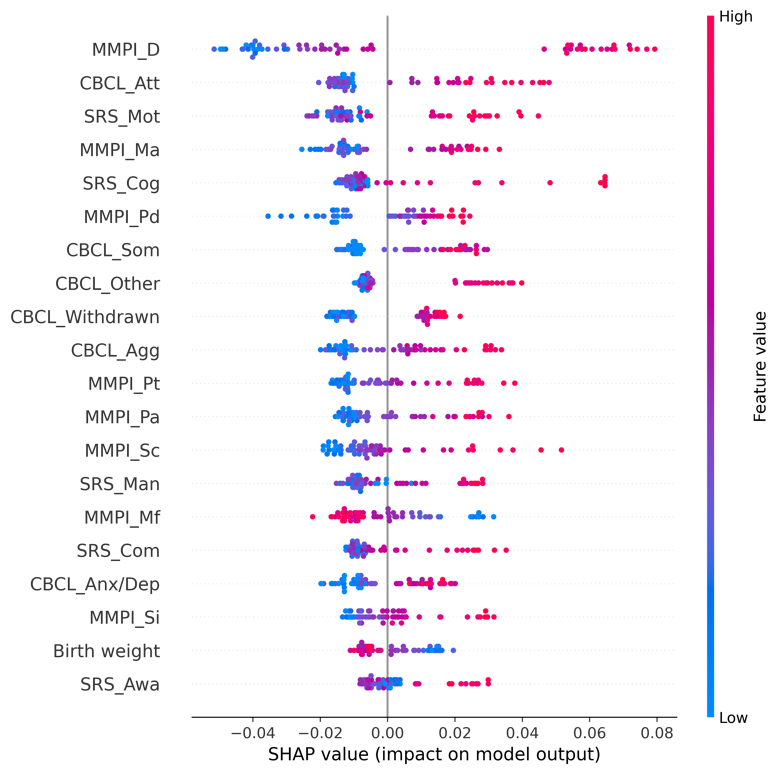 | 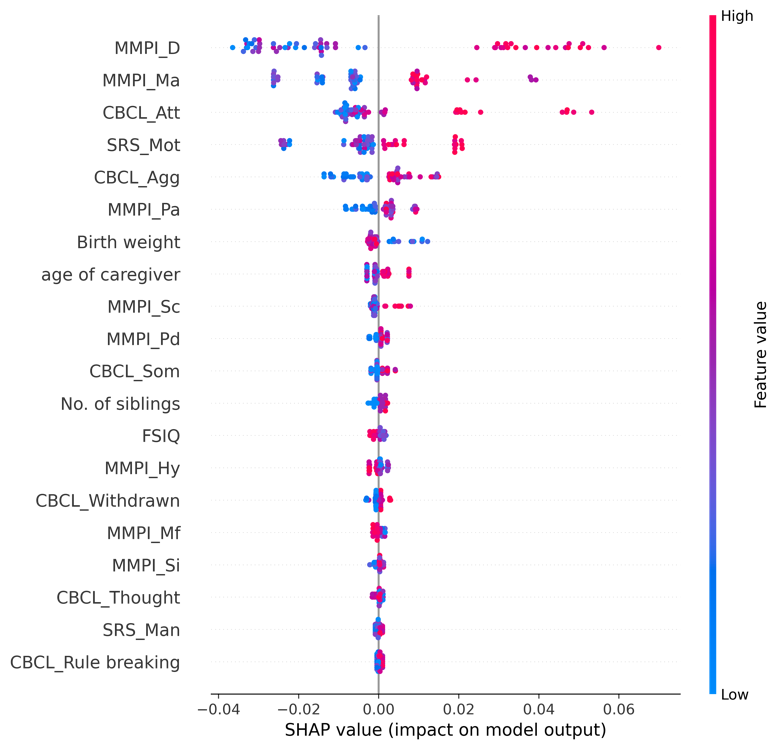 |

## eFigure 14.5. Receiver operating characteristic curves (ROC) for the training and test set

| <Training set> | <Test set> |
| --- | --- |
| 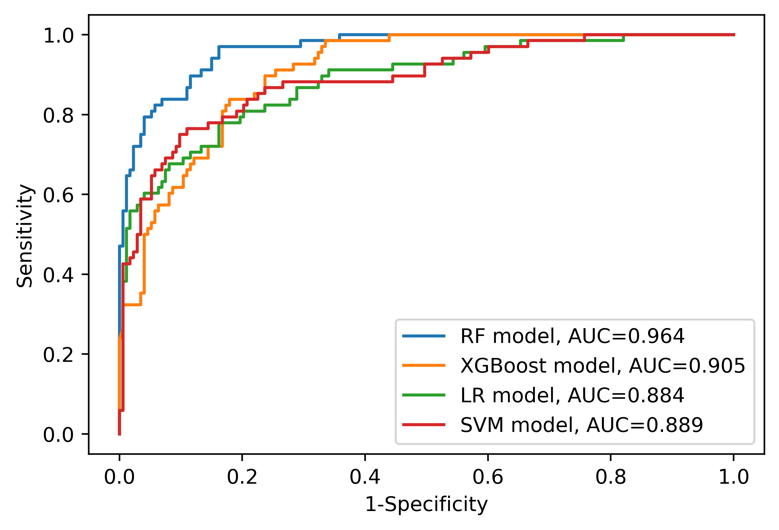 | 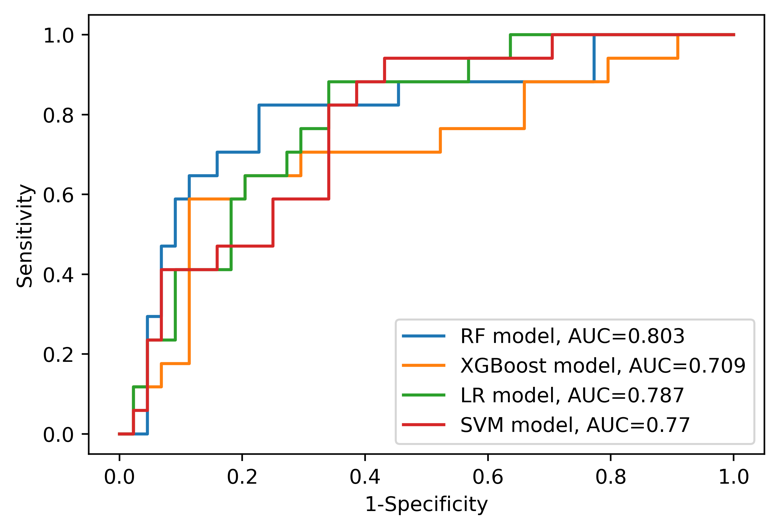 |

Abbreviations: AUC=area under curve, LR=logistic regression, RF=random forest, SVM=support vector machine, XGBoost=Extreme Gradient Boosting.

# **eAppendix 15. Model information and detailed results (Sample: CBCL 6-18 sample [n=302], Outcome: Parent-child dysfunctional interaction)**

## eTable 15.1. Model features (Variables are on the second columns)

| **Caregiver variables** | - Assistant caregiver status [Yes/No]  - Current working status [Yes/No]  - Minnesota Multiphasic Personality Inventory (MMPI) Hypochondriasis (T-score)  - MMPI Depression (T-score)  - MMPI Hysteria (T-score)  - MMPI Psychopathy (T-score)  - MMPI Masculinity/Femininity (T-score)  - MMPI Paranoia (T-score)  - MMPI Psychasthenia (T-score)  - MMPI Schizophrenia (T-score)  - MMPI Hypomania (T-score)  - MMPI Social Introversion (T-score)  - The order of birth  - Number of children |
| --- | --- |
| **ASD patient variables** | - Age  - Sex [Male/Female]  - Family history of mental disorder [Yes/No]  - History of major disease [Yes/No]  - Psychotropic medication status [Drug free/Monotherapy of antipsychotics/Combined therapy of antipsychotics/Other psychotic medication]  - Gestational age  - Birth weight  - Mode of delivery [Vaginal delivery/Caesarean section]  - Existence of another child with mental disorder [Yes/No] [Yes/No]  - Full-Scale Intelligence Quotient (FSIQ)  - Social responsiveness scale (SRS) Social awareness (T-score)  - SRS Social cognition (T-score)  - SRS Social communication (T-score)  - SRS Social motivation (T-score)  - SRS Autistic mannerisms (T-score)  - Child Behavior Checklist (CBCL) Anxious/depressed (T-score)  - CBCL Withdrawn/depressed (T-score)  - CBCL Somatic complaints (T-score)  - CBCL Social problems (T-score)  - CBCL Thought problems (T-score)  - CBCL Attention problems (T-score)  - CBCL Rule-breaking behavior (T-score)  - CBCL Aggressive behavior (T-score)  - CBCL Other problems (T-score) |

## eTable 15.2. Performance of each model on the training set

|  | **RF model** | **XGBoost model** | **LR model** | **SVM model** |
| --- | --- | --- | --- | --- |
| **ROC AUC (95% CI)** | 0.980 (0.966-0.991) | 0.931 (0.899-0.958) | 0.812 (0.758-0.863) | 0.806 (0.752-0.859) |
| **Sensitivity (95% CI)** | 0.912 (0.857-0.960) | 0.841 (0.771-0.906) | 0.673 (0.586-0.758) | 0.681 (0.596-0.766) |
| **Specificity (95% CI)** | 0.938 (0.893-0.976) | 0.875 (0.813-0.930) | 0.789 (0.716-0.856) | 0.750 (0.672-0.824) |
| **PPV (95% CI)** | 0.928 (0.877-0.973) | 0.856 (0.787-0.918) | 0.738 (0.653-0.821) | 0.706 (0.623-0.789) |
| **NPV (95% CI)** | 0.923 (0.875-0.966) | 0.862 (0.800-0.919) | 0.732 (0.655-0.804) | 0.727 (0.649-0.803) |
| **Accuracy (95% CI)** | 0.925 (0.892-0.959) | 0.859 (0.813-0.900) | 0.734 (0.676-0.788) | 0.718 (0.660-0.776) |
| Abbreviations: AUC=area under curve, CI=confidence interval, NA=not available, NPV=negative predictive value, LR=logistic regression, RF=random forest, ROC=receiver operating characteristic, PPV=positive predictive value, SVM=support vector machine, XGBoost=Extreme Gradient Boosting. | | | | |

## eTable 15.3. Performance of each model on the test set

|  | **RF model** | **XGBoost model** | **LR model** | **SVM model** |
| --- | --- | --- | --- | --- |
| **ROC AUC (95% CI)** | 0.745 (0.611-0.860) | 0.750 (0.620-0.864) | 0.720 (0.580-0.841) | 0.726 (0.586-0.847) |
| **Sensitivity (95% CI)** | 0.679 (0.500-0.846) | 0.679 (0.500-0.848) | 0.571 (0.385-0.750) | 0.643 (0.458-0.816) |
| **Specificity (95% CI)** | 0.606 (0.438-0.769) | 0.606 (0.438-0.769) | 0.667 (0.500-0.824) | 0.697 (0.533-0.848) |
| **PPV (95% CI)** | 0.594 (0.414-0.759) | 0.594 (0.419-0.760) | 0.593 (0.400-0.778) | 0.643 (0.458-0.815) |
| **NPV (95% CI)** | 0.690 (0.517-0.853) | 0.690 (0.517-0.852) | 0.647 (0.483-0.800) | 0.697 (0.531-0.848) |
| **Accuracy (95% CI)** | 0.639 (0.525-0.754) | 0.639 (0.525-0.754) | 0.623 (0.492-0.738) | 0.672 (0.557-0.787) |
| Abbreviations: AUC=area under curve, CI=confidence interval, NA=not available, NPV=negative predictive value, LR=logistic regression, RF=random forest, ROC=receiver operating characteristic, PPV=positive predictive value, SVM=support vector machine, XGBoost=Extreme Gradient Boosting. | | | | |

## eFigure 15.4. SHapley Additive exPlanations (SHAP) summary plot for RF and XGBoost models (only top 20 predictors were presented)

| <RF model> | <XGBoost model> |
| --- | --- |
| 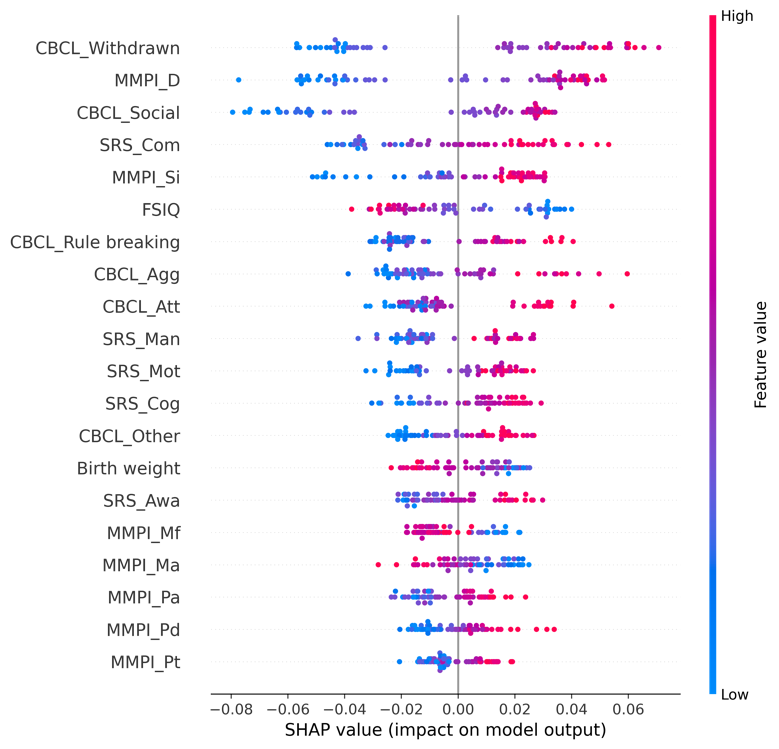 | 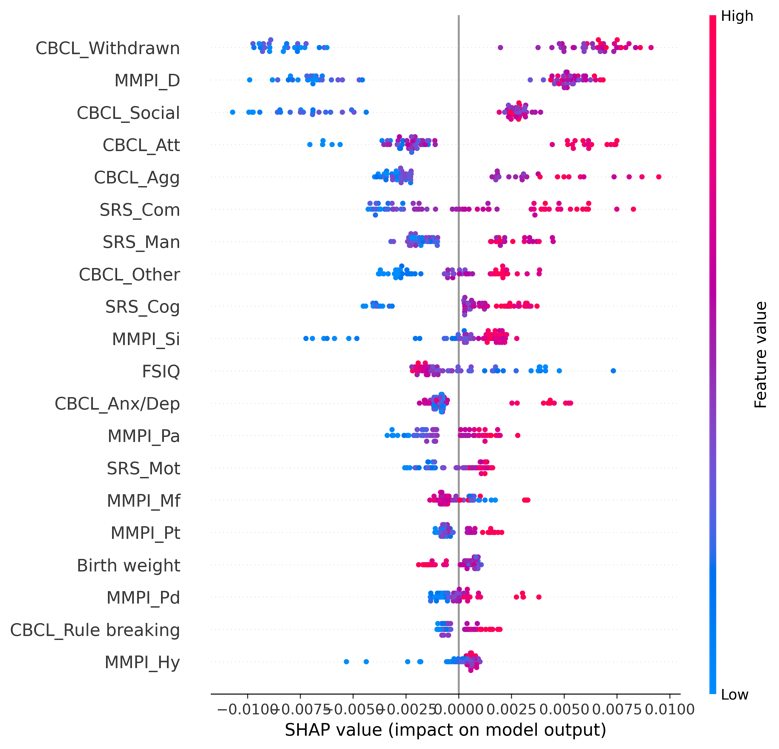 |

## eFigure 15.5. Receiver operating characteristic curves (ROC) for the training and test set

| <Training set> | <Test set> |
| --- | --- |
| 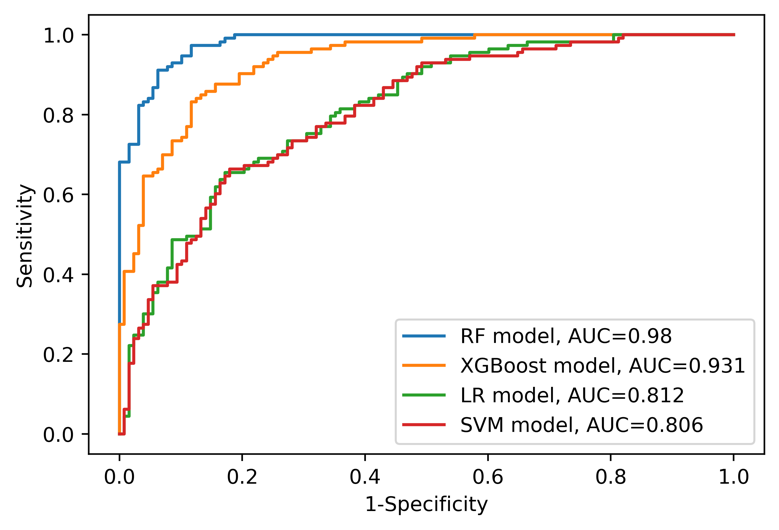 | 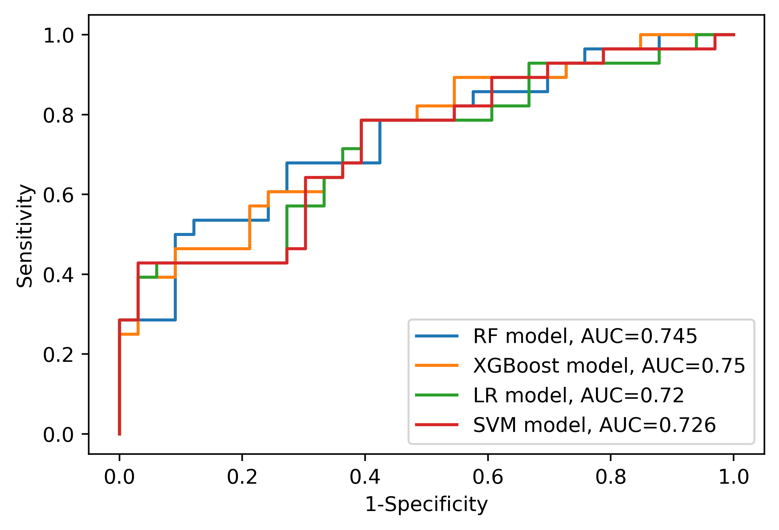 |

Abbreviations: AUC=area under curve, LR=logistic regression, RF=random forest, SVM=support vector machine, XGBoost=Extreme Gradient Boosting.

# **eAppendix 16. Model information and detailed results (Sample: CBCL 6-18 sample [n=302], Outcome: Difficult child)**

## eTable 16.1. Model features (Variables are on the second columns)

| **Caregiver variables** | - Assistant caregiver status [Yes/No]  - Current working status [Yes/No]  - Minnesota Multiphasic Personality Inventory (MMPI) Hypochondriasis (T-score)  - MMPI Depression (T-score)  - MMPI Hysteria (T-score)  - MMPI Psychopathy (T-score)  - MMPI Masculinity/Femininity (T-score)  - MMPI Paranoia (T-score)  - MMPI Psychasthenia (T-score)  - MMPI Schizophrenia (T-score)  - MMPI Hypomania (T-score)  - MMPI Social Introversion (T-score)  - The order of birth  - Number of children |
| --- | --- |
| **ASD patient variables** | - Age  - Sex [Male/Female]  - Family history of mental disorder [Yes/No]  - History of major disease [Yes/No]  - Psychotropic medication status [Drug free/Monotherapy of antipsychotics/Combined therapy of antipsychotics/Other psychotic medication]  - Gestational age  - Birth weight  - Mode of delivery [Vaginal delivery/Caesarean section]  - Existence of another child with mental disorder [Yes/No] [Yes/No]  - Full-Scale Intelligence Quotient (FSIQ)  - Social responsiveness scale (SRS) Social awareness (T-score)  - SRS Social cognition (T-score)  - SRS Social communication (T-score)  - SRS Social motivation (T-score)  - SRS Autistic mannerisms (T-score)  - Child Behavior Checklist (CBCL) Anxious/depressed (T-score)  - CBCL Withdrawn/depressed (T-score)  - CBCL Somatic complaints (T-score)  - CBCL Social problems (T-score)  - CBCL Thought problems (T-score)  - CBCL Attention problems (T-score)  - CBCL Rule-breaking behavior (T-score)  - CBCL Aggressive behavior (T-score)  - CBCL Other problems (T-score) |

## eTable 16.2. Performance of each model on the training set

|  | **RF model** | **XGBoost model** | **LR model** | **SVM model** |
| --- | --- | --- | --- | --- |
| **ROC AUC (95% CI)** | 0.992 (0.984-0.997) | 0.930 (0.897-0.958) | 0.883 (0.839-0.923) | 1.000 (1.000-1.000) |
| **Sensitivity (95% CI)** | 0.950 (0.908-0.984) | 0.858 (0.792-0.917) | 0.825 (0.752-0.891) | 1.000 (1.000-1.000) |
| **Specificity (95% CI)** | 0.909 (0.856-0.957) | 0.802 (0.729-0.870) | 0.802 (0.729-0.870) | 1.000 (1.000-1.000) |
| **PPV (95% CI)** | 0.912 (0.860-0.958) | 0.811 (0.739-0.877) | 0.805 (0.733-0.873) | 1.000 (1.000-1.000) |
| **NPV (95% CI)** | 0.948 (0.904-0.983) | 0.851 (0.782-0.914) | 0.822 (0.748-0.888) | 1.000 (1.000-1.000) |
| **Accuracy (95% CI)** | 0.929 (0.896-0.959) | 0.830 (0.780-0.876) | 0.813 (0.763-0.863) | 1.000 (1.000-1.000) |
| Abbreviations: AUC=area under curve, CI=confidence interval, NA=not available, NPV=negative predictive value, LR=logistic regression, RF=random forest, ROC=receiver operating characteristic, PPV=positive predictive value, SVM=support vector machine, XGBoost=Extreme Gradient Boosting. | | | | |

## eTable 16.3. Performance of each model on the test set

|  | **RF model** | **XGBoost model** | **LR model** | **SVM model** |
| --- | --- | --- | --- | --- |
| **ROC AUC (95% CI)** | 0.805 (0.683-0.912) | 0.814 (0.697-0.917) | 0.777 (0.649-0.891) | 0.726 (0.589-0.847) |
| **Sensitivity (95% CI)** | 0.867 (0.735-0.969) | 0.867 (0.733-0.969) | 0.867 (0.735-0.969) | 0.900 (0.783-1.000) |
| **Specificity (95% CI)** | 0.548 (0.370-0.719) | 0.645 (0.471-0.806) | 0.645 (0.469-0.806) | 0.484 (0.306-0.658) |
| **PPV (95% CI)** | 0.650 (0.500-0.794) | 0.703 (0.548-0.844) | 0.703 (0.548-0.842) | 0.628 (0.477-0.767) |
| **NPV (95% CI)** | 0.810 (0.625-0.957) | 0.833 (0.667-0.962) | 0.833 (0.667-0.962) | 0.833 (0.640-1.000) |
| **Accuracy (95% CI)** | 0.705 (0.590-0.820) | 0.754 (0.639-0.852) | 0.754 (0.639-0.852) | 0.689 (0.574-0.803) |
| Abbreviations: AUC=area under curve, CI=confidence interval, NA=not available, NPV=negative predictive value, LR=logistic regression, RF=random forest, ROC=receiver operating characteristic, PPV=positive predictive value, SVM=support vector machine, XGBoost=Extreme Gradient Boosting. | | | | |

## eFigure 16.4. SHapley Additive exPlanations (SHAP) summary plot for RF and XGBoost models (only top 20 predictors were presented)

| <RF model> | <XGBoost model> |
| --- | --- |
| 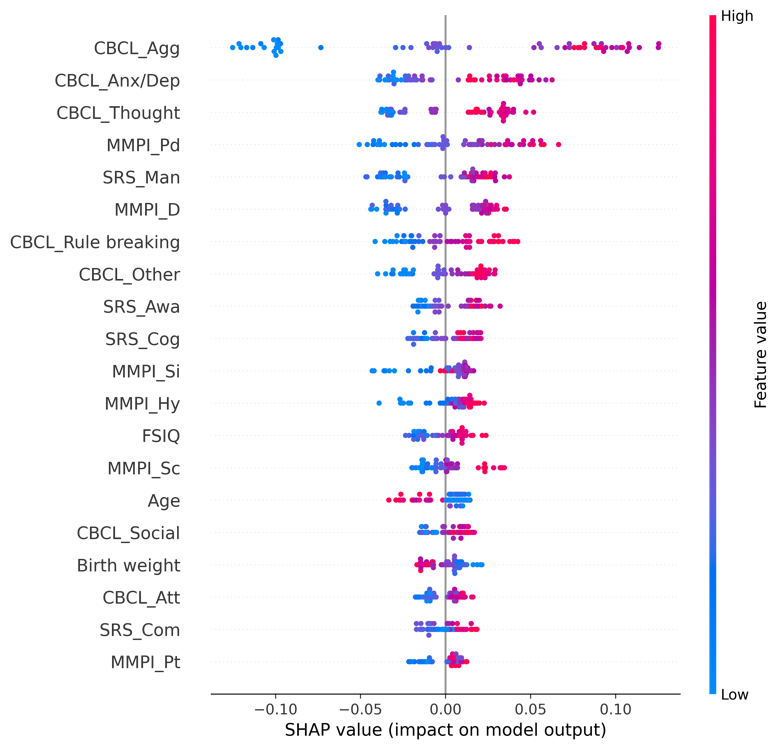 | 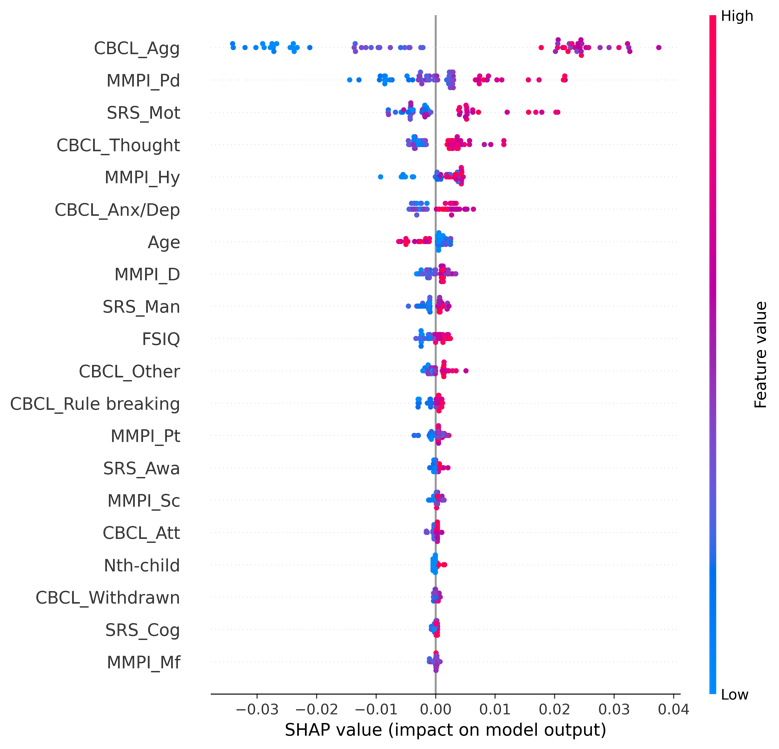 |

## eFigure 16.5. Receiver operating characteristic curves (ROC) for the training and test set

| <Training set> | <Test set> |
| --- | --- |
| 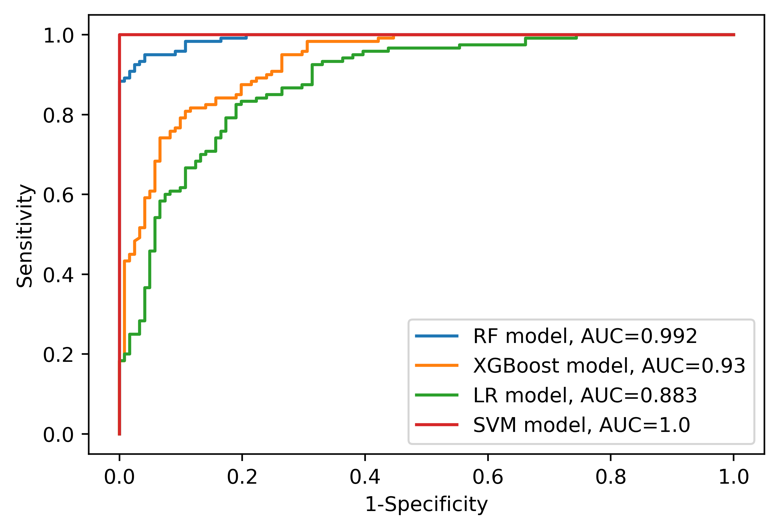 | 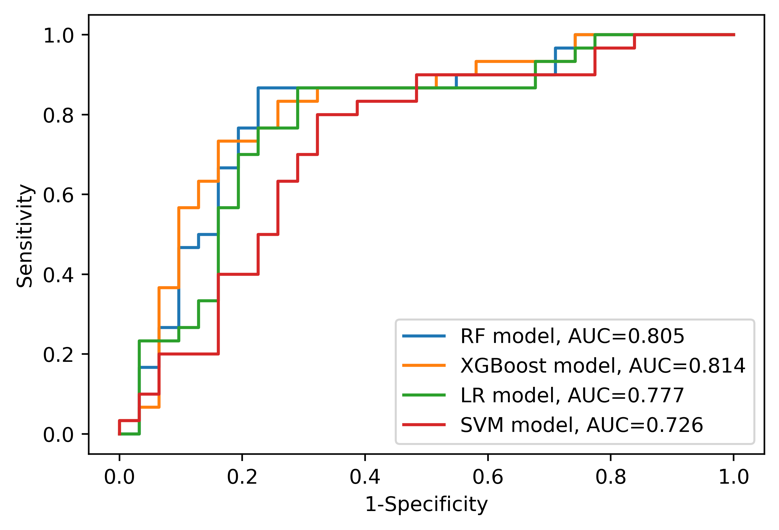 |

Abbreviations: AUC=area under curve, LR=logistic regression, RF=random forest, SVM=support vector machine, XGBoost=Extreme Gradient Boosting.

# **eAppendix 17. Model information and detailed results (Sample: CBCL 6-18 sample [n=302], Outcome: Total parenting stress)**

## eTable 17.1. Model features (Variables are on the second columns)

| **Caregiver variables** | - Assistant caregiver status [Yes/No]  - Current working status [Yes/No]  - Minnesota Multiphasic Personality Inventory (MMPI) Hypochondriasis (T-score)  - MMPI Depression (T-score)  - MMPI Hysteria (T-score)  - MMPI Psychopathy (T-score)  - MMPI Masculinity/Femininity (T-score)  - MMPI Paranoia (T-score)  - MMPI Psychasthenia (T-score)  - MMPI Schizophrenia (T-score)  - MMPI Hypomania (T-score)  - MMPI Social Introversion (T-score)  - The order of birth  - Number of children |
| --- | --- |
| **ASD patient variables** | - Age  - Sex [Male/Female]  - Family history of mental disorder [Yes/No]  - History of major disease [Yes/No]  - Psychotropic medication status [Drug free/Monotherapy of antipsychotics/Combined therapy of antipsychotics/Other psychotic medication]  - Gestational age  - Birth weight  - Mode of delivery [Vaginal delivery/Caesarean section]  - Existence of another child with mental disorder [Yes/No] [Yes/No]  - Full-Scale Intelligence Quotient (FSIQ)  - Social responsiveness scale (SRS) Social awareness (T-score)  - SRS Social cognition (T-score)  - SRS Social communication (T-score)  - SRS Social motivation (T-score)  - SRS Autistic mannerisms (T-score)  - Child Behavior Checklist (CBCL) Anxious/depressed (T-score)  - CBCL Withdrawn/depressed (T-score)  - CBCL Somatic complaints (T-score)  - CBCL Social problems (T-score)  - CBCL Thought problems (T-score)  - CBCL Attention problems (T-score)  - CBCL Rule-breaking behavior (T-score)  - CBCL Aggressive behavior (T-score)  - CBCL Other problems (T-score) |

## eTable 17.2. Performance of each model on the training set

|  | **RF model** | **XGBoost model** | **LR model** | **SVM model** |
| --- | --- | --- | --- | --- |
| **ROC AUC (95% CI)** | 0.966 (0.946-0.982) | 0.951 (0.926-0.971) | 0.881 (0.839-0.919) | 0.883 (0.842-0.920) |
| **Sensitivity (95% CI)** | 0.906 (0.852-0.953) | 0.875 (0.817-0.929) | 0.797 (0.726-0.865) | 0.781 (0.706-0.851) |
| **Specificity (95% CI)** | 0.858 (0.790-0.920) | 0.841 (0.771-0.907) | 0.761 (0.681-0.838) | 0.779 (0.701-0.852) |
| **PPV (95% CI)** | 0.879 (0.820-0.932) | 0.862 (0.800-0.919) | 0.791 (0.719-0.859) | 0.800 (0.728-0.868) |
| **NPV (95% CI)** | 0.890 (0.829-0.945) | 0.856 (0.788-0.917) | 0.768 (0.688-0.842) | 0.759 (0.679-0.835) |
| **Accuracy (95% CI)** | 0.884 (0.842-0.921) | 0.859 (0.813-0.900) | 0.780 (0.726-0.830) | 0.780 (0.726-0.830) |
| Abbreviations: AUC=area under curve, CI=confidence interval, NA=not available, NPV=negative predictive value, LR=logistic regression, RF=random forest, ROC=receiver operating characteristic, PPV=positive predictive value, SVM=support vector machine, XGBoost=Extreme Gradient Boosting. | | | | |

## eTable 17.3. Performance of each model on the test set

|  | **RF model** | **XGBoost model** | **LR model** | **SVM model** |
| --- | --- | --- | --- | --- |
| **ROC AUC (95% CI)** | 0.847 (0.734-0.940) | 0.747 (0.615-0.864) | 0.839 (0.725-0.933) | 0.842 (0.729-0.935) |
| **Sensitivity (95% CI)** | 0.812 (0.667-0.935) | 0.656 (0.485-0.818) | 0.656 (0.485-0.815) | 0.656 (0.485-0.815) |
| **Specificity (95% CI)** | 0.793 (0.633-0.929) | 0.690 (0.516-0.850) | 0.828 (0.679-0.962) | 0.897 (0.769-1.000) |
| **PPV (95% CI)** | 0.812 (0.667-0.935) | 0.700 (0.531-0.857) | 0.808 (0.643-0.957) | 0.875 (0.722-1.000) |
| **NPV (95% CI)** | 0.793 (0.633-0.929) | 0.645 (0.469-0.815) | 0.686 (0.526-0.833) | 0.703 (0.550-0.844) |
| **Accuracy (95% CI)** | 0.803 (0.705-0.902) | 0.672 (0.557-0.787) | 0.738 (0.623-0.836) | 0.770 (0.656-0.869) |
| Abbreviations: AUC=area under curve, CI=confidence interval, NA=not available, NPV=negative predictive value, LR=logistic regression, RF=random forest, ROC=receiver operating characteristic, PPV=positive predictive value, SVM=support vector machine, XGBoost=Extreme Gradient Boosting. | | | | |

## eFigure 17.4. SHapley Additive exPlanations (SHAP) summary plot for RF and XGBoost models (only top 20 predictors were presented)

| <RF model> | <XGBoost model> |
| --- | --- |
| 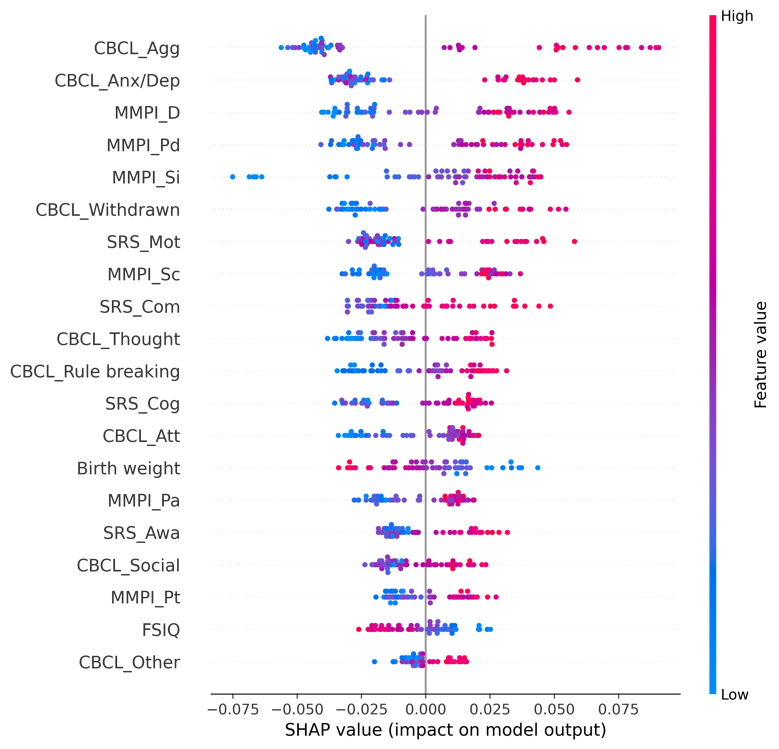 | 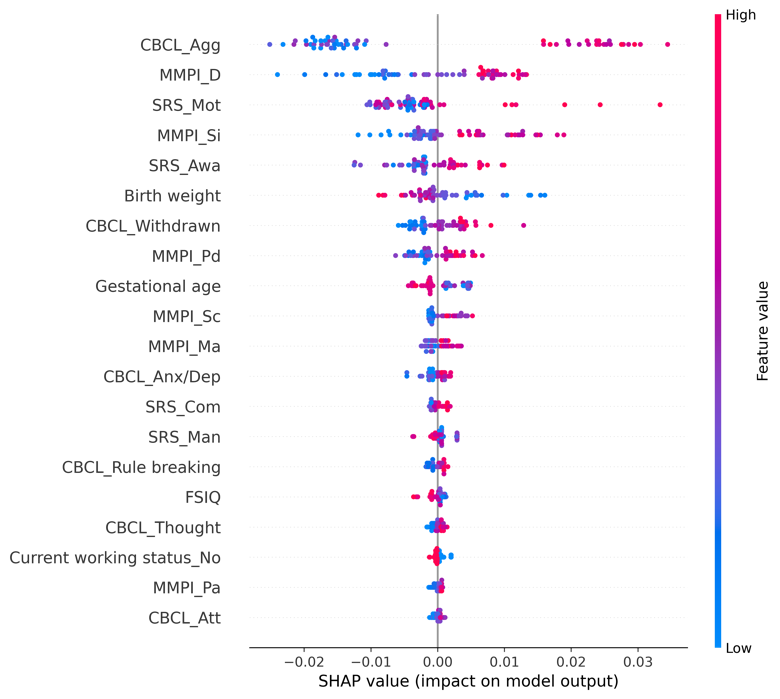 |

## eFigure 17.5. Receiver operating characteristic curves (ROC) for the training and test set

| <Training set> | <Test set> |
| --- | --- |
| 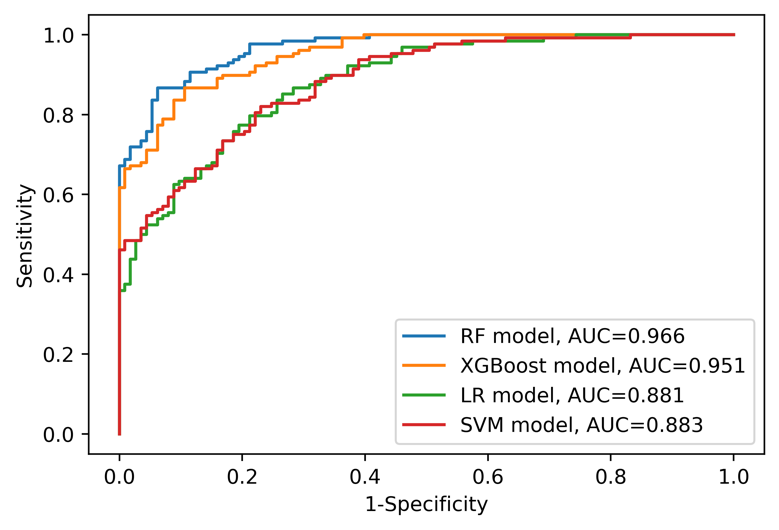 | 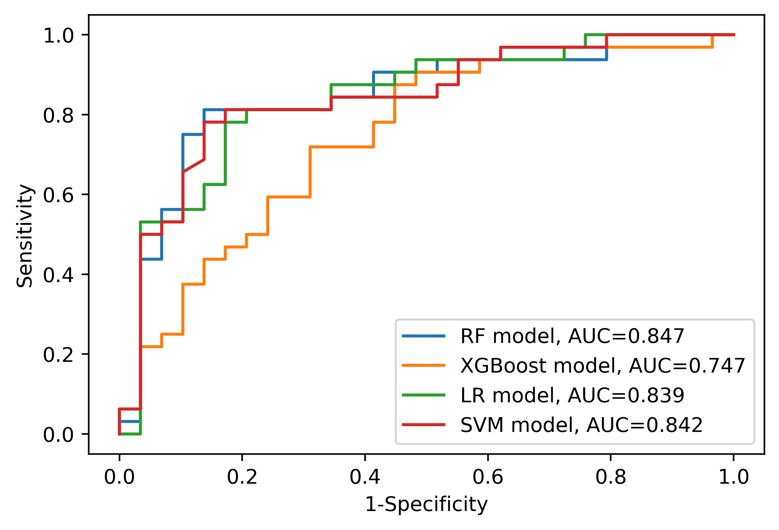 |

Abbreviations: AUC=area under curve, LR=logistic regression, RF=random forest, SVM=support vector machine, XGBoost=Extreme Gradient Boosting.

# **eAppendix 18. Model information and detailed results (Sample: with ADHD [n=168], Outcome: Parental distress)**

## eTable 18.1. Model features (Variables are on the second columns)

| **Caregiver variables** | - Assistant caregiver status [Yes/No]  - Current working status [Yes/No]  - Minnesota Multiphasic Personality Inventory (MMPI) Hypochondriasis (T-score)  - MMPI Depression (T-score)  - MMPI Hysteria (T-score)  - MMPI Psychopathy (T-score)  - MMPI Masculinity/Femininity (T-score)  - MMPI Paranoia (T-score)  - MMPI Psychasthenia (T-score)  - MMPI Schizophrenia (T-score)  - MMPI Hypomania (T-score)  - MMPI Social Introversion (T-score)  - The order of birth  - Number of children |
| --- | --- |
| **ASD patient variables** | - Age  - Sex [Male/Female]  - Family history of mental disorder [Yes/No]  - History of major disease [Yes/No]  - Psychotropic medication status [Drug free/Monotherapy of antipsychotics/Combined therapy of antipsychotics/Other psychotic medication]  - Gestational age  - Birth weight  - Mode of delivery [Vaginal delivery/Caesarean section]  - Existence of another child with mental disorder [Yes/No]  - Full-Scale Intelligence Quotient (FSIQ)  - Social responsiveness scale (SRS) Social awareness (T-score)  - SRS Social cognition (T-score)  - SRS Social communication (T-score)  - SRS Social motivation (T-score)  - SRS Autistic mannerisms (T-score)  - Child Behavior Checklist (CBCL) Anxious/depressed (T-score)  - CBCL Withdrawn/depressed (T-score)  - CBCL Somatic complaints (T-score)  - CBCL Attention problems (T-score)  - CBCL Aggressive behavior (T-score)  - CBCL Other problems (T-score) |

## eTable 18.2. Performance of each model on the training set

|  | **RF model** | **XGBoost model** | **LR model** | **SVM model** |
| --- | --- | --- | --- | --- |
| **ROC AUC (95% CI)** | 0.975 (0.950-0.992) | 0.898 (0.841-0.946) | 0.865 (0.800-0.922) | 1.000 (1.000-1.000) |
| **Sensitivity (95% CI)** | 0.705 (0.562-0.837) | 0.545 (0.395-0.690) | 0.568 (0.419-0.714) | 1.000 (1.000-1.000) |
| **Specificity (95% CI)** | 0.967 (0.925-1.000) | 0.944 (0.892-0.989) | 0.889 (0.821-0.949) | 1.000 (1.000-1.000) |
| **PPV (95% CI)** | 0.912 (0.806-1.000) | 0.828 (0.679-0.960) | 0.714 (0.559-0.857) | 1.000 (1.000-1.000) |
| **NPV (95% CI)** | 0.870 (0.802-0.932) | 0.810 (0.732-0.881) | 0.808 (0.729-0.881) | 1.000 (1.000-1.000) |
| **Accuracy (95% CI)** | 0.881 (0.821-0.933) | 0.813 (0.746-0.873) | 0.784 (0.709-0.851) | 1.000 (1.000-1.000) |
| Abbreviations: AUC=area under curve, CI=confidence interval, NA=not available, NPV=negative predictive value, LR=logistic regression, RF=random forest, ROC=receiver operating characteristic, PPV=positive predictive value, SVM=support vector machine, XGBoost=Extreme Gradient Boosting. | | | | |

## eTable 18.3. Performance of each model on the test set

|  | **RF model** | **XGBoost model** | **LR model** | **SVM model** |
| --- | --- | --- | --- | --- |
| **ROC AUC (95% CI)** | 0.794 (0.577-0.970) | 0.818 (0.628-0.968) | 0.739 (0.493-0.949) | 0.771 (0.553-0.945) |
| **Sensitivity (95% CI)** | 0.455 (0.143-0.769) | 0.455 (0.143-0.769) | 0.364 (0.091-0.667) | 0.545 (0.231-0.857) |
| **Specificity (95% CI)** | 1.000 (1.000-1.000) | 1.000 (1.000-1.000) | 0.957 (0.857-1.000) | 0.870 (0.714-1.000) |
| **PPV (95% CI)** | 1.000 (1.000-1.000) | 1.000 (1.000-1.000) | 0.800 (0.333-1.000) | 0.667 (0.333-1.000) |
| **NPV (95% CI)** | 0.793 (0.643-0.931) | 0.793 (0.643-0.931) | 0.759 (0.594-0.900) | 0.800 (0.630-0.952) |
| **Accuracy (95% CI)** | 0.824 (0.676-0.941) | 0.824 (0.676-0.941) | 0.765 (0.618-0.912) | 0.765 (0.618-0.912) |
| Abbreviations: AUC=area under curve, CI=confidence interval, NA=not available, NPV=negative predictive value, LR=logistic regression, RF=random forest, ROC=receiver operating characteristic, PPV=positive predictive value, SVM=support vector machine, XGBoost=Extreme Gradient Boosting. | | | | |

## eFigure 18.4. SHapley Additive exPlanations (SHAP) summary plot for RF and XGBoost models (only top 20 predictors were presented)

| <RF model> | <XGBoost model> |
| --- | --- |
| 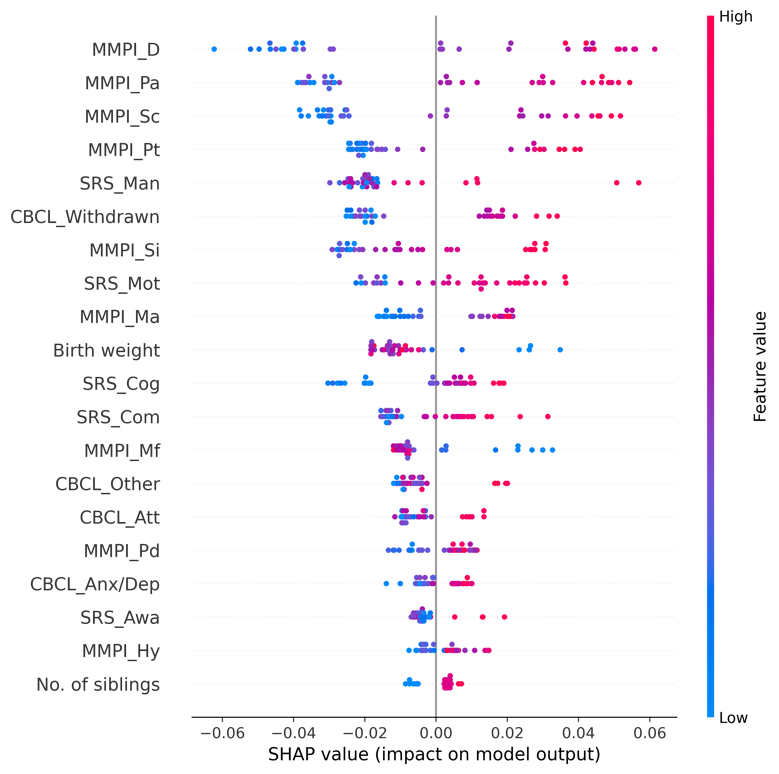 | 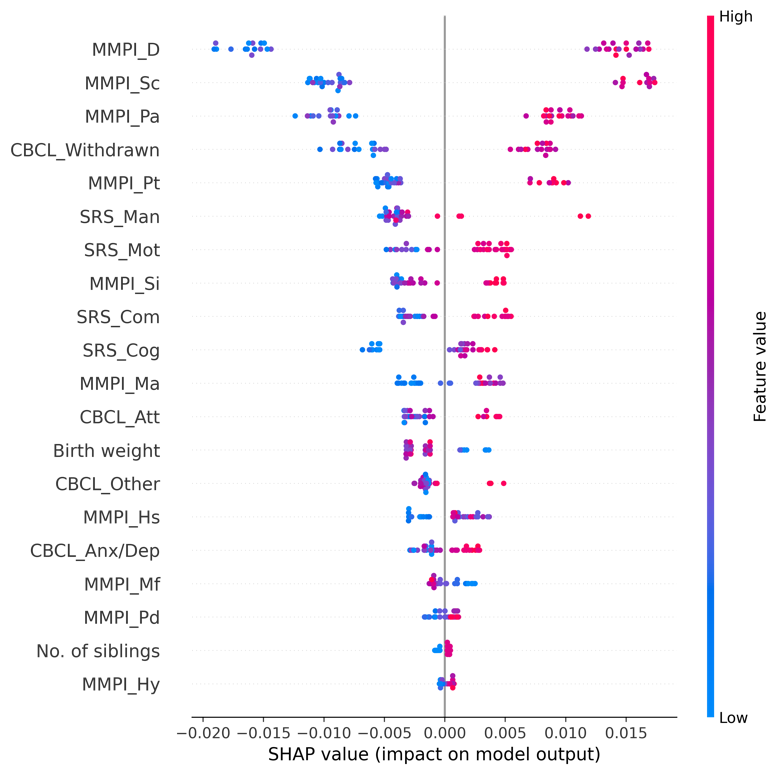 |

## eFigure 18.5. Receiver operating characteristic curves (ROC) for the training and test set

| <Training set> | <Test set> |
| --- | --- |
| 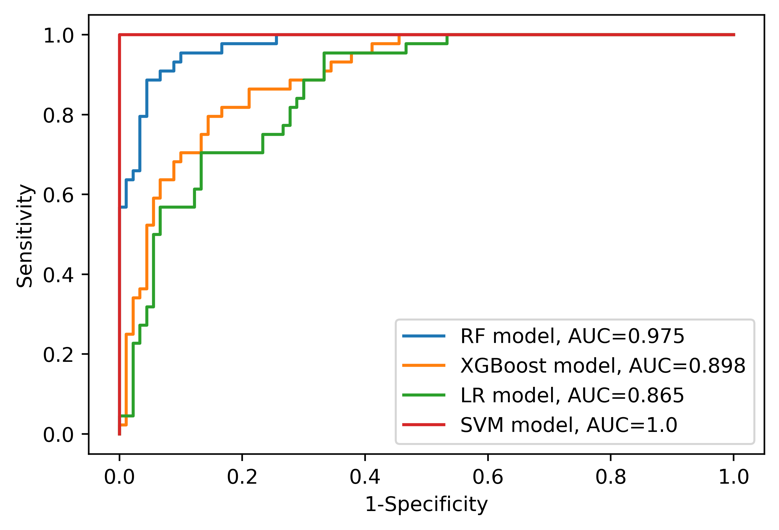 | 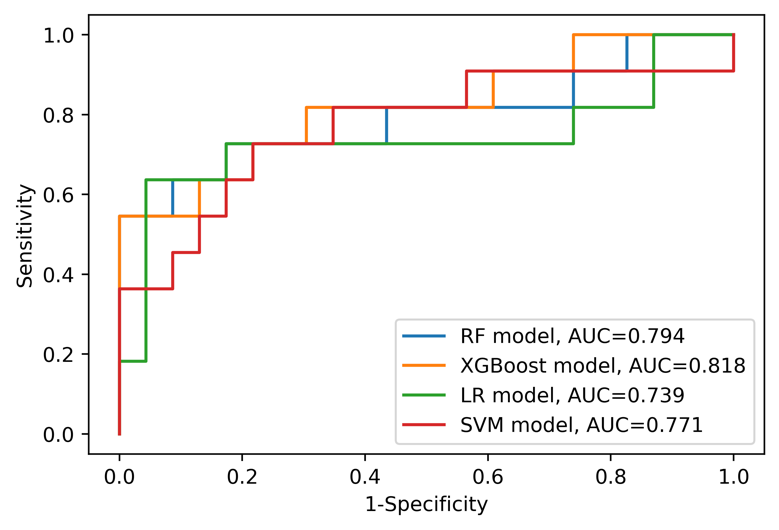 |

Abbreviations: AUC=area under curve, LR=logistic regression, RF=random forest, SVM=support vector machine, XGBoost=Extreme Gradient Boosting.

# **eAppendix 19. Model information and detailed results (Sample: with ADHD [n=168], Outcome: Parent-child dysfunctional interaction)**

## eTable 19.1. Model features (Variables are on the second columns)

| **Caregiver variables** | - Assistant caregiver status [Yes/No]  - Current working status [Yes/No]  - Minnesota Multiphasic Personality Inventory (MMPI) Hypochondriasis (T-score)  - MMPI Depression (T-score)  - MMPI Hysteria (T-score)  - MMPI Psychopathy (T-score)  - MMPI Masculinity/Femininity (T-score)  - MMPI Paranoia (T-score)  - MMPI Psychasthenia (T-score)  - MMPI Schizophrenia (T-score)  - MMPI Hypomania (T-score)  - MMPI Social Introversion (T-score)  - The order of birth  - Number of children |
| --- | --- |
| **ASD patient variables** | - Age  - Sex [Male/Female]  - Family history of mental disorder [Yes/No]  - History of major disease [Yes/No]  - Psychotropic medication status [Drug free/Monotherapy of antipsychotics/Combined therapy of antipsychotics/Other psychotic medication]  - Gestational age  - Birth weight  - Mode of delivery [Vaginal delivery/Caesarean section]  - Existence of another child with mental disorder [Yes/No]  - Full-Scale Intelligence Quotient (FSIQ)  - Social responsiveness scale (SRS) Social awareness (T-score)  - SRS Social cognition (T-score)  - SRS Social communication (T-score)  - SRS Social motivation (T-score)  - SRS Autistic mannerisms (T-score)  - Child Behavior Checklist (CBCL) Anxious/depressed (T-score)  - CBCL Withdrawn/depressed (T-score)  - CBCL Somatic complaints (T-score)  - CBCL Attention problems (T-score)  - CBCL Aggressive behavior (T-score)  - CBCL Other problems (T-score) |

## eTable 19.2. Performance of each model on the training set

|  | **RF model** | **XGBoost model** | **LR model** | **SVM model** |
| --- | --- | --- | --- | --- |
| **ROC AUC (95% CI)** | 0.943 (0.902-0.976) | 0.896 (0.835-0.947) | 0.859 (0.791-0.918) | 0.824 (0.749-0.890) |
| **Sensitivity (95% CI)** | 0.776 (0.667-0.879) | 0.655 (0.529-0.776) | 0.672 (0.549-0.790) | 0.552 (0.424-0.679) |
| **Specificity (95% CI)** | 0.921 (0.855-0.974) | 0.921 (0.855-0.975) | 0.829 (0.740-0.909) | 0.855 (0.771-0.929) |
| **PPV (95% CI)** | 0.882 (0.787-0.962) | 0.864 (0.750-0.957) | 0.750 (0.629-0.864) | 0.744 (0.610-0.870) |
| **NPV (95% CI)** | 0.843 (0.761-0.919) | 0.778 (0.688-0.862) | 0.768 (0.674-0.857) | 0.714 (0.618-0.804) |
| **Accuracy (95% CI)** | 0.858 (0.799-0.910) | 0.806 (0.739-0.873) | 0.761 (0.687-0.828) | 0.724 (0.649-0.799) |
| Abbreviations: AUC=area under curve, CI=confidence interval, NA=not available, NPV=negative predictive value, LR=logistic regression, RF=random forest, ROC=receiver operating characteristic, PPV=positive predictive value, SVM=support vector machine, XGBoost=Extreme Gradient Boosting. | | | | |

## eTable 19.3. Performance of each model on the test set

|  | **RF model** | **XGBoost model** | **LR model** | **SVM model** |
| --- | --- | --- | --- | --- |
| **ROC AUC (95% CI)** | 0.884 (0.751-0.979) | 0.874 (0.730-0.979) | 0.839 (0.689-0.951) | 0.877 (0.743-0.969) |
| **Sensitivity (95% CI)** | 0.667 (0.412-0.909) | 0.467 (0.214-0.727) | 0.600 (0.350-0.857) | 0.533 (0.273-0.800) |
| **Specificity (95% CI)** | 0.842 (0.667-1.000) | 0.895 (0.737-1.000) | 0.895 (0.737-1.000) | 1.000 (1.000-1.000) |
| **PPV (95% CI)** | 0.769 (0.500-1.000) | 0.778 (0.462-1.000) | 0.818 (0.556-1.000) | 1.000 (1.000-1.000) |
| **NPV (95% CI)** | 0.762 (0.571-0.941) | 0.680 (0.500-0.857) | 0.739 (0.550-0.913) | 0.731 (0.556-0.895) |
| **Accuracy (95% CI)** | 0.765 (0.618-0.912) | 0.706 (0.559-0.853) | 0.765 (0.618-0.882) | 0.794 (0.647-0.912) |
| Abbreviations: AUC=area under curve, CI=confidence interval, NA=not available, NPV=negative predictive value, LR=logistic regression, RF=random forest, ROC=receiver operating characteristic, PPV=positive predictive value, SVM=support vector machine, XGBoost=Extreme Gradient Boosting. | | | | |

## eFigure 19.4. SHapley Additive exPlanations (SHAP) summary plot for RF and XGBoost models (only top 20 predictors were presented)

| <RF model> | <XGBoost model> |
| --- | --- |
| 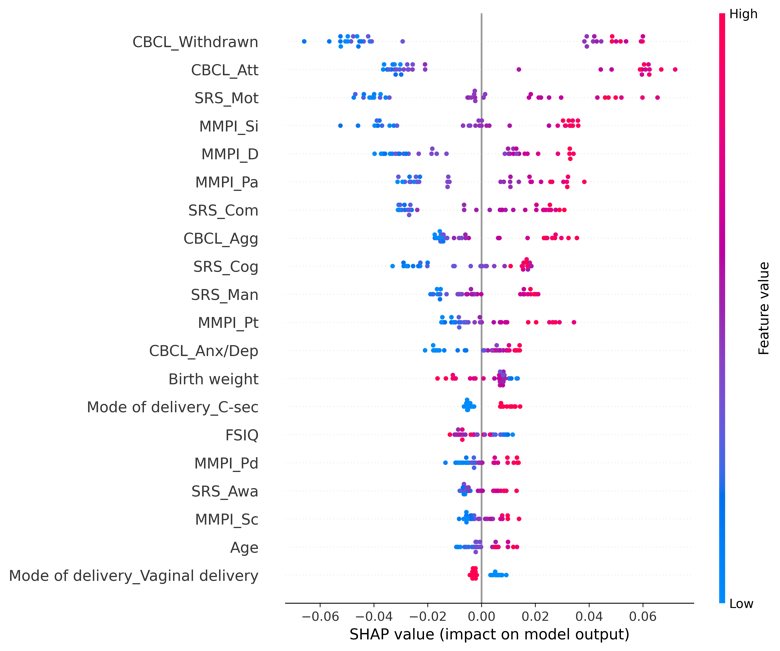 | 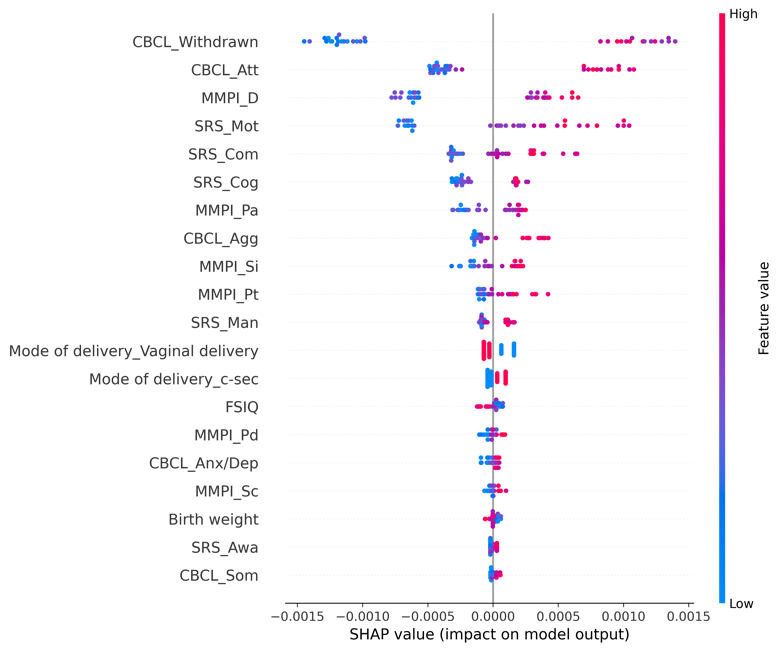 |

## eFigure 19.5. Receiver operating characteristic curves (ROC) for the training and test set

| <Training set> | <Test set> |
| --- | --- |
| 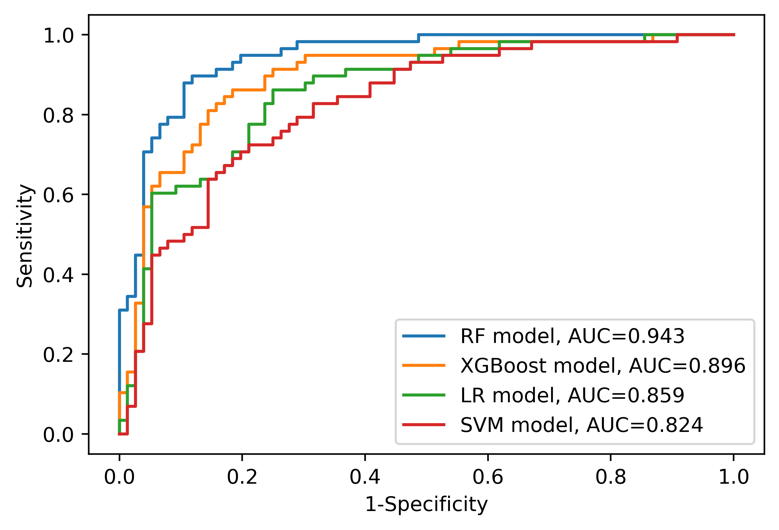 | 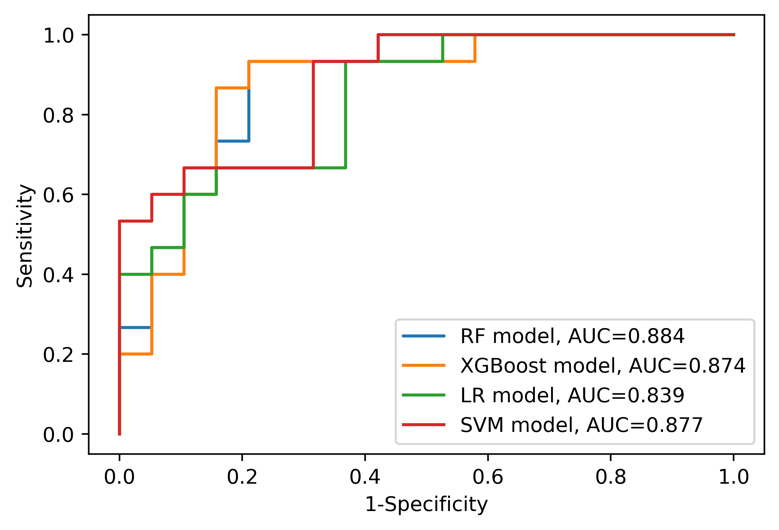 |

Abbreviations: AUC=area under curve, LR=logistic regression, RF=random forest, SVM=support vector machine, XGBoost=Extreme Gradient Boosting.

# **eAppendix 20. Model information and detailed results (Sample: with ADHD [n=168], Outcome: Difficult child)**

## eTable 20.1. Model features (Variables are on the second columns)

| **Caregiver variables** | - Assistant caregiver status [Yes/No]  - Current working status [Yes/No]  - Minnesota Multiphasic Personality Inventory (MMPI) Hypochondriasis (T-score)  - MMPI Depression (T-score)  - MMPI Hysteria (T-score)  - MMPI Psychopathy (T-score)  - MMPI Masculinity/Femininity (T-score)  - MMPI Paranoia (T-score)  - MMPI Psychasthenia (T-score)  - MMPI Schizophrenia (T-score)  - MMPI Hypomania (T-score)  - MMPI Social Introversion (T-score)  - The order of birth  - Number of children |
| --- | --- |
| **ASD patient variables** | - Age  - Sex [Male/Female]  - Family history of mental disorder [Yes/No]  - History of major disease [Yes/No]  - Psychotropic medication status [Drug free/Monotherapy of antipsychotics/Combined therapy of antipsychotics/Other psychotic medication]  - Gestational age  - Birth weight  - Mode of delivery [Vaginal delivery/Caesarean section]  - Existence of another child with mental disorder [Yes/No]  - Full-Scale Intelligence Quotient (FSIQ)  - Social responsiveness scale (SRS) Social awareness (T-score)  - SRS Social cognition (T-score)  - SRS Social communication (T-score)  - SRS Social motivation (T-score)  - SRS Autistic mannerisms (T-score)  - Child Behavior Checklist (CBCL) Anxious/depressed (T-score)  - CBCL Withdrawn/depressed (T-score)  - CBCL Somatic complaints (T-score)  - CBCL Attention problems (T-score)  - CBCL Aggressive behavior (T-score)  - CBCL Other problems (T-score) |

## eTable 20.2. Performance of each model on the training set

|  | **RF model** | **XGBoost model** | **LR model** | **SVM model** |
| --- | --- | --- | --- | --- |
| **ROC AUC (95% CI)** | 0.969 (0.942-0.989) | 0.899 (0.844-0.947) | 0.839 (0.768-0.905) | 0.144 (0.084-0.210) |
| **Sensitivity (95% CI)** | 0.910 (0.838-0.971) | 0.746 (0.640-0.845) | 0.701 (0.589-0.809) | 0.716 (0.607-0.822) |
| **Specificity (95% CI)** | 0.910 (0.836-0.971) | 0.851 (0.759-0.932) | 0.821 (0.723-0.910) | 0.806 (0.703-0.896) |
| **PPV (95% CI)** | 0.910 (0.836-0.971) | 0.833 (0.732-0.923) | 0.797 (0.690-0.897) | 0.787 (0.679-0.884) |
| **NPV (95% CI)** | 0.910 (0.838-0.971) | 0.770 (0.671-0.862) | 0.733 (0.630-0.829) | 0.740 (0.636-0.836) |
| **Accuracy (95% CI)** | 0.910 (0.858-0.955) | 0.799 (0.731-0.866) | 0.761 (0.687-0.828) | 0.761 (0.687-0.828) |
| Abbreviations: AUC=area under curve, CI=confidence interval, NA=not available, NPV=negative predictive value, LR=logistic regression, RF=random forest, ROC=receiver operating characteristic, PPV=positive predictive value, SVM=support vector machine, XGBoost=Extreme Gradient Boosting. | | | | |

## eTable 20.3. Performance of each model on the test set

|  | **RF model** | **XGBoost model** | **LR model** | **SVM model** |
| --- | --- | --- | --- | --- |
| **ROC AUC (95% CI)** | 0.709 (0.514-0.872) | 0.678 (0.477-0.864) | 0.657 (0.453-0.837) | 0.315 (0.144-0.516) |
| **Sensitivity (95% CI)** | 0.706 (0.471-0.917) | 0.765 (0.545-0.944) | 0.588 (0.353-0.812) | 0.588 (0.353-0.812) |
| **Specificity (95% CI)** | 0.588 (0.353-0.818) | 0.647 (0.412-0.875) | 0.529 (0.286-0.765) | 0.529 (0.286-0.765) |
| **PPV (95% CI)** | 0.632 (0.400-0.842) | 0.684 (0.450-0.889) | 0.556 (0.312-0.786) | 0.556 (0.312-0.786) |
| **NPV (95% CI)** | 0.667 (0.412-0.900) | 0.733 (0.500-0.933) | 0.562 (0.308-0.812) | 0.562 (0.308-0.812) |
| **Accuracy (95% CI)** | 0.647 (0.500-0.794) | 0.706 (0.559-0.853) | 0.559 (0.382-0.735) | 0.559 (0.382-0.735) |
| Abbreviations: AUC=area under curve, CI=confidence interval, NA=not available, NPV=negative predictive value, LR=logistic regression, RF=random forest, ROC=receiver operating characteristic, PPV=positive predictive value, SVM=support vector machine, XGBoost=Extreme Gradient Boosting. | | | | |

## eFigure 20.4. SHapley Additive exPlanations (SHAP) summary plot for RF and XGBoost models (only top 20 predictors were presented)

| <RF model> | <XGBoost model> |
| --- | --- |
| 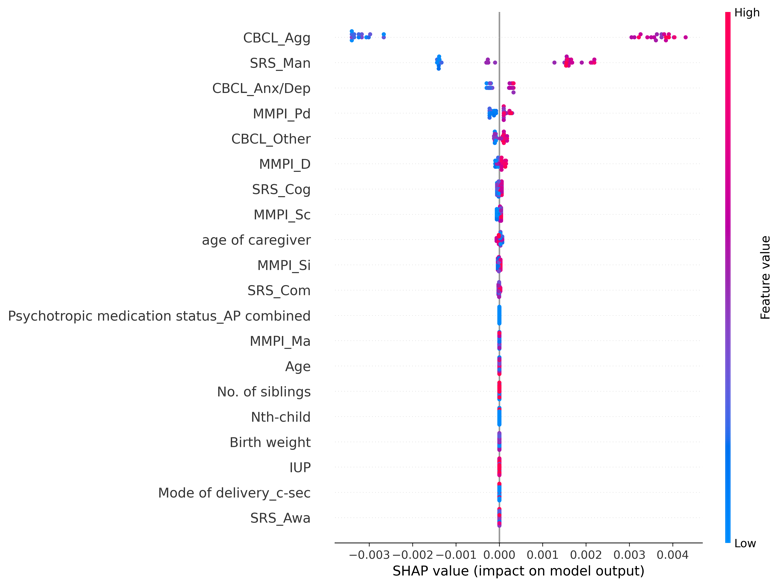 | 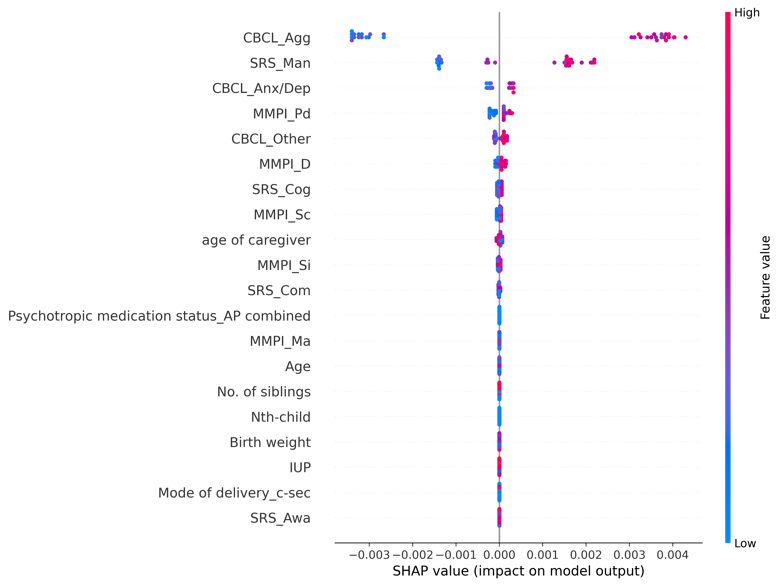 |

## eFigure 20.5. Receiver operating characteristic curves (ROC) for the training and test set

| <Training set> | <Test set> |
| --- | --- |
| 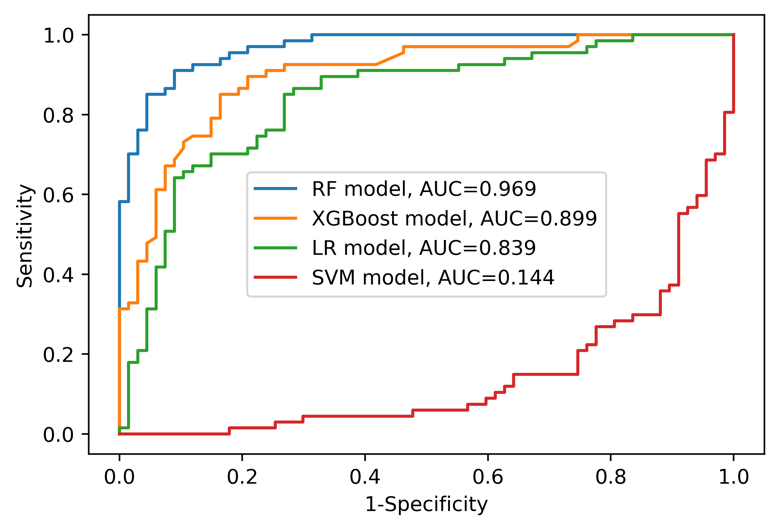 | 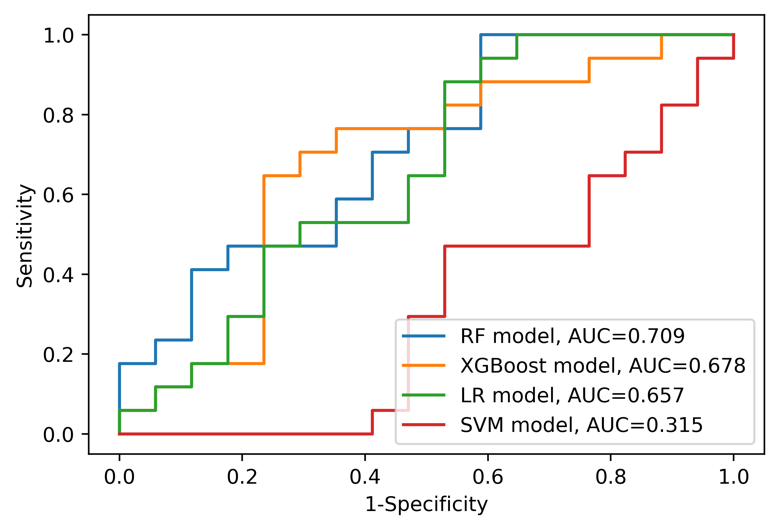 |

Abbreviations: AUC=area under curve, LR=logistic regression, RF=random forest, SVM=support vector machine, XGBoost=Extreme Gradient Boosting.

# **eAppendix 21. Model information and detailed results (Sample: with ADHD [n=168], Outcome: Total parenting stress)**

## eTable 21.1. Model features (Variables are on the second columns)

| **Caregiver variables** | - Assistant caregiver status [Yes/No]  - Current working status [Yes/No]  - Minnesota Multiphasic Personality Inventory (MMPI) Hypochondriasis (T-score)  - MMPI Depression (T-score)  - MMPI Hysteria (T-score)  - MMPI Psychopathy (T-score)  - MMPI Masculinity/Femininity (T-score)  - MMPI Paranoia (T-score)  - MMPI Psychasthenia (T-score)  - MMPI Schizophrenia (T-score)  - MMPI Hypomania (T-score)  - MMPI Social Introversion (T-score)  - The order of birth  - Number of children |
| --- | --- |
| **ASD patient variables** | - Age  - Sex [Male/Female]  - Family history of mental disorder [Yes/No]  - History of major disease [Yes/No]  - Psychotropic medication status [Drug free/Monotherapy of antipsychotics/Combined therapy of antipsychotics/Other psychotic medication]  - Gestational age  - Birth weight  - Mode of delivery [Vaginal delivery/Caesarean section]  - Existence of another child with mental disorder [Yes/No]  - Full-Scale Intelligence Quotient (FSIQ)  - Social responsiveness scale (SRS) Social awareness (T-score)  - SRS Social cognition (T-score)  - SRS Social communication (T-score)  - SRS Social motivation (T-score)  - SRS Autistic mannerisms (T-score)  - Child Behavior Checklist (CBCL) Anxious/depressed (T-score)  - CBCL Withdrawn/depressed (T-score)  - CBCL Somatic complaints (T-score)  - CBCL Attention problems (T-score)  - CBCL Aggressive behavior (T-score)  - CBCL Other problems (T-score) |

## eTable 21.2. Performance of each model on the training set

|  | **RF model** | **XGBoost model** | **LR model** | **SVM model** |
| --- | --- | --- | --- | --- |
| **ROC AUC (95% CI)** | 0.977 (0.953-0.994) | 0.924 (0.874-0.963) | 0.825 (0.750-0.891) | 0.864 (0.799-0.918) |
| **Sensitivity (95% CI)** | 0.928 (0.862-0.985) | 0.884 (0.803-0.956) | 0.783 (0.682-0.877) | 1.000 (1.000-1.000) |
| **Specificity (95% CI)** | 0.877 (0.790-0.951) | 0.815 (0.716-0.905) | 0.692 (0.579-0.800) | 0.000 (0.000-0.000) |
| **PPV (95% CI)** | 0.889 (0.810-0.956) | 0.836 (0.744-0.914) | 0.730 (0.627-0.826) | 0.515 (0.425-0.597) |
| **NPV (95% CI)** | 0.919 (0.845-0.983) | 0.869 (0.778-0.948) | 0.750 (0.635-0.857) | NA |
| **Accuracy (95% CI)** | 0.903 (0.851-0.948) | 0.851 (0.791-0.910) | 0.739 (0.664-0.813) | 0.515 (0.425-0.597) |
| Abbreviations: AUC=area under curve, CI=confidence interval, NA=not available, NPV=negative predictive value, LR=logistic regression, RF=random forest, ROC=receiver operating characteristic, PPV=positive predictive value, SVM=support vector machine, XGBoost=Extreme Gradient Boosting. | | | | |

## eTable 21.3. Performance of each model on the test set

|  | **RF model** | **XGBoost model** | **LR model** | **SVM model** |
| --- | --- | --- | --- | --- |
| **ROC AUC (95% CI)** | 0.865 (0.726-0.969) | 0.834 (0.681-0.954) | 0.886 (0.754-0.985) | 0.886 (0.751-0.983) |
| **Sensitivity (95% CI)** | 0.882 (0.706-1.000) | 0.824 (0.625-1.000) | 0.882 (0.706-1.000) | 1.000 (1.000-1.000) |
| **Specificity (95% CI)** | 0.706 (0.474-0.917) | 0.706 (0.474-0.917) | 0.529 (0.286-0.769) | 0.000 (0.000-0.000) |
| **PPV (95% CI)** | 0.750 (0.550-0.938) | 0.737 (0.529-0.933) | 0.652 (0.455-0.842) | 0.500 (0.324-0.676) |
| **NPV (95% CI)** | 0.857 (0.643-1.000) | 0.800 (0.571-1.000) | 0.818 (0.556-1.000) | NA |
| **Accuracy (95% CI)** | 0.794 (0.647-0.912) | 0.765 (0.618-0.912) | 0.706 (0.559-0.853) | 0.500 (0.324-0.676) |
| Abbreviations: AUC=area under curve, CI=confidence interval, NA=not available, NPV=negative predictive value, LR=logistic regression, RF=random forest, ROC=receiver operating characteristic, PPV=positive predictive value, SVM=support vector machine, XGBoost=Extreme Gradient Boosting. | | | | |

## eFigure 21.4. SHapley Additive exPlanations (SHAP) summary plot for RF and XGBoost models (only top 20 predictors were presented)

| <RF model> | <XGBoost model> |
| --- | --- |
| 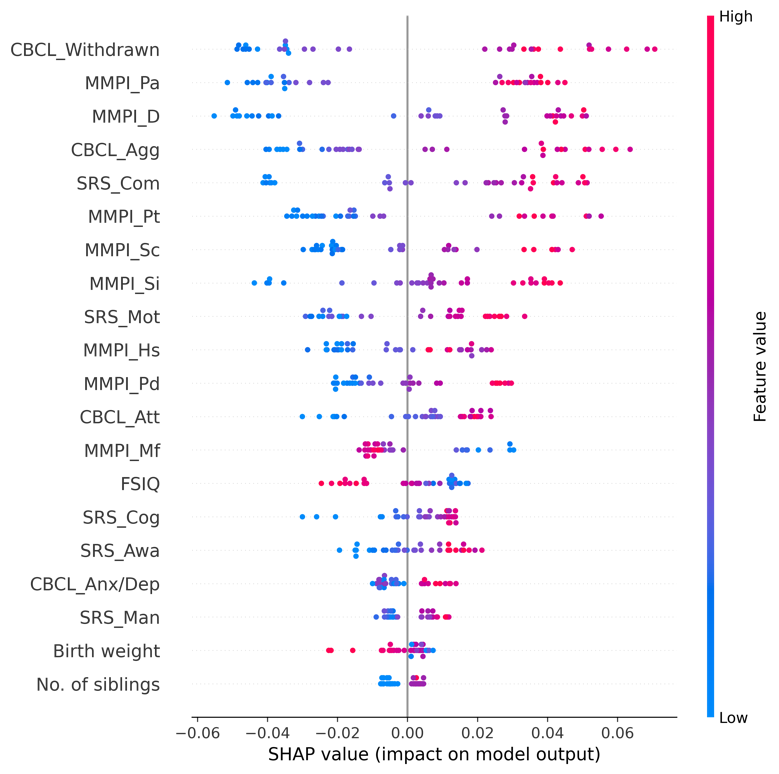 | 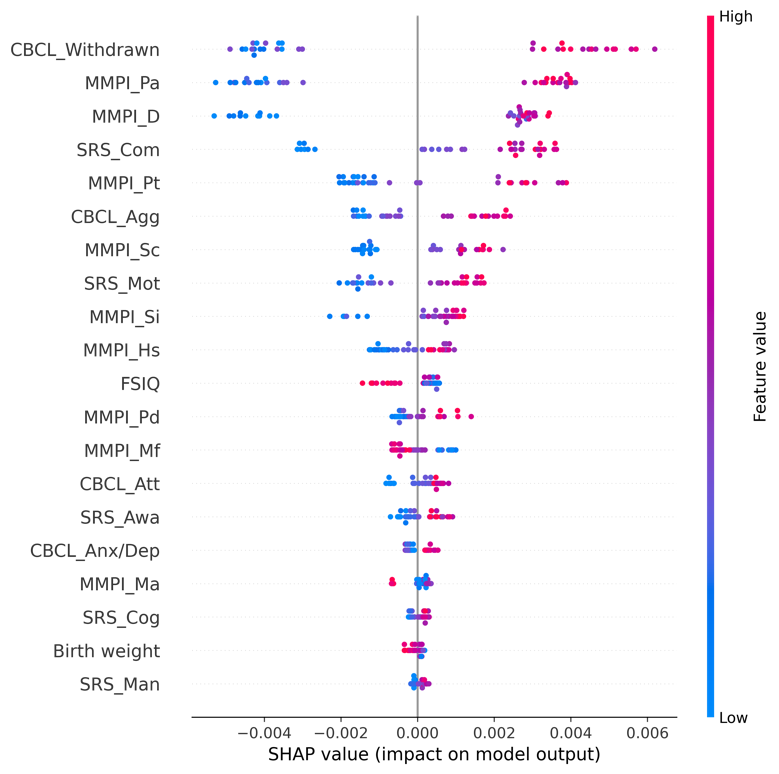 |

## eFigure 21.5. Receiver operating characteristic curves (ROC) for the training and test set

| <Training set> | <Test set> |
| --- | --- |
| 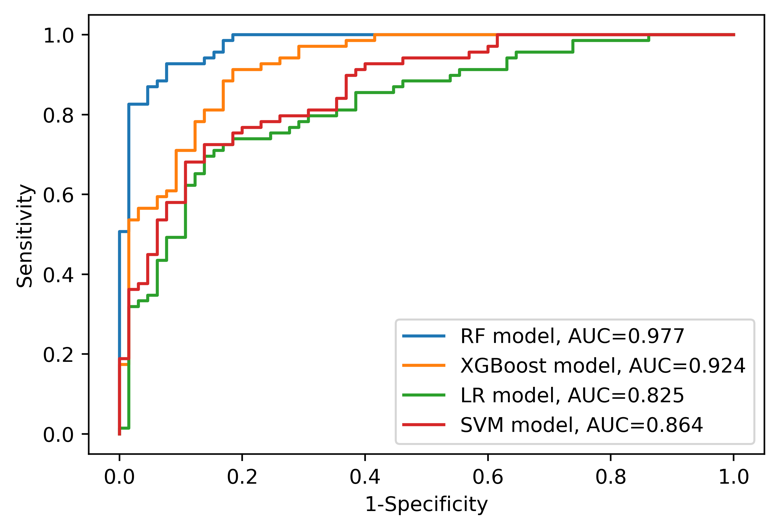 | 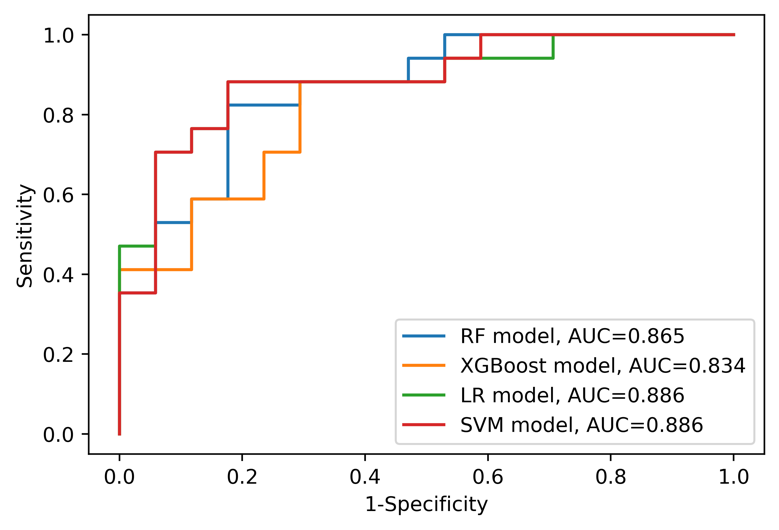 |

Abbreviations: AUC=area under curve, LR=logistic regression, RF=random forest, SVM=support vector machine, XGBoost=Extreme Gradient Boosting.

# **eAppendix 22. Model information and detailed results (Sample: without ADHD [n=328], Outcome: Parental distress)**

## eTable 22.1. Model features (Variables are on the second columns)

| **Caregiver variables** | - Assistant caregiver status [Yes/No]  - Current working status [Yes/No]  - Minnesota Multiphasic Personality Inventory (MMPI) Hypochondriasis (T-score)  - MMPI Depression (T-score)  - MMPI Hysteria (T-score)  - MMPI Psychopathy (T-score)  - MMPI Masculinity/Femininity (T-score)  - MMPI Paranoia (T-score)  - MMPI Psychasthenia (T-score)  - MMPI Schizophrenia (T-score)  - MMPI Hypomania (T-score)  - MMPI Social Introversion (T-score)  - The order of birth  - Number of children |
| --- | --- |
| **ASD patient variables** | - Age  - Sex [Male/Female]  - Family history of mental disorder [Yes/No]  - History of major disease [Yes/No]  - Psychotropic medication status [Drug free/Monotherapy of antipsychotics/Combined therapy of antipsychotics/Other psychotic medication]  - Gestational age  - Birth weight  - Mode of delivery [Vaginal delivery/Caesarean section]  - Existence of another child with mental disorder [Yes/No]  - Full-Scale Intelligence Quotient (FSIQ)  - Social responsiveness scale (SRS) Social awareness (T-score)  - SRS Social cognition (T-score)  - SRS Social communication (T-score)  - SRS Social motivation (T-score)  - SRS Autistic mannerisms (T-score)  - Child Behavior Checklist (CBCL) Anxious/depressed (T-score)  - CBCL Withdrawn/depressed (T-score)  - CBCL Somatic complaints (T-score)  - CBCL Attention problems (T-score)  - CBCL Aggressive behavior (T-score)  - CBCL Other problems (T-score) |

## eTable 22.2. Performance of each model on the training set

|  | **RF model** | **XGBoost model** | **LR model** | **SVM model** |
| --- | --- | --- | --- | --- |
| **ROC AUC (95% CI)** | 0.964 (0.943-0.981) | 0.908 (0.870-0.941) | 0.826 (0.766-0.879) | 0.840 (0.780-0.893) |
| **Sensitivity (95% CI)** | 0.577 (0.459-0.691) | 0.479 (0.361-0.595) | 0.310 (0.203-0.419) | 0.507 (0.391-0.625) |
| **Specificity (95% CI)** | 0.990 (0.973-1.000) | 0.979 (0.956-0.995) | 0.979 (0.956-0.995) | 0.932 (0.894-0.964) |
| **PPV (95% CI)** | 0.953 (0.879-1.000) | 0.895 (0.784-0.977) | 0.846 (0.692-0.966) | 0.735 (0.609-0.854) |
| **NPV (95% CI)** | 0.863 (0.816-0.906) | 0.835 (0.785-0.882) | 0.792 (0.739-0.843) | 0.836 (0.785-0.883) |
| **Accuracy (95% CI)** | 0.878 (0.836-0.916) | 0.844 (0.798-0.885) | 0.798 (0.748-0.844) | 0.817 (0.771-0.863) |
| Abbreviations: AUC=area under curve, CI=confidence interval, NA=not available, NPV=negative predictive value, LR=logistic regression, RF=random forest, ROC=receiver operating characteristic, PPV=positive predictive value, SVM=support vector machine, XGBoost=Extreme Gradient Boosting. | | | | |

## eTable 22.3. Performance of each model on the test set

|  | **RF model** | **XGBoost model** | **LR model** | **SVM model** |
| --- | --- | --- | --- | --- |
| **ROC AUC (95% CI)** | 0.781 (0.654-0.887) | 0.765 (0.637-0.875) | 0.828 (0.713-0.923) | 0.843 (0.733-0.931) |
| **Sensitivity (95% CI)** | 0.389 (0.167-0.632) | 0.222 (0.050-0.438) | 0.333 (0.118-0.565) | 0.500 (0.267-0.750) |
| **Specificity (95% CI)** | 0.938 (0.860-1.000) | 0.958 (0.894-1.000) | 1.000 (1.000-1.000) | 0.938 (0.860-1.000) |
| **PPV (95% CI)** | 0.700 (0.375-1.000) | 0.667 (0.200-1.000) | 1.000 (1.000-1.000) | 0.750 (0.500-1.000) |
| **NPV (95% CI)** | 0.804 (0.692-0.902) | 0.767 (0.655-0.869) | 0.800 (0.694-0.898) | 0.833 (0.727-0.927) |
| **Accuracy (95% CI)** | 0.788 (0.682-0.879) | 0.758 (0.652-0.864) | 0.818 (0.712-0.909) | 0.818 (0.712-0.909) |
| Abbreviations: AUC=area under curve, CI=confidence interval, NA=not available, NPV=negative predictive value, LR=logistic regression, RF=random forest, ROC=receiver operating characteristic, PPV=positive predictive value, SVM=support vector machine, XGBoost=Extreme Gradient Boosting. | | | | |

##

## eFigure 22.4. SHapley Additive exPlanations (SHAP) summary plot for RF and XGBoost models (only top 20 predictors were presented)

| <RF model> | <XGBoost model> |
| --- | --- |
| 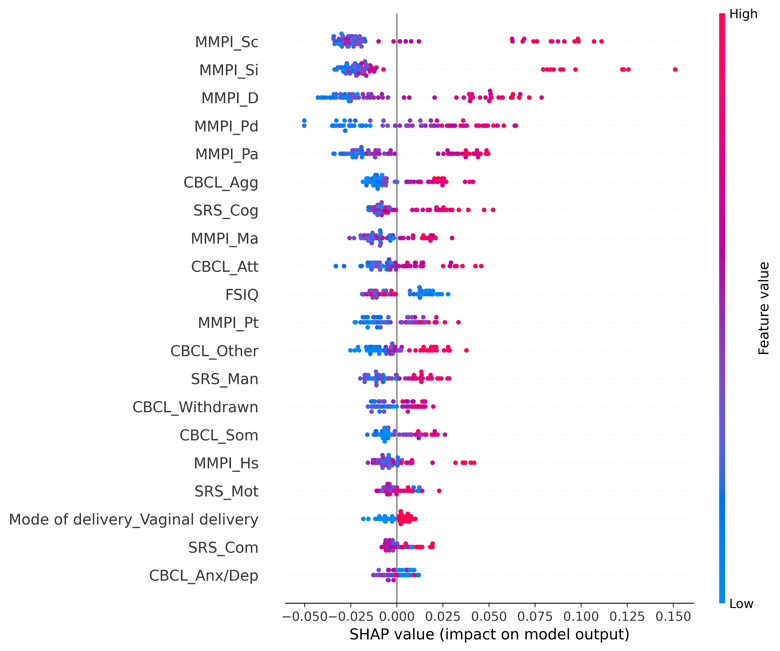 | 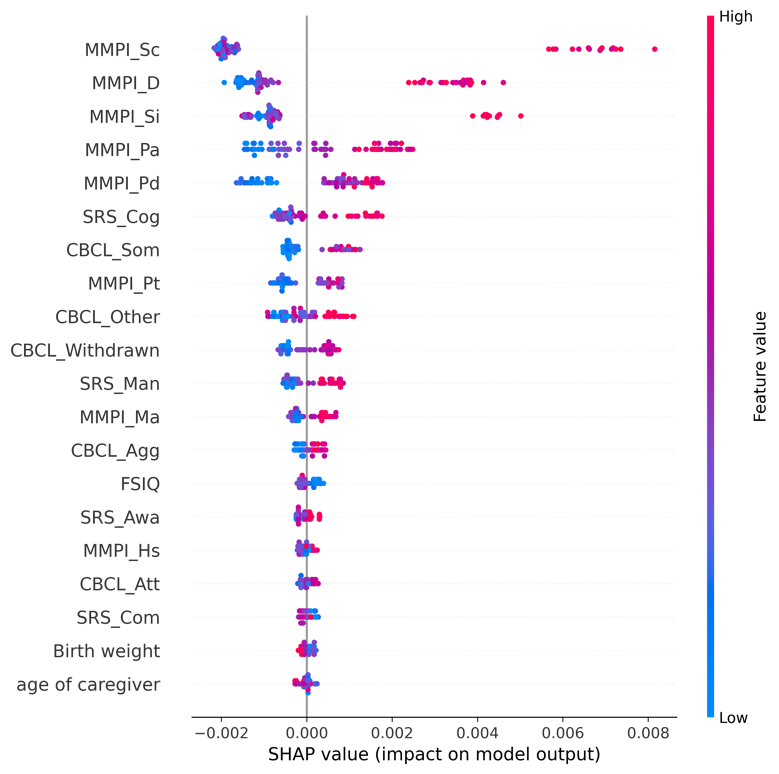 |

## eFigure 22.5. Receiver operating characteristic curves (ROC) for the training and test set

| <Training set> | <Test set> |
| --- | --- |
| 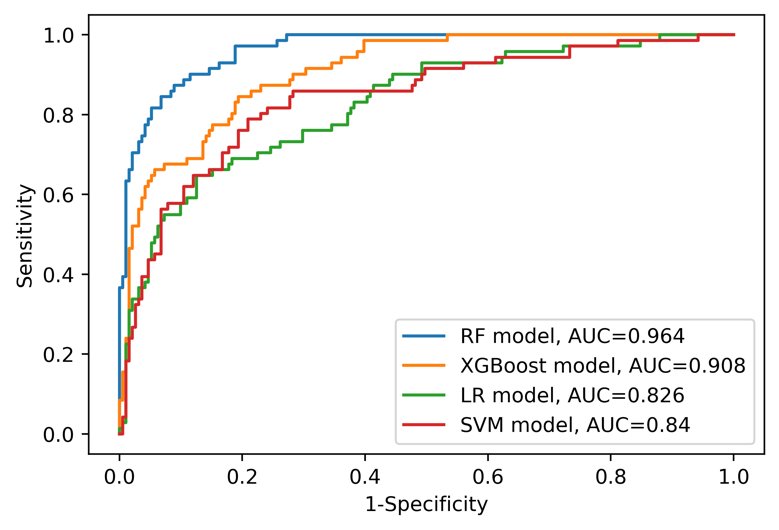 | 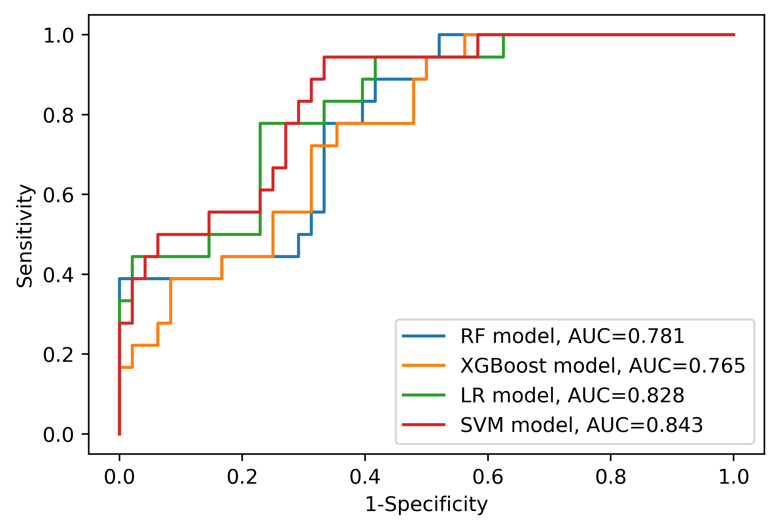 |

Abbreviations: AUC=area under curve, LR=logistic regression, RF=random forest, SVM=support vector machine, XGBoost=Extreme Gradient Boosting.

# **eAppendix 23. Model information and detailed results (Sample: without ADHD [n=328], Outcome: Parent-child dysfunctional interaction)**

## eTable 23.1. Model features (Variables are on the second columns)

| **Caregiver variables** | - Assistant caregiver status [Yes/No]  - Current working status [Yes/No]  - Minnesota Multiphasic Personality Inventory (MMPI) Hypochondriasis (T-score)  - MMPI Depression (T-score)  - MMPI Hysteria (T-score)  - MMPI Psychopathy (T-score)  - MMPI Masculinity/Femininity (T-score)  - MMPI Paranoia (T-score)  - MMPI Psychasthenia (T-score)  - MMPI Schizophrenia (T-score)  - MMPI Hypomania (T-score)  - MMPI Social Introversion (T-score)  - The order of birth  - Number of children |
| --- | --- |
| **ASD patient variables** | - Age  - Sex [Male/Female]  - Family history of mental disorder [Yes/No]  - History of major disease [Yes/No]  - Psychotropic medication status [Drug free/Monotherapy of antipsychotics/Combined therapy of antipsychotics/Other psychotic medication]  - Gestational age  - Birth weight  - Mode of delivery [Vaginal delivery/Caesarean section]  - Existence of another child with mental disorder [Yes/No]  - Full-Scale Intelligence Quotient (FSIQ)  - Social responsiveness scale (SRS) Social awareness (T-score)  - SRS Social cognition (T-score)  - SRS Social communication (T-score)  - SRS Social motivation (T-score)  - SRS Autistic mannerisms (T-score)  - Child Behavior Checklist (CBCL) Anxious/depressed (T-score)  - CBCL Withdrawn/depressed (T-score)  - CBCL Somatic complaints (T-score)  - CBCL Attention problems (T-score)  - CBCL Aggressive behavior (T-score)  - CBCL Other problems (T-score) |

## eTable 23.2. Performance of each model on the training set

|  | **RF model** | **XGBoost model** | **LR model** | **SVM model** |
| --- | --- | --- | --- | --- |
| **ROC AUC (95% CI)** | 0.945 (0.919-0.967) | 0.838 (0.789-0.882) | 0.786 (0.729-0.840) | 0.756 (0.695-0.813) |
| **Sensitivity (95% CI)** | 0.716 (0.628-0.798) | 0.606 (0.510-0.696) | 0.569 (0.476-0.661) | 0.009 (0.000-0.030) |
| **Specificity (95% CI)** | 0.928 (0.886-0.967) | 0.843 (0.784-0.899) | 0.797 (0.732-0.858) | 1.000 (1.000-1.000) |
| **PPV (95% CI)** | 0.876 (0.805-0.941) | 0.733 (0.640-0.824) | 0.667 (0.570-0.761) | NA |
| **NPV (95% CI)** | 0.821 (0.762-0.876) | 0.750 (0.683-0.812) | 0.722 (0.653-0.788) | 0.586 (0.527-0.645) |
| **Accuracy (95% CI)** | 0.840 (0.794-0.882) | 0.744 (0.691-0.794) | 0.702 (0.645-0.756) | 0.588 (0.527-0.645) |
| Abbreviations: AUC=area under curve, CI=confidence interval, NA=not available, NPV=negative predictive value, LR=logistic regression, RF=random forest, ROC=receiver operating characteristic, PPV=positive predictive value, SVM=support vector machine, XGBoost=Extreme Gradient Boosting. | | | | |

## eTable 23.3. Performance of each model on the test set

|  | **RF model** | **XGBoost model** | **LR model** | **SVM model** |
| --- | --- | --- | --- | --- |
| **ROC AUC (95% CI)** | 0.734 (0.605-0.854) | 0.718 (0.587-0.842) | 0.762 (0.637-0.873) | 0.745 (0.616-0.861) |
| **Sensitivity (95% CI)** | 0.630 (0.438-0.815) | 0.667 (0.480-0.846) | 0.667 (0.478-0.846) | 0.000 (0.000-0.000) |
| **Specificity (95% CI)** | 0.718 (0.571-0.857) | 0.744 (0.600-0.875) | 0.744 (0.600-0.875) | 1.000 (1.000-1.000) |
| **PPV (95% CI)** | 0.607 (0.423-0.788) | 0.643 (0.458-0.818) | 0.643 (0.457-0.818) | NA |
| **NPV (95% CI)** | 0.737 (0.591-0.875) | 0.763 (0.622-0.892) | 0.763 (0.622-0.892) | 0.591 (0.470-0.712) |
| **Accuracy (95% CI)** | 0.682 (0.561-0.803) | 0.712 (0.606-0.818) | 0.712 (0.606-0.818) | 0.591 (0.470-0.712) |
| Abbreviations: AUC=area under curve, CI=confidence interval, NA=not available, NPV=negative predictive value, LR=logistic regression, RF=random forest, ROC=receiver operating characteristic, PPV=positive predictive value, SVM=support vector machine, XGBoost=Extreme Gradient Boosting. | | | | |

## eFigure 23.4. SHapley Additive exPlanations (SHAP) summary plot for RF and XGBoost models (only top 20 predictors were presented)

| <RF model> | <XGBoost model> |
| --- | --- |
| 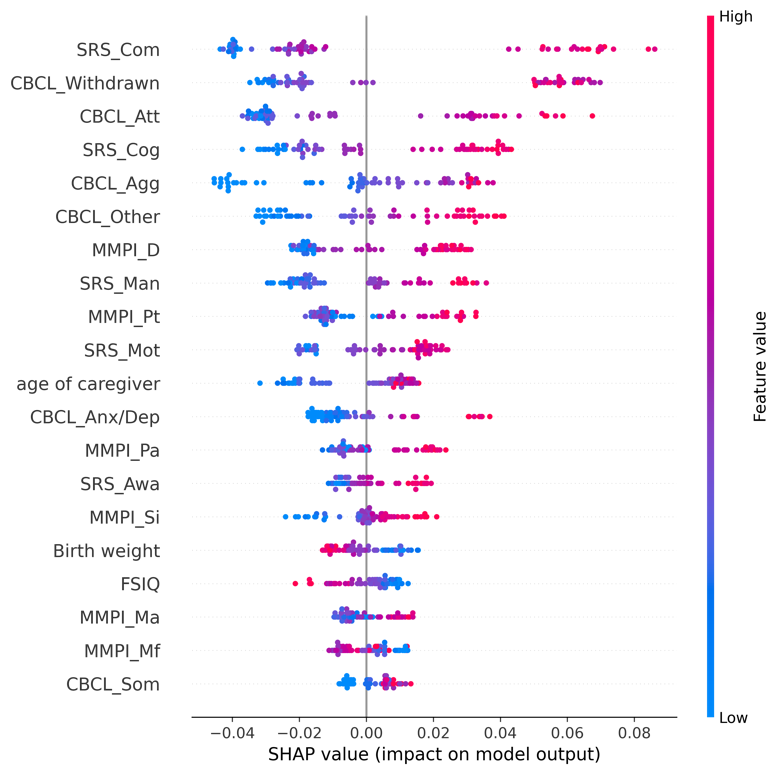 | 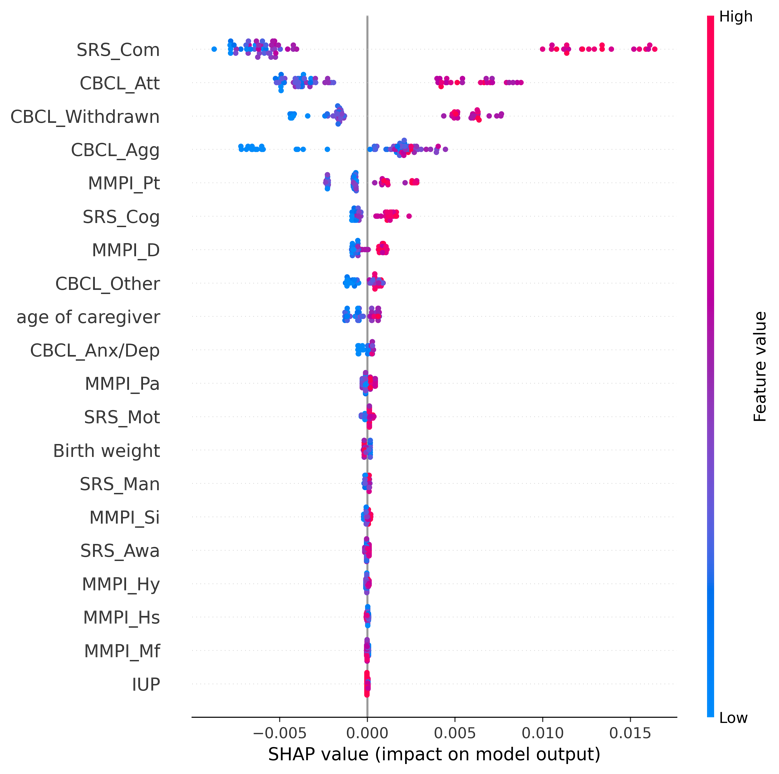 |

## eFigure 23.5. Receiver operating characteristic curves (ROC) for the training and test set

| <Training set> | <Test set> |
| --- | --- |
| 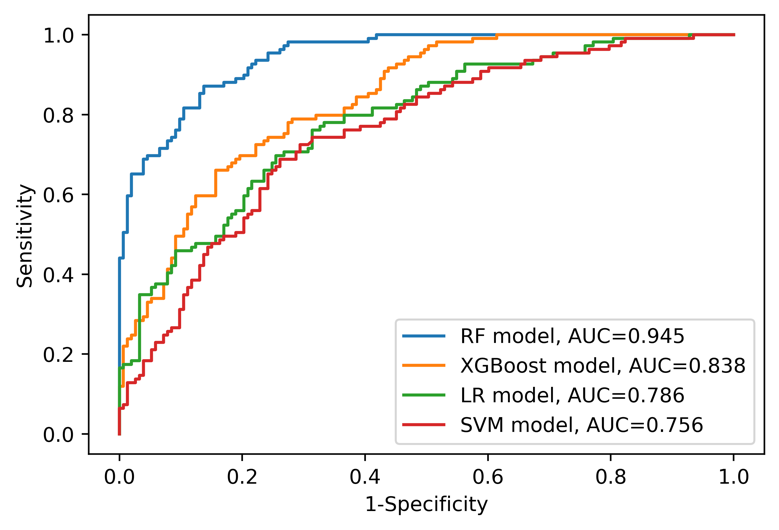 | 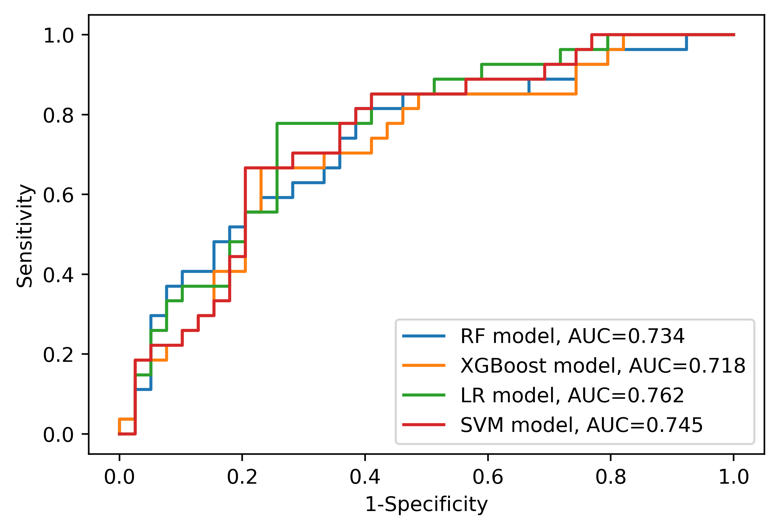 |

Abbreviations: AUC=area under curve, LR=logistic regression, RF=random forest, SVM=support vector machine, XGBoost=Extreme Gradient Boosting.

# **eAppendix 24. Model information and detailed results (Sample: without ADHD [n=328], Outcome: Difficult child)**

## eTable 24.1. Model features (Variables are on the second columns)

| **Caregiver variables** | - Assistant caregiver status [Yes/No]  - Current working status [Yes/No]  - Minnesota Multiphasic Personality Inventory (MMPI) Hypochondriasis (T-score)  - MMPI Depression (T-score)  - MMPI Hysteria (T-score)  - MMPI Psychopathy (T-score)  - MMPI Masculinity/Femininity (T-score)  - MMPI Paranoia (T-score)  - MMPI Psychasthenia (T-score)  - MMPI Schizophrenia (T-score)  - MMPI Hypomania (T-score)  - MMPI Social Introversion (T-score)  - The order of birth  - Number of children |
| --- | --- |
| **ASD patient variables** | - Age  - Sex [Male/Female]  - Family history of mental disorder [Yes/No]  - History of major disease [Yes/No]  - Psychotropic medication status [Drug free/Monotherapy of antipsychotics/Combined therapy of antipsychotics/Other psychotic medication]  - Gestational age  - Birth weight  - Mode of delivery [Vaginal delivery/Caesarean section]  - Existence of another child with mental disorder [Yes/No]  - Full-Scale Intelligence Quotient (FSIQ)  - Social responsiveness scale (SRS) Social awareness (T-score)  - SRS Social cognition (T-score)  - SRS Social communication (T-score)  - SRS Social motivation (T-score)  - SRS Autistic mannerisms (T-score)  - Child Behavior Checklist (CBCL) Anxious/depressed (T-score)  - CBCL Withdrawn/depressed (T-score)  - CBCL Somatic complaints (T-score)  - CBCL Attention problems (T-score)  - CBCL Aggressive behavior (T-score)  - CBCL Other problems (T-score) |

## eTable 24.2. Performance of each model on the training set

|  | **RF model** | **XGBoost model** | **LR model** | **SVM model** |
| --- | --- | --- | --- | --- |
| **ROC AUC (95% CI)** | 0.961 (0.940-0.978) | 0.828 (0.776-0.875) | 0.811 (0.757-0.861) | 0.801 (0.746-0.852) |
| **Sensitivity (95% CI)** | 0.867 (0.802-0.926) | 0.664 (0.575-0.750) | 0.637 (0.549-0.726) | 0.000 (0.000-0.000) |
| **Specificity (95% CI)** | 0.926 (0.881-0.965) | 0.832 (0.771-0.891) | 0.846 (0.785-0.901) | 1.000 (1.000-1.000) |
| **PPV (95% CI)** | 0.899 (0.840-0.951) | 0.750 (0.663-0.832) | 0.758 (0.670-0.841) | NA |
| **NPV (95% CI)** | 0.902 (0.852-0.946) | 0.765 (0.698-0.828) | 0.754 (0.688-0.818) | 0.569 (0.508-0.626) |
| **Accuracy (95% CI)** | 0.901 (0.863-0.935) | 0.760 (0.706-0.809) | 0.756 (0.702-0.805) | 0.569 (0.508-0.626) |
| Abbreviations: AUC=area under curve, CI=confidence interval, NA=not available, NPV=negative predictive value, LR=logistic regression, RF=random forest, ROC=receiver operating characteristic, PPV=positive predictive value, SVM=support vector machine, XGBoost=Extreme Gradient Boosting. | | | | |

## eTable 24.3. Performance of each model on the test set

|  | **RF model** | **XGBoost model** | **LR model** | **SVM model** |
| --- | --- | --- | --- | --- |
| **ROC AUC (95% CI)** | 0.854 (0.755-0.933) | 0.827 (0.717-0.917) | 0.831 (0.720-0.924) | 0.836 (0.726-0.929) |
| **Sensitivity (95% CI)** | 0.714 (0.538-0.875) | 0.679 (0.500-0.846) | 0.679 (0.500-0.846) | 0.000 (0.000-0.000) |
| **Specificity (95% CI)** | 0.842 (0.714-0.949) | 0.789 (0.649-0.917) | 0.816 (0.686-0.930) | 1.000 (1.000-1.000) |
| **PPV (95% CI)** | 0.769 (0.600-0.923) | 0.704 (0.520-0.875) | 0.731 (0.552-0.893) | NA |
| **NPV (95% CI)** | 0.800 (0.667-0.917) | 0.769 (0.632-0.895) | 0.775 (0.641-0.900) | 0.576 (0.455-0.697) |
| **Accuracy (95% CI)** | 0.788 (0.682-0.879) | 0.742 (0.636-0.848) | 0.758 (0.652-0.848) | 0.576 (0.455-0.697) |
| Abbreviations: AUC=area under curve, CI=confidence interval, NA=not available, NPV=negative predictive value, LR=logistic regression, RF=random forest, ROC=receiver operating characteristic, PPV=positive predictive value, SVM=support vector machine, XGBoost=Extreme Gradient Boosting. | | | | |

## eFigure 24.4. SHapley Additive exPlanations (SHAP) summary plot for RF and XGBoost models (only top 20 predictors were presented)

| <RF model> | <XGBoost model> |
| --- | --- |
| 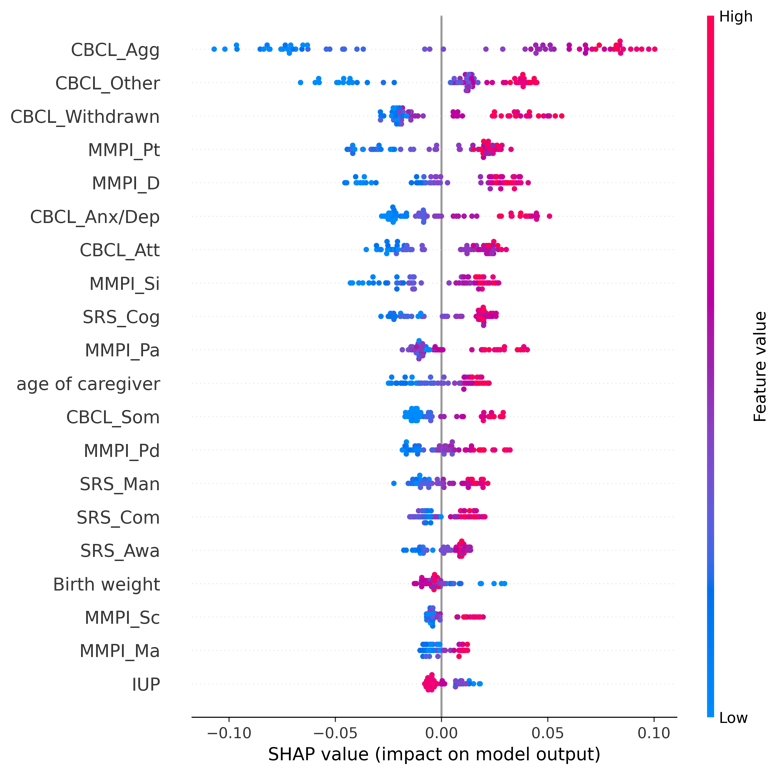 | 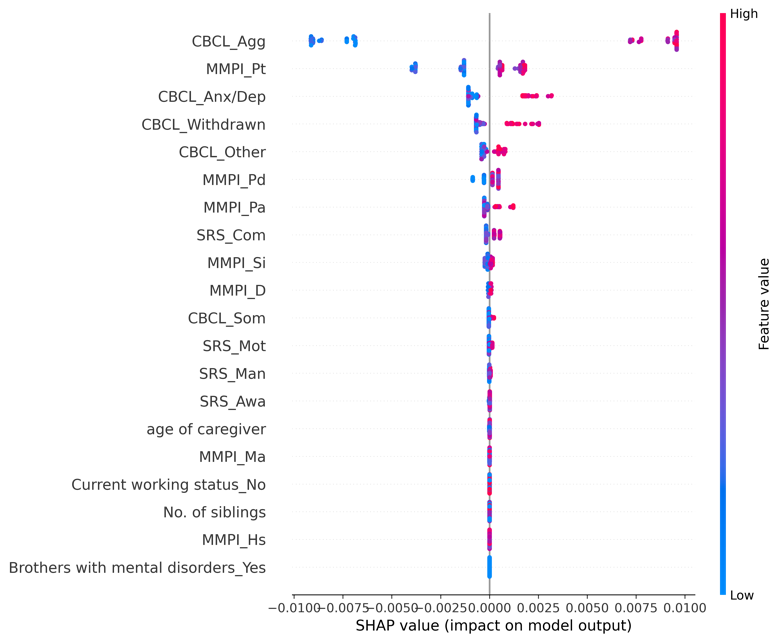 |

## eFigure 24.5. Receiver operating characteristic curves (ROC) for the training and test set

| <Training set> | <Test set> |
| --- | --- |
| 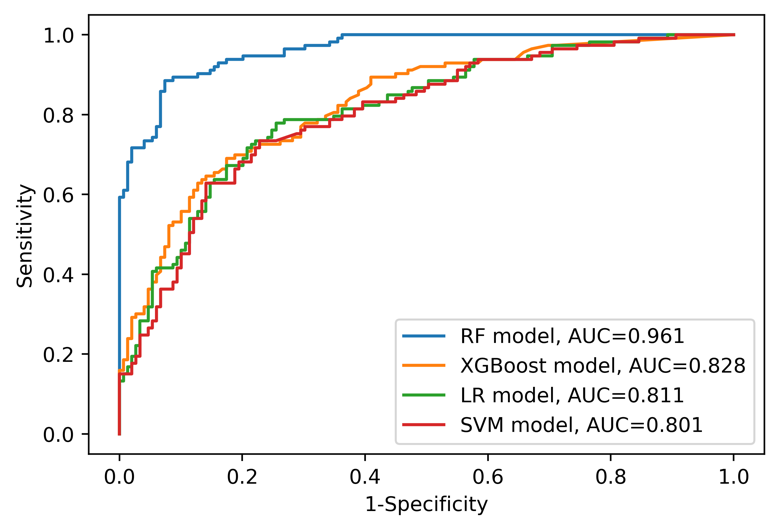 | 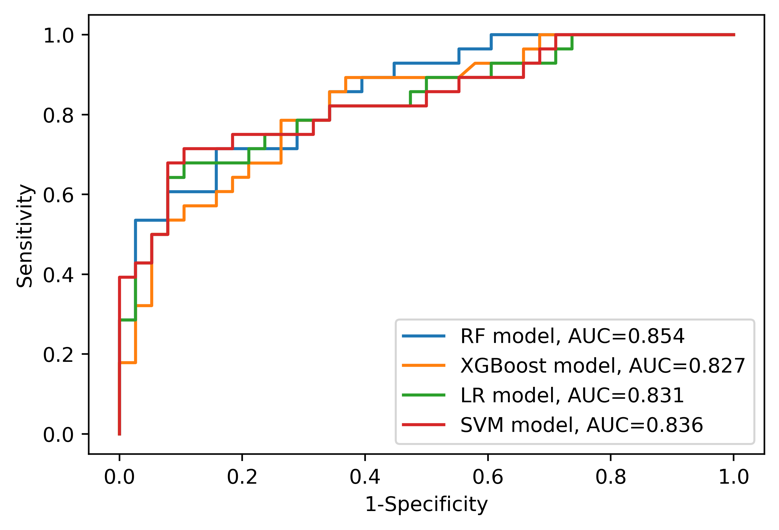 |

Abbreviations: AUC=area under curve, LR=logistic regression, RF=random forest, SVM=support vector machine, XGBoost=Extreme Gradient Boosting.

# **eAppendix 25. Model information and detailed results (Sample: without ADHD [n=328], Outcome: Total parenting stress)**

## eTable 25.1. Model features (Variables are on the second columns)

| **Caregiver variables** | - Assistant caregiver status [Yes/No]  - Current working status [Yes/No]  - Minnesota Multiphasic Personality Inventory (MMPI) Hypochondriasis (T-score)  - MMPI Depression (T-score)  - MMPI Hysteria (T-score)  - MMPI Psychopathy (T-score)  - MMPI Masculinity/Femininity (T-score)  - MMPI Paranoia (T-score)  - MMPI Psychasthenia (T-score)  - MMPI Schizophrenia (T-score)  - MMPI Hypomania (T-score)  - MMPI Social Introversion (T-score)  - The order of birth  - Number of children |
| --- | --- |
| **ASD patient variables** | - Age  - Sex [Male/Female]  - Family history of mental disorder [Yes/No]  - History of major disease [Yes/No]  - Psychotropic medication status [Drug free/Monotherapy of antipsychotics/Combined therapy of antipsychotics/Other psychotic medication]  - Gestational age  - Birth weight  - Mode of delivery [Vaginal delivery/Caesarean section]  - Existence of another child with mental disorder [Yes/No]  - Full-Scale Intelligence Quotient (FSIQ)  - Social responsiveness scale (SRS) Social awareness (T-score)  - SRS Social cognition (T-score)  - SRS Social communication (T-score)  - SRS Social motivation (T-score)  - SRS Autistic mannerisms (T-score)  - Child Behavior Checklist (CBCL) Anxious/depressed (T-score)  - CBCL Withdrawn/depressed (T-score)  - CBCL Somatic complaints (T-score)  - CBCL Attention problems (T-score)  - CBCL Aggressive behavior (T-score)  - CBCL Other problems (T-score) |

## eTable 25.2. Performance of each model on the training set

|  | **RF model** | **XGBoost model** | **LR model** | **SVM model** |
| --- | --- | --- | --- | --- |
| **ROC AUC (95% CI)** | 0.990 (0.981-0.996) | 1.000 (1.000-1.000) | 0.878 (0.833-0.918) | 0.878 (0.835-0.917) |
| **Sensitivity (95% CI)** | 0.910 (0.855-0.959) | 0.984 (0.958-1.000) | 0.754 (0.677-0.829) | 0.754 (0.675-0.829) |
| **Specificity (95% CI)** | 0.971 (0.941-0.993) | 1.000 (1.000-1.000) | 0.871 (0.812-0.925) | 0.850 (0.788-0.906) |
| **PPV (95% CI)** | 0.965 (0.929-0.992) | 1.000 (1.000-1.000) | 0.836 (0.764-0.903) | 0.814 (0.741-0.883) |
| **NPV (95% CI)** | 0.925 (0.879-0.966) | 0.986 (0.964-1.000) | 0.803 (0.737-0.865) | 0.799 (0.732-0.862) |
| **Accuracy (95% CI)** | 0.943 (0.912-0.969) | 0.992 (0.981-1.000) | 0.817 (0.771-0.863) | 0.805 (0.756-0.851) |
| Abbreviations: AUC=area under curve, CI=confidence interval, NA=not available, NPV=negative predictive value, LR=logistic regression, RF=random forest, ROC=receiver operating characteristic, PPV=positive predictive value, SVM=support vector machine, XGBoost=Extreme Gradient Boosting. | | | | |

## eTable 25.3. Performance of each model on the test set

|  | **RF model** | **XGBoost model** | **LR model** | **SVM model** |
| --- | --- | --- | --- | --- |
| **ROC AUC (95% CI)** | 0.759 (0.636-0.869) | 0.755 (0.630-0.868) | 0.755 (0.633-0.864) | 0.753 (0.629-0.864) |
| **Sensitivity (95% CI)** | 0.742 (0.579-0.889) | 0.742 (0.576-0.892) | 0.710 (0.545-0.865) | 0.677 (0.500-0.84) |
| **Specificity (95% CI)** | 0.629 (0.464-0.788) | 0.657 (0.500-0.812) | 0.657 (0.500-0.812) | 0.657 (0.500-0.806) |
| **PPV (95% CI)** | 0.639 (0.476-0.795) | 0.657 (0.500-0.812) | 0.647 (0.484-0.806) | 0.636 (0.467-0.795) |
| **NPV (95% CI)** | 0.733 (0.568-0.889) | 0.742 (0.581-0.889) | 0.719 (0.556-0.871) | 0.697 (0.536-0.853) |
| **Accuracy (95% CI)** | 0.682 (0.561-0.788) | 0.697 (0.591-0.803) | 0.682 (0.576-0.788) | 0.667 (0.545-0.773) |
| Abbreviations: AUC=area under curve, CI=confidence interval, NA=not available, NPV=negative predictive value, LR=logistic regression, RF=random forest, ROC=receiver operating characteristic, PPV=positive predictive value, SVM=support vector machine, XGBoost=Extreme Gradient Boosting. | | | | |

## eFigure 25.4. SHapley Additive exPlanations (SHAP) summary plot for (only top 20 predictors were presented)

| <RF model> | <XGBoost model> |
| --- | --- |
| 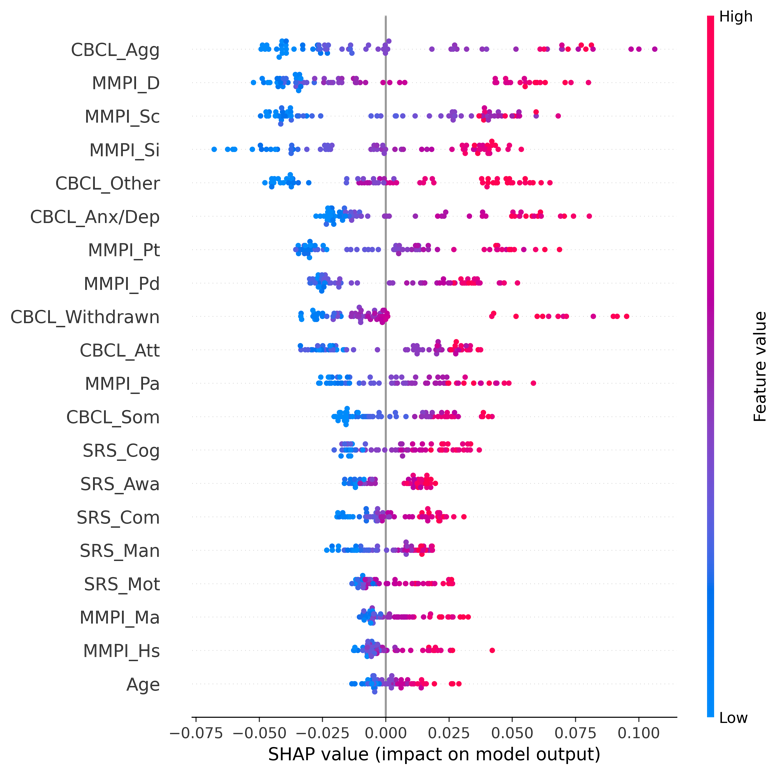 | 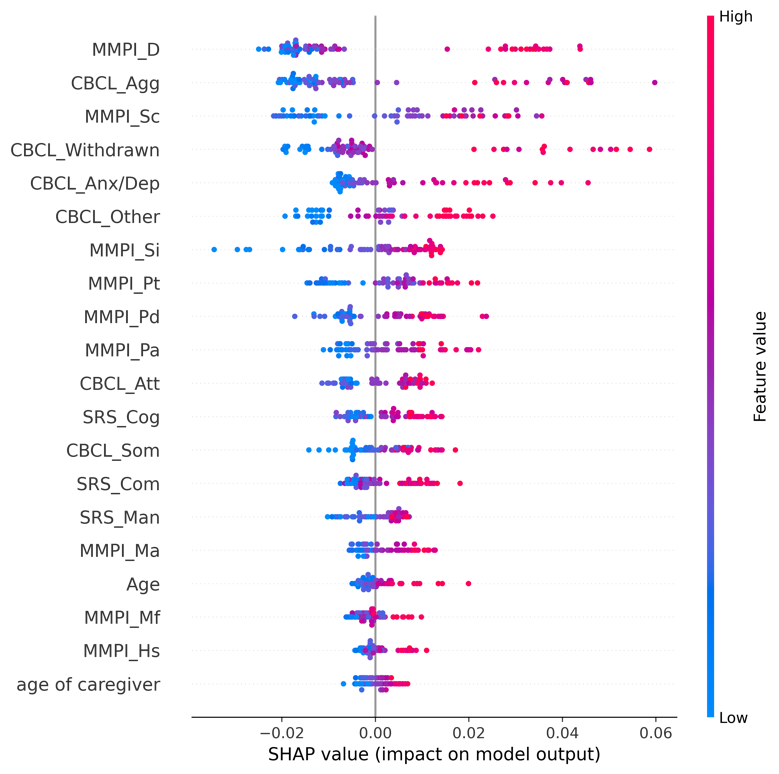 |

## eFigure 25.5. Receiver operating characteristic curves (ROC) for the training and test set

| <Training set> | <Test set> |
| --- | --- |
| 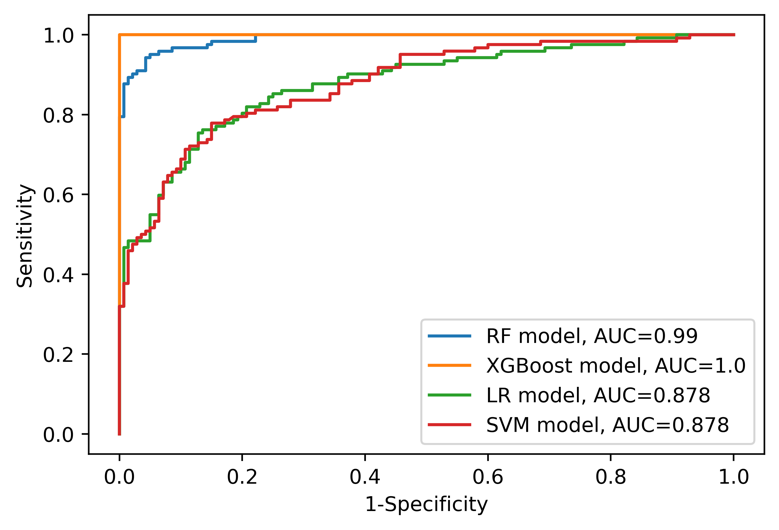 | 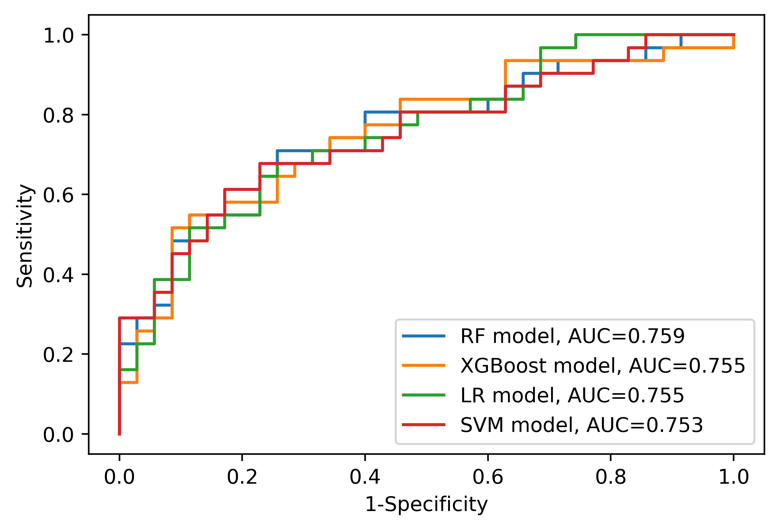 |

Abbreviations: AUC=area under curve, LR=logistic regression, RF=random forest, SVM=support vector machine, XGBoost=Extreme Gradient Boosting.
